# A salt-induced kinase is required for the metabolic regulation of sleep

**DOI:** 10.1371/journal.pbio.3000220

**Published:** 2020-04-21

**Authors:** Jeremy J. Grubbs, Lindsey E. Lopes, Alexander M. van der Linden, David M. Raizen

**Affiliations:** 1 Department of Biology, University of Nevada, Reno, Nevada, United States of America; 2 Department of Neurology, Perelman School of Medicine, University of Pennsylvania, Philadelphia, Pennsylvania, United States of America; University of Massachusetts Medical School, UNITED STATES

## Abstract

Many lines of evidence point to links between sleep regulation and energy homeostasis, but mechanisms underlying these connections are unknown. During *Caenorhabditis elegans* sleep, energetic stores are allocated to nonneural tasks with a resultant drop in the overall fat stores and energy charge. Mutants lacking KIN-29, the *C*. *elegans* homolog of a mammalian Salt-Inducible Kinase (SIK) that signals sleep pressure, have low ATP levels despite high-fat stores, indicating a defective response to cellular energy deficits. Liberating energy stores corrects adiposity and sleep defects of *kin-29* mutants. *kin-29* sleep and energy homeostasis roles map to a set of sensory neurons that act upstream of fat regulation as well as of central sleep-controlling neurons, suggesting hierarchical somatic/neural interactions regulating sleep and energy homeostasis. Genetic interaction between *kin-29* and the histone deacetylase *hda-4* coupled with subcellular localization studies indicate that KIN-29 acts in the nucleus to regulate sleep. We propose that KIN-29/SIK acts in nuclei of sensory neuroendocrine cells to transduce low cellular energy charge into the mobilization of energy stores, which in turn promotes sleep.

## Abstract 2

Sleep is intricately connected with metabolism. This study shows that KIN-29, the orthologue of the mammalian salt-inducible kinase (SIK) in the nematode *Caenorhabditis elegans*, is a key regulator involved in connecting sleep and energy homeostasis.

## Introduction

Sleep is intricately connected with metabolism, and reciprocal interactions between sleep and metabolic processes underlie a number of clinical pathologies. Acute disruption of human sleep results in elevated appetite [1] and insulin resistance [2], and chronically short-sleeping humans are more likely to be obese and diabetic [3]. Starvation in humans, rats, *Drosophila*, and *C*. *elegans* affects sleep [4–9], indicating that sleep is regulated, in part, by nutrient availability.

Sleep is associated with reduced neural energetic demands across phylogeny [10–12]; for example, slow-wave sleep in mammals is associated with reduced nervous system energetic demands [13–15], and the reduction in neural activity in a sleeping *C*. *elegans* is likely similarly associated with reduced energy demands [11]. Despite this apparent reduced energy demand in neurons, overall metabolic rates during sleep in mammals [15,16] and *Drosophila* [17] are only modestly reduced, suggesting that during sleep, energetic stores are allocated to other metabolic functions [18], such as the synthesis of proteins [19] and other macromolecules [20]. Importantly, although there are genes reported to function in the regulation of both metabolism and sleep [21–26], mechanisms by which these gene products couple the 2 processes at the level of the whole organism remain unclear. Thus, the molecular and cellular mechanisms connecting sleep with energy homeostasis of the animal remain opaque.

Salt-Inducible Kinases (SIKs) have been identified as conserved regulators of sleep [27] and metabolism [28]. There are 3 SIKs in mammals, 2 in *Drosophila*, and 1 in *C*. *elegans* called KIN-29 [29]. Gain-of-function mouse mutants of SIK3 are sleepy [27] with a phosphoprotein profile that mimics that of sleep-deprived mice [30], suggesting that SIK3 signaling promotes sleep need. The *Drosophila* SIK3 and *C*. *elegans* KIN-29 loss-of-function mutants have reduced sleep [27]. *kin-29* is also required for sleep in satiated animals [31,32], suggesting a generalized role for KIN-29 in promoting sleep.

In addition to sleep behavioral phenotypes, SIK gene mutations are associated with metabolic defects. In *Drosophila*, reduction of dSIK gene function in neurons results in elevated levels of triglycerides and glycogen [33], whereas loss of *Drosophila* SIK3 in the fat body results in a depletion of triglyceride stores [34]. Based on this combination of sleep and metabolic phenotypes as well as on our preliminary studies, we hypothesized that SIKs’ function may be an integral part of the mechanism by which sleep and energy homeostasis are integrated. We set out to test this hypothesis using *C*. *elegans*, which has proven a powerful organism to study sleep [35], as well as metabolism [36].

*C*. *elegans* sleeps during development in a stage known as developmentally timed sleep (DTS), or lethargus [37,38]. They also sleep after exposure to environmental conditions that cause cellular stress in a behavior termed stress-induced sleep (SIS) [39,40]. Additionally, *C*. *elegans* sleep when satiated [32,41] and in the setting of starvation [4,32]. Two neurons show strong effects in regulating *C*. *elegans* sleep: the RIS neuron regulates DTS [42], SIS [43], starvation-associated sleep [32], and satiety-associated sleep [32], and the ALA neuron regulates SIS [39,44,45].

Here, we show that multiple types of *C*. *elegans* sleep are associated with reduced energy levels of the animals and require the function of KIN-29/SIK. Consistent with a deficit in energy mobilization, we show that *kin-29* mutants have reduced ATP and behave like starved animals despite having elevated fat stores. Experimental mobilization of triglycerides in these fat stores restores sleep. We find that *C*. *elegans kin-29* acts in the nucleus to regulate sleep and energy homeostasis via *hda-4*, which encodes a class IIa histone-deacetylase. KIN-29 and HDA-4 act in the same metabolically responsive sensory neurons upstream of or in parallel to fat homeostasis and to the activation of the sleep-promoting neurons ALA and RIS. Together, these results indicate that sleep is regulated via hierarchical interactions between neurons that sense energy needs, fat-storage cells that respond to energy need, and the action of sleep-inducing neurons.

## Results

### Sleep is associated with energetic store depletion and fat mobilization

To understand how sleep/wake states are coordinated with the metabolic states of an animal, we studied how lethargus (DTS), SIS, and sleep deprivation correlate with metabolic measurements. We hypothesized that during lethargus/DTS, when *C*. *elegans* faces the energetically expensive task of replacing its exoskeleton [46], it sleeps to conserve energy [15] and also mobilizes fat to release energy for biosynthetic tasks. Likewise, during conditions of cellular stress, such as after genotoxic ultraviolet light exposure, *C*. *elegans* sleeps presumably to conserve energy and mobilizes fat to release energy needed for cellular repair. If this hypothesis is correct, then energy levels should be inversely proportional to sleep drive. When energy levels are low or dropping, sleep drive is high, and when energy levels are high or increasing, sleep drive is low.

**Fig 1:**
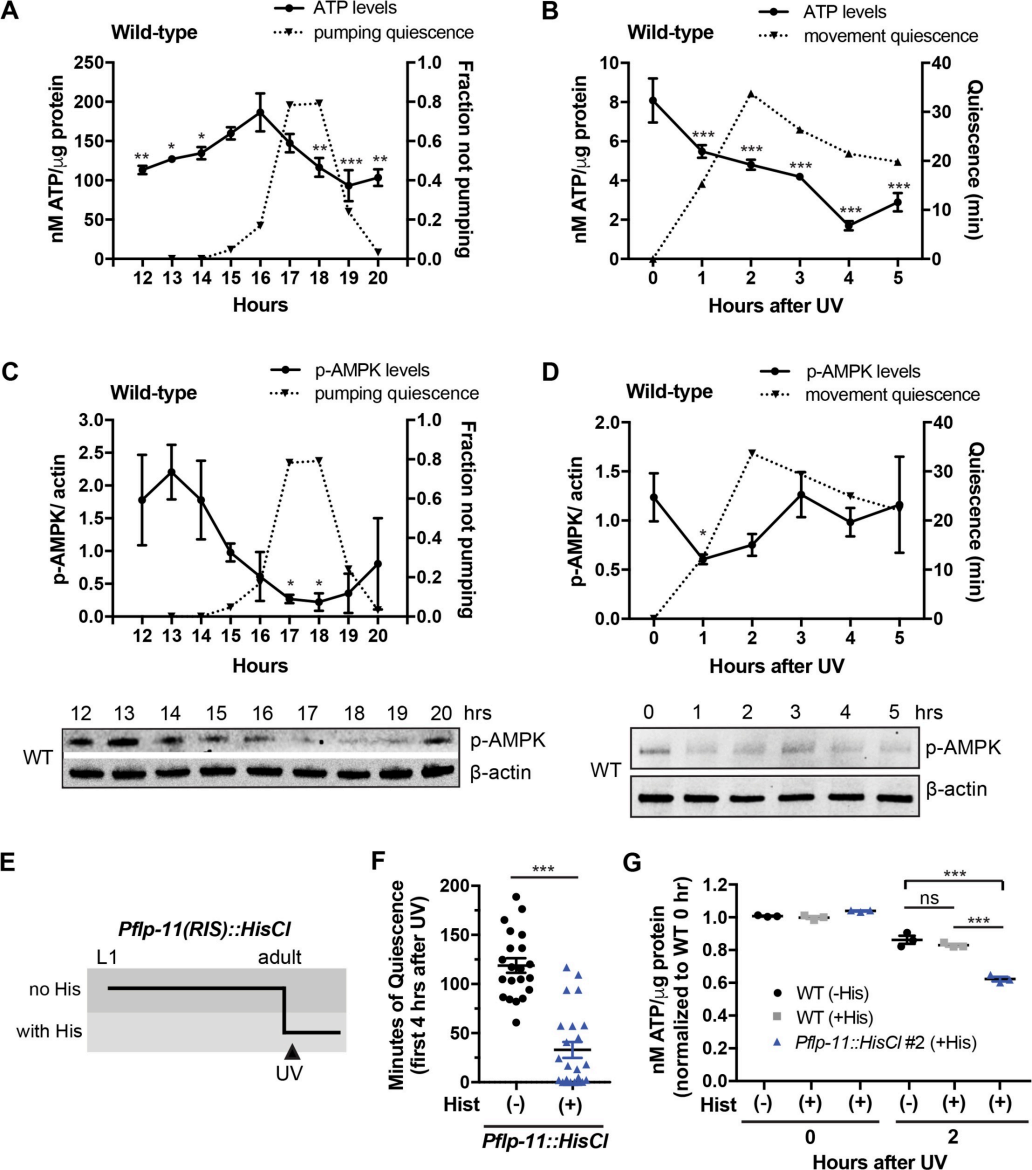
ATP and p-AMPK levels fall during DTS and SIS. (A and C) Total body ATP normalized by μg protein (A) and total body p-AMPK normalized to actin loading control (C) in WT animals before, during, and after lethargus/DTS of the L1 stage. Shown is a representative time course performed in technical duplicates of an experiment that was replicated 6 times for ATP levels (the average of the 7 biological replications is shown in Fig 3F) and 3 times for p-AMPK staining (Fig 3G). p-AMPK and ATP levels were measured from worms grown in the same batch. Data are represented as the mean ± SD with 2 technical replicates for ATP and AMPK. The second y-axis shows the averaged fraction of nonpumping animals (*n* = 10) for each time point. Statistical comparisons were performed with a 2-way ANOVA, followed by post hoc pairwise comparisons at each time point to obtain nominal *p*-values, which were subjected to a Tukey multiple-comparison correction. ATP values that are significantly different from the largest value in the time course are indicated by *** (*p* < 0.001), ** (*p* < 0.01), and * (*p* < 0.05) (S1 Data, Sheet 1A and 1C). A representative western blot is shown below the graph, in which the intensities of the upper bands represent levels of p-AMPK using an antibody against mammalian p-AMPKα Thr-172, and the intensities of the lower bands represent levels of the β-actin loading control. (B and D) Total body ATP normalized by μg protein (B) and total body p-AMPK normalized to actin loading control (D) as well as movement quiescence in WT adults after UVC irradiation. We measured body movement quiescence of animals that were in the same batch used for ATP measurements. Shown is a representative time course of an experiment run independently 6 times for ATP (Fig 3H) and 3 times for p-AMPK staining (Fig 3I). Data are represented as the mean ± SD with 3 technical replicates for ATP. For p-AMPK, data are represented as the mean ± SEM of 2 experiments. The second y-axis shows the averaged movement quiescence of animals (*n* = 5–7) for each time point. Statistical comparisons were performed with a 2-way ANOVA, followed by post hoc pairwise comparisons at each time point to obtain nominal *p*-values, which were subjected to a Tukey multiple-comparison correction. *** and * indicate corrected *p*-values that are different from the 0-hr time point at *p* < 0.001 and *p* < 0.05, respectively (S1 Data, Sheet 1B and 1D). A representative western blot of p-AMPK and actin levels is shown below the graph. (E) Schematic of a histamine-mediated chemogenetic inhibition experiment to deprive worms of SIS. Age-matched worms were grown from the L1 stage to adulthood in the absence of histamine. One-day-old adults were transferred to individual wells of a WorMotel device with each well containing 10 mM histamine or vehicle control agar. Approximately 15 min after transfer, worms were exposed to UVC irradiation (1,500 J/m^2^), and their body movement quiescence was recorded for 8 hr. See also Material and methods. (F) Minutes of body movement quiescence during the first 4 hr after UVC exposure/SIS in adult animals expressing either the *Pflp-11*::*HisCl* transgene (+) or in nontransgenic animals (-). Shown is the combined data of 2 biological replicates as shown in S2B Fig. Data are represented as mean ± SEM. ***p < 0.001 by a 2-tailed Mann-Whitney *t* test (S1 Data, Sheet 1F). (G) Total body ATP per μg protein measured 0 and 2 hr (during maximal movement quiescence) after UVC exposure in adult animals expressing either the *Pflp-11*::*HisCl* transgene or in nontransgenic animals in the presence of 10 mM histamine (+His) compared with WT adults in the absence of histamine (-His). Data were normalized to WT controls immediately before UVC exposure (0 hr) in the absence of histamine (-His). The graph shows the mean ± SEM of 3 experiments. *** indicates values that are different from that of nontransgenic animals (+His) at *p* < 0.001, and ns indicates *p* > 0.05. Statistical comparisons were performed with a 2-way ANOVA using time and genotype as factors, followed by post hoc pairwise comparisons at each time point to obtain nominal *p*-values, which were subjected to a Tukey correction for multiple comparisons (S1 Data, Sheet 1G). AMPK, adenosine monophosphate regulated protein kinase; DTS, developmentally timed sleep; L1 stage, first larval stage; ns, not significant; p-AMPK, phosphorylated AMPK; SIS, stress-induced sleep; UVC, ultraviolet C; WT, wild type.

To assess energy levels, we measured ATP in whole animals. Consistent with published data [47], ATP levels built up until first larval stage (L1) lethargus/DTS, dropped during L1 lethargus, and then began to recover after L1 lethargus (Fig 1A). Although we normalized ATP levels to total protein, the drop in the levels is not explained by a concomitant increase in protein levels (S1A Fig). Similarly, after exposure to genotoxic stress, ATP levels decreased for 4 hr, a period in which the animals slept (Fig 1B). These data suggest that during SIS and DTS, the rate of ATP consumption exceeds the rate of ATP generation. We note that absolute levels of ATP were low not only during L1 lethargus (16–19 hr) but also at the 12-hr time point, when the animals were awake and moving. Hence, within the limits of our time window of observation, quiescent behavior appears to be associated with dropping ATP levels and not necessarily with the absolute levels of ATP (S1C Fig).

Adenosine monophosphate regulated protein kinase (AMPK) is a conserved regulator of metabolism and energy at the cellular and whole body level [48]. AMPK is activated by a high ratio of AMP to ATP; its activation inhibits anabolic pathways and activates catabolic pathways [48]. We measured activation of the *C*. *elegans* AMPKα2 homolog AAK-2 using an antibody directed against the phosphorylated threonine 172 of mammals AMPK, which is equivalent to threonine 243 in *C*. *elegans* AAK-2 (S1D Fig). This anti–phosphorylated AMPK (p-AMPK) antibody detected a 72-kilodalton band in wild-type *C*. *elegans*, which was absent in animals carrying an *aak-2* null mutation (S1D Fig), as shown previously [49]. Consistent with the known phosphorylation of AMPK in the setting of high AMP/ATP ratios, ATP levels were low (S1E Fig) and p-AMPK levels were high (S1F Fig) in animals mutant for Pten induced putative kinase 1 (*pink-1*), the *C*. *elegans* PARK6 homolog, which is required for mitochondria maintenance [50]. These experiments demonstrate the specificity of the antibody for phosphorylated AAK-2 and the reliability of p-AMPK in reporting the anabolic versus catabolic state of the animal.

Surprisingly, despite the falling ATP levels measured during DTS and SIS, p-AMPK was decreased during lethargus/DTS as well as during SIS (Fig 1C and 1D). These results suggest that AMPK is not activated in whole animals during these sleep states despite falling whole animal ATP levels, suggesting that the animal is in an overall anabolic metabolic state. However, we cannot exclude the possibility that AMPK is activated in specific cells.

Our ATP measurements show that sleep behavior correlates with dropping ATP levels. However, it remained unclear whether sleep behavior causes the ATP drop or whether sleep is in response to ATP depletion—i.e., sleep is an attempt by the animal to conserve energy. To distinguish between these possibilities, we developed a chemogenetic approach to sleep deprive animals. We expressed a histamine-gated chloride channel (HisCl) in the sleep-promoting RIS neuron [42] and then cultivated animals on histamine during sleep. This approach works because histamine is not used by *C*. *elegans* as a neurotransmitter [51]. RIS is required for movement quiescence during lethargus/DTS and, in addition, is required for quiescence during UV-induced SIS (S11C Fig) as well as heat-induced SIS [43] (S11D Fig). Chemogenetic silencing of RIS resulted in a 35.0% ± 10.1% (mean ± SEM, *n* = 6) reduction in DTS (S2A Fig) and 72.3% ± 9.5% (mean ± SEM, *n* = 12) reduction in SIS (Fig 1E and 1F; S2B Fig). Most of the reduction in DTS quiescence in response to HisCl activation occurred in the second half of lethargus, suggesting that this period is more sensitive to RIS inhibition.

We compared ATP levels during SIS of animals in which sleep was chemogenetically reduced with animals expressing the HisCl but not exposed to histamine. There was no effect of histamine itself on ATP levels. ATP levels were reduced specifically by preventing sleep (Fig 1G and S2C Fig). These data suggest that the sleep state is not causing the drop in ATP levels; rather, it is a response to increased energetic demands, in an attempt to conserve energy. p-AMPK levels remained low with sleep deprivation (S2D Fig), suggesting that they are regulated independently from sleep behavior.

**Fig 2:**
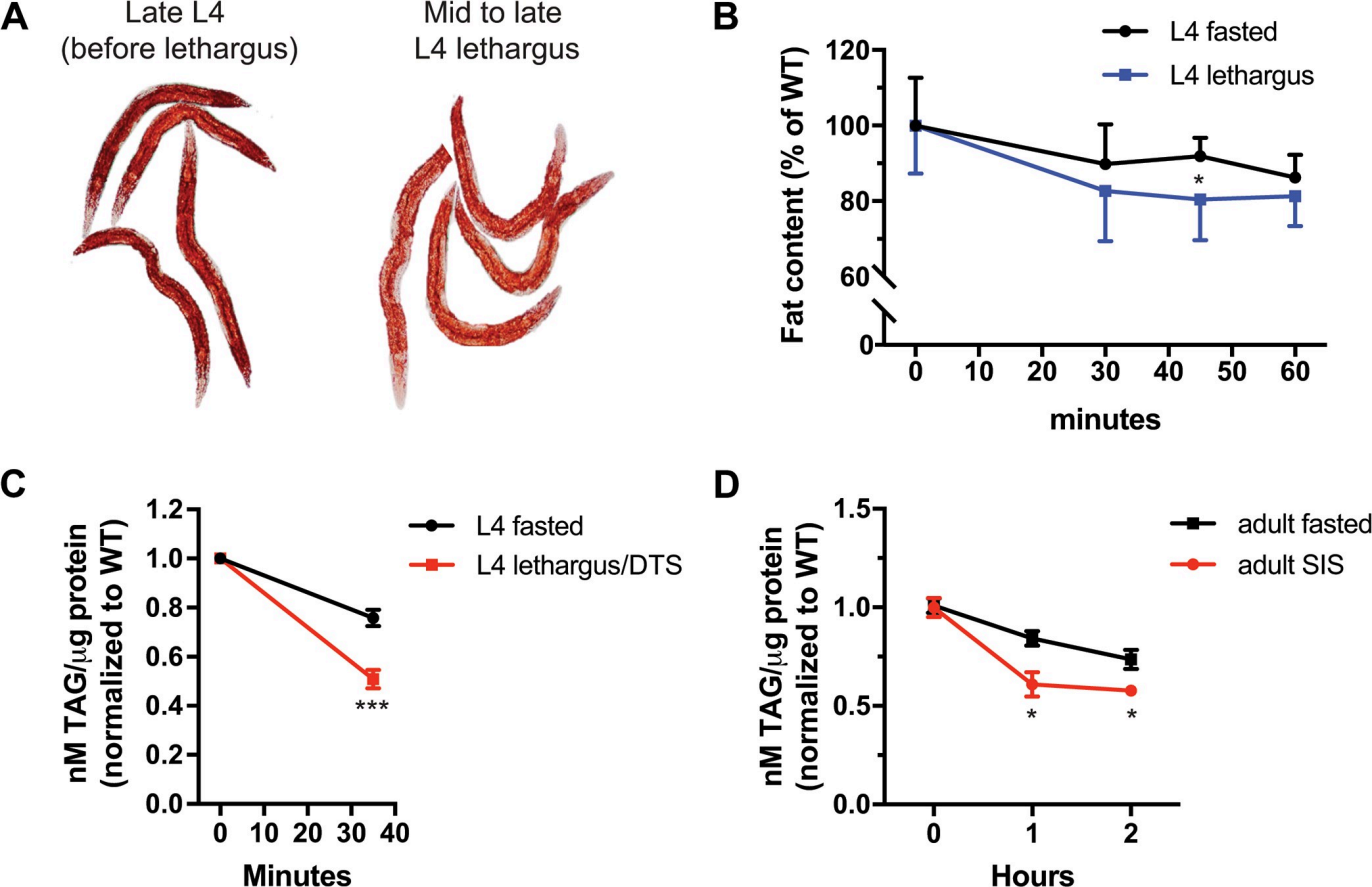
Fat stores are depleted during DTS lethargus and SIS. (A) Representative images of Oil Red O–stained L4 larvae before lethargus (left image) and during mid to late lethargus (right image). (B) Fat content measured with fixative Oil Red O staining of WT L4 larvae during nonlethargus L4 fasting and during lethargus/DTS (*n* = 5–15 animals per time point). Fat levels during lethargus/DTS are depleted faster than fat levels of fasting nonlethargus animals. Data are represented as a percentage of nonstarved awake animals ± SEM. *p < 0.05. Statistical comparisons were performed with a mixed-effects analysis using time and conditions as factors, followed by post hoc pairwise comparisons at each time point to obtain nominal *p*-values, which were subjected to a Bonferroni correction for multiple comparisons (S1 Data, Sheet 2B). (C and D) Total triglyceride (TAG) per μg protein measured in (C) fasting WT L4 larvae during nonlethargus and during lethargus (*n* = 35 animals per time point), and (D) in WT adult animals during fasting and after exposure to UVC irradiation (1,500 J/m^2^) (SIS). For DTS, animals were collected 35 min after onset of lethargus or starvation. Data were normalized to WT controls (0 hr) for each condition. Graphs show the mean ± SEM of 2 experiments with *n* = 4–6 technical replicates for each time point. *** and * indicate values that are different from that of fasted animals at *p* < 0.001 and *p* < 0.05, respectively. Statistical comparisons were performed with a 2-way ANOVA using time and conditions as factors, followed by a Bonferroni correction for multiple comparisons (S1 Data, Sheet 2C and 2D). DTS, developmentally timed sleep; L4, fourth larval stage; SIS, stress-induced sleep; TAG, triacylglyceride; UVC, ultraviolet C; WT, wild type.

When subjected to reduced nutrient intake or to increased nutrient expenditure, animals break down fat stores to release energy for use by all cells. Because during lethargus/DTS, *C*. *elegans* synthesize and secrete a cuticle [46], an energetically expensive task, we asked whether fat levels during DTS were depleted faster than can be explained by fasting alone. In support of the high energetic demands during lethargus/DTS, we observed a depletion of fat stores using 2 different methods to quantify fat—Oil Red O staining and total triglyceride measurements. When we fasted awake animals for an hour during the middle of the fourth larval (L4) stage, their fat stores decreased, but animals in lethargus/DTS showed a greater reduction of fat stores (Fig 2A–2C), suggesting that energetic demands are indeed increased during lethargus/DTS. Similarly, fat stores were depleted faster during SIS than during fasting (Fig 2D). Thus, a reduction in energy use in the neuromuscular system by sleeping does not fully compensate for the overall energetic demands during DTS and SIS.

Together, these results indicate that *C*. *elegans* sleep, both during DTS and during SIS, is associated with a net negative energy balance, in which ATP consumption is greater than ATP synthesis, and that sleep is an energy-conserving state.

### KIN-29 regulates sleep, metabolic stores, and energy charge

We sought to identify molecular signals that mediate this metabolic regulation of sleep. Because SIK3 is a key regulator of sleep [27] and metabolism [34,52,53], we reasoned that SIKs may coordinate both metabolism and behavioral state (sleep/wake). In contrast to mammals or *Drosophila*, which have 3 or 2 genes, respectively, encoding SIK proteins, *C*. *elegans* has only 1 called KIN-29 (S3A Fig), thereby simplifying genetic manipulations.

**Fig 3:**
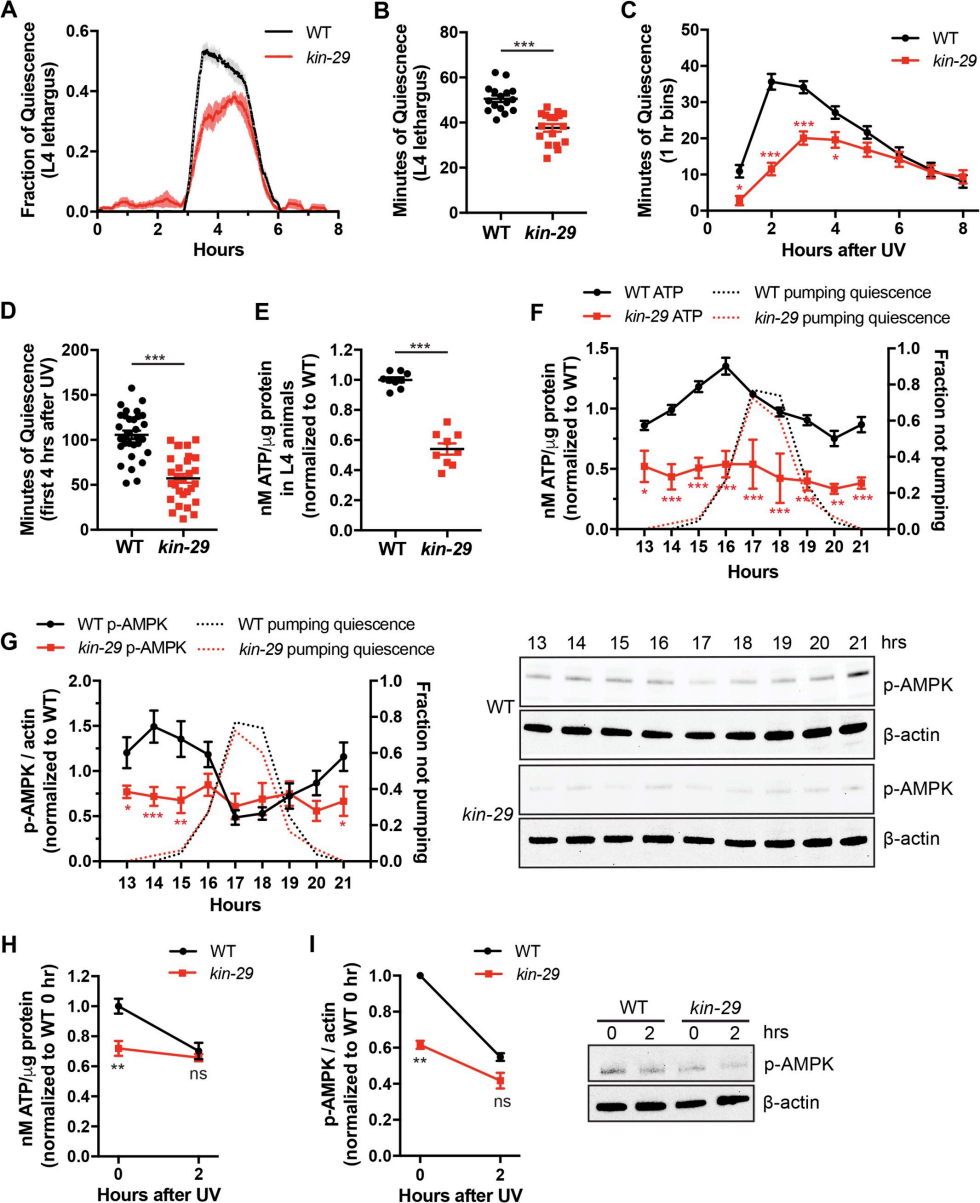
*kin-29* mutants have reduced DTS and SIS and low ATP and p-AMPK levels. (A) Fraction of body movement quiescence of WT and *kin-29* null mutant animals. Data are represented as a moving window of the fraction of a 10-min time interval spent quiescent of *n* = 6 animals for each trace. WT and *kin-29* mutants were analyzed on the same WorMotel. The x-axis represents hours from the start of recording in the late L4 stage. The data from individual worms were aligned such that the start of lethargus quiescence occurred simultaneously. Shading indicates SEM (S1 Data, Sheet 3A). (B) Movement quiescence during L4 lethargus/DTS is lower in *kin-29* null mutants compared with WT (*n* = 16–17 animals combined from 3 separate experiments). Data are represented as the mean ± SEM. ***p < 0.001 by an unpaired 2-tailed *t* test (S1 Data, Sheet 3B). (C) Time course of body movement quiescence per hour for WT and *kin-29* null mutant animals (*n* = 32 animals for each genotype) after UVC irradiation (1,500 J/m^2^). Graphs show the mean ± SEM. Statistical comparisons were performed with a 2-way ANOVA using time and genotype as factors, followed by post hoc pairwise comparisons at each time point to obtain nominal *p*-values, which were subjected to a Bonferroni correction for multiple comparisons. *** and * indicate corrected *p*-values that are different from WT at *p* < 0.001 and *p* < 0.05, respectively (S1 Data, Sheet 3C). (D) Minutes of body movement quiescence during the first 4 hr after UVC exposure/SIS is lower in *kin-29* null mutants compared with WT (*n* = 32 animals for each genotype) as determined from the time-course data in (C). Data are represented as mean ± SEM. ***p < 0.001 by an unpaired 2-tailed *t* test (S1 Data, Sheet 3D). (E) Total body ATP levels per μg protein are lower in *kin-29* L4 larvae compared with WT mid-L4 larvae. Data are normalized to WT and represented as the mean ± SEM of 9 experiments. ***p < 0.001 by an unpaired 2-tailed *t* test (S1 Data, Sheet 3E). (F) L1 time course of total body ATP levels per μg protein in WT and *kin-29* null mutant animals. Data are normalized to the average value of the WT time course. Graphs show the mean ± SEM of 7 experiments for WT, and 2 experiments for *kin-29* mutants. The second y-axis shows the averaged fraction of nonpumping animals (*n* = 10) for each genotype and time point. Statistical comparisons were performed with a 2-way ANOVA using time and genotype as factors, followed by post hoc pairwise comparisons at each time point to obtain nominal *p*-values, which were subjected to a Bonferroni correction for multiple comparisons. ***, **, and * indicate corrected *p*-values that are different from WT at *p* < 0.001, *p* < 0.01, and *p* < 0.05, respectively (S1 Data, Sheet 3F). (G) L1 time course of total body p-AMPK divided by actin loading control in WT and *kin-29* null mutant animals. Data are normalized to the average value of the WT time-course period. Graphs show the mean ± SEM of 3 experiments for WT (8 replicates) and *kin-29* mutants (5 replicates). The second y-axis shows the averaged fraction of nonpumping animals (*n* = 10) for each genotype and time point. Statistical comparisons were performed by an unpaired multiple-comparison *t* test with Holm-Sidak correction. ***, **, and * indicate corrected *p*-values that are different from *kin-29* mutants at *p* < 0.001, *p* < 0.01, and *p* < 0.05, respectively. Representative western blots are shown adjacent to the graph (S1 Data, Sheet 3G). (H) Total body ATP levels per μg protein at maximum quiescence (2 hr) after UVC exposure in WT animals and in *kin-29* null mutants. Although levels before irradiation are lower in *kin-29* mutants, they are not significantly different 2 hr after UVC irradiation. Data are normalized to WT without UVC irradiation (0 hr). Graphs show the mean ± SEM of 4–6 experiments. **p < 0.01. Statistical comparisons were performed with a mixed-effects analysis using time and genotype as factors, followed by post hoc pairwise comparisons at each time point to obtain nominal *p*-values, which were subjected to a Bonferroni correction for multiple comparisons (S1 Data, Sheet 3H). (I) Total body p-AMPK divided by actin loading control at 2 hr after UVC exposure in WT and in *kin-29* null mutants. Graphs show the mean ± SEM of 3 experiments. **p < 0.01. Statistical comparisons were performed with a 2-way ANOVA using time and genotype as factors, followed by post hoc pairwise comparisons at each time point to obtain nominal *p*-values, which were subjected to a Bonferroni correction for multiple comparisons. A representative western blot is shown adjacent to the graph (S1 Data, Sheet 3I). DTS, developmentally timed sleep; L1; first larval stage; L4, fourth larval stage; ns, not significant; p-AMPK, phosphorylated adenosine monophosphate regulated protein kinase; SIS, stress-induced sleep; UVC, ultraviolet C; WT, wild type.

We assessed sleep phenotypes of *kin-29* null mutants. As previously reported [27], *kin-29* mutants had reduced lethargus/DTS (Fig 3A and 3B; S3B and S3C Fig). In addition, *kin-29* mutants had reduced SIS as determined by movement and feeding quiescence of animals exposed to UV radiation (Fig 3C and 3D; S3D Fig) or to heat stress (S3E and S3F Fig). Together with prior observations of quiescence defects in the setting of satiety [31], these data suggest that *kin-29* is generally required for sleep.

Based on our above analysis indicating that ATP levels’ changes inversely correlate with sleep drive, there are at least 2 possibilities for the reduced sleep of *kin-29* mutants. First, it is possible that cellular energy levels are high in *kin-29* mutants, thereby reducing sleep drive. Alternatively, it is possible that *kin-29* mutants have low ATP levels but are defective in the sleep response to low ATP levels. We found that both ATP and p-AMPK levels were lower in *kin-29* mutants than in wild-type controls during the L4 stage (Fig 3E and S3H Fig). Consistent with our whole animal tissue extract ATP determinations, a validated luminescence assay for in vivo ATP levels [54] showed reduced ATP levels in *kin-29* adult animals (S3G Fig).

Animals with reduced cellular energy, due to either reduced food intake (e.g., *eat-2* mutants [55–57]) or reduced ability to liberate energy from food [58], forage hyperactively in the presence of ample food and leave the bacterial lawn frequently [59,60]. As predicted by our measurements of low ATP levels, *kin-29* mutants left the bacterial lawn more frequently than wild-type animals (S4A Fig). Therefore, both biochemically and behaviorally, *kin-29* mutants show evidence of low cellular ATP levels.

These results suggested that in *kin-29* mutants, ATP production is reduced, ATP consumption is increased, or both. We examined the total ATP and p-AMPK levels before, during, and after lethargus/DTS. In contrast to the dynamic ATP levels during larval development observed in wild-type animals (Fig 1A), both the ATP and p-AMPK levels remained constant and low in *kin-29* mutants across DTS/lethargus (Fig 3F and 3G) and in SIS (Fig 3H and 3I; S3I Fig). These results suggest that KIN-29 is required to generate normal cellular energy levels, which may be required to promote sleep.

ATP is generated by breakdown of macromolecules such as triglycerides [61]. During cultivation of the animals, we noted that *kin-29* mutants had darker intestines than wild-type animals when viewed under bright-field stereomicroscopy. An optically dense intestinal phenotype correlates with elevated fat stores [62–64]. We therefore hypothesized that *kin-29* mutants had increased fat stores.

**Fig 4:**
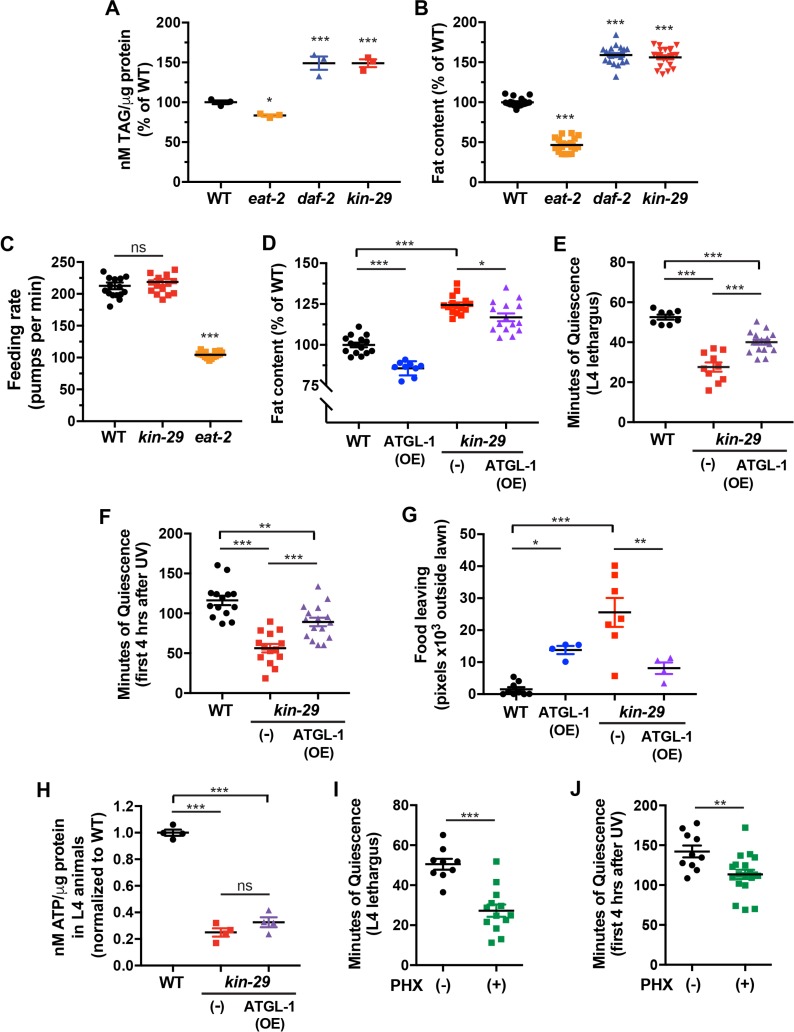
*kin-29* mutants have increased total body fat and food-leaving behavior, which are partially suppressed by ATGL-1 OE. (A) Total triglyceride (TAG) levels per μg protein. The temperature-sensitive *daf-2* insulin receptor mutant was raised at 15°C (permissive) and shifted to 20°C (restrictive) at the early L4 stage before TAG measurements during the adult stage. Data are represented as the percentage of total TAG in WT controls ± SEM of 3 experiments. * and *** indicate values that are different from WT at *p* < 0.05 and *p* < 0.001, respectively, by an ANOVA with Tukey multiple-comparisons test (S1 Data, Sheet 4A). (B) Fat content measured with fixative Oil Red O staining for each indicated genotype. Data are represented as a percentage of body fat in WT controls ± SEM (*n* = 19–21 animals). *** indicates values that are different from WT at *p* < 0.001 by an ANOVA with Tukey multiple-comparisons test (S1 Data, Sheet 4B). (C) The feeding rate of *kin-29* null mutants is not different from that of WT control animals. Feeding rate was measured as the number of feeding motions (pumps per minute) in the presence of food for each indicated genotype with *eat-2* feeding-defective mutants showing as expected reduced feeding rate. Mean ± SEM (*n* = 16 animals). *** indicates values that are different from WT at *p* < 0.001 by an ANOVA with Tukey multiple-comparisons test (S1 Data, Sheet 4C). (D) ATGL-1 OE reduces the increased fat stores of *kin-29* null mutants. Fat content was measured with fixative Oil Red O staining. Data are represented as a percentage of body fat in WT control animals ± SEM (*n* = 9–16 animals per group). ATGL-1 (OE) indicates OE of ATGL-1. *p < 0.05 and ***p < 0.001 by an ANOVA with Tukey multiple-comparisons test (S1 Data, Sheet 4D). (E and F) ATGL-1 OE partially restores the reduced L4 lethargus/DTS (E) and UVC-induced quiescence (F) of *kin-29* null mutants. Data are represented as the mean ± SEM with *n* = 8–17 animals for DTS and *n* = 14–16 animals for SIS. ***p < 0.001 and **p < 0.01 by an ANOVA with Tukey multiple-comparisons test (S1 Data, Sheet 4E and 4F). (G) ATGL-1 OE reduces the increased food-leaving behavior of *kin-29* null mutants. Food leaving was measured as the area of exploration with each data point representing tracks from a population outside the bacterial lawn. Each data point represents the total number of pixels outside of the bacterial lawn of 5 animals per plate, and the horizontal line represents the mean ± SEM of individual experiments (S1 Data, Sheet 4G). Representative images are shown of food-leaving behavior, in which frames from a 12-hr video were collapsed into a single image. ***p < 0.001 and **p < 0.01 by an ANOVA with Tukey multiple-comparisons test. (H) The reduced ATP levels per μg protein in L4 larvae of *kin-29* null mutants are not significantly restored by ATGL-1 OE. Data are normalized to WT and represented as the mean ± SEM of 4 experiments. ***p < 0.001 by an ANOVA with Tukey multiple-comparisons test (S1 Data, Sheet 4H). (I and J) PHX treatment results in reduced body movement quiescence during L4 lethargus/DTS (I) and after UVC exposure/SIS (J) in WT animals. Total minutes of body movement quiescence during L4 lethargus, and during the first 4 hr after UVC exposure in adults in the presence (+) of 1 mM PHX or in the absence (-) of PHX. Shown is 1 representative biological replicate of an experiment performed 4 times. Data are represented as mean ± SEM with *n* = 9–13 animals for DTS and *n* = 10–19 animals for SIS. ***p < 0.001, **p < 0.0 by an unpaired 2-tailed *t* test (S1 Data, Sheet 4I and 4J). ATGL-1, adipose triglyceride lipase-1; DTS, developmentally timed sleep; L4 stage, fourth larval stage; ns, not significant; OE, overexpression; PHX, perhexiline; SIS, stress-induced sleep; TAG, triacylglyceride; UVC, ultraviolet C; WT, wild type.

To test this hypothesis, we measured fat levels in *kin-29* null mutants using multiple methods including fixative Oil Red O, fixative Nile Red staining [65], and measurement of triacylglycerides (TAGs) in worm extracts. Using these fat-ascertainment methods, we observed increased fat stores in *kin-29* mutants (Fig 4A and 4B). The *kin-29* increased-fat phenotype was present throughout the animal life span from the L1 stage through the adult stage (S4B Fig). As controls for our fat-ascertainment methods, we observed increased fat in animals mutant for the insulin receptor DAF-2 [63] and decreased fat in animals mutant for the gene *eat-2* [64,66], which is required for food intake [55,67] (Fig 4A and 4B). To further characterize the excess fat phenotype of *kin-29* mutants, we used a green fluorescent protein (*gfp*) reporter that marks the surface of lipid droplets (DHS-3::GFP) [68]. *kin-29* mutants had increased number and size of lipid droplets in comparison with wild-type animals (S4C Fig).

The fat phenotype of *kin-29* mutants is not explained by increased food intake, because feeding behavior as measured by the frequency of pharyngeal contractions (Fig 4C) and by the uptake of fluorescence microspheres (S4D Fig) [69] was not elevated in *kin-29* mutants when compared with well-fed control wild-type animals.

In summary, these fat-assessment methods all show elevated fat stores despite normal food intake and reduced ATP levels in *kin-29* mutants.

### Sleep defects of KIN-29 mutants are corrected by liberation of energy stores

One explanation for the mutant sleep and metabolic phenotypes is that *kin-29* is required to respond to dropping cellular ATP levels by promoting in parallel both sleep and the liberation of energy from fat stores. An alternative explanation is that sleep is required for fat mobilization and that in the absence of sleep (as seen in *kin-29* mutants), fat is no longer mobilized properly during lethargus. Finally, a third explanation is that fat breakdown is the signal for sleep and that *kin-29* mutants do not liberate fat energy stores when ATP levels drop. If this third, linear explanation in which *kin-29*→fat mobilization→sleep, is correct, then (1) reduction of sleep by other means should not affect fat mobilization or ATP levels during lethargus; and (2) it should be possible to bypass the need for *kin-29* in promoting sleep by using a genetic manipulation that liberates fat directly.

To test whether sleep is required for fat mobilization and cellular energetics, we measured fat stores and ATP levels in *aptf-1* mutants, which display minimal movement quiescence due to a defective RIS neuron [42]. Fat stores and ATP levels in *aptf-1* mutants were no different from wild-type controls (S5A and S5B Fig), suggesting that movement quiescence is not required for fat mobilization. However, since worms do not feed during lethargus, even when mutant for *aptf-1* [42], it remains possible that specifically feeding quiescence is required for fat mobilization.

We next tested whether we can bypass the need for *kin-29* in sleep promotion by experimentally liberating fat in a *kin-29* mutant. The adipose triglyceride lipase-1 (*atgl-1*) encodes the *C*. *elegans* orthologue of the rate-limiting enzyme in mammalian fat breakdown [70,71] and is expressed in the *C*. *elegans* intestinal cells that store fat [72]. We overexpressed ATGL-1 and assessed both cellular energy stores and sleep behavior. We observed a reduction of body fat stores in *kin-29* mutants (Fig 4D and S6C Fig), indicating that the ATGL-1 overexpression achieved the intended goal. ATGL-1 partially restored the defective DTS and SIS phenotype of *kin-29* mutants (Fig 4E and 4F; S6A and S6B Fig) and corrected the food-leaving phenotype (Fig 4G and S6D Fig) but did not cause a measurable increase in ATP levels (Fig 4H). These results suggest that it is the liberation of fat from intestinal cells by ATGL-1 overexpression and not the increase in ATP levels that promotes sleep and reduced food leaving. However, we cannot exclude the possibility that our ATP assay was not sufficiently sensitive to detect an ATP increase in response to ATGL-1 overexpression. Based on our data (Fig 4H), we estimate that we would require an *N* = 16 to have 80% power to detect a difference (at *p* < 0.05) if in fact there were one. In contrast to its effects on behavior of *kin-29* mutants, ATGL-1 overexpression in a wild-type background did not significantly affect DTS or SIS (S6E and S6F Fig), suggesting that fat liberation is already maximal in wild-type animals.

We hypothesized that the mechanism by which ATGL-1 overexpression promotes sleep is via beta-oxidation of the liberated fatty acids. To test this hypothesis, we used the carnitine palmitoyltransferase (CPT) inhibitor perhexiline (PHX) to block fatty acid oxidation [73] (S7A Fig). We found that PHX treatment impaired body movement quiescence during lethargus/DTS (Fig 4I and S7B Fig) as well as during SIS (Fig 4J and S7C Fig). In addition, PHX had a small but significant suppression of feeding quiescence during SIS (S7D Fig). The fraction of feeding quiescent animals after ultraviolet C (UVC) exposure was 59.3% ± 1.4% (mean ± SEM, *n* = 27) in the presence of vehicle and 28.6% ± 1.8% (mean ± SEM, *n* = 28) in the presence of PHX.

Taken together, these results indicate that KIN-29 responds to dropping ATP levels to signal the intestinal cells to liberate and metabolize fatty acids, which then results in signals to sleep-promoting centers by yet unclear mechanisms.

### A sensory neuron basis for KIN-29 SIK in the metabolic regulation of sleep

**Fig 5:**
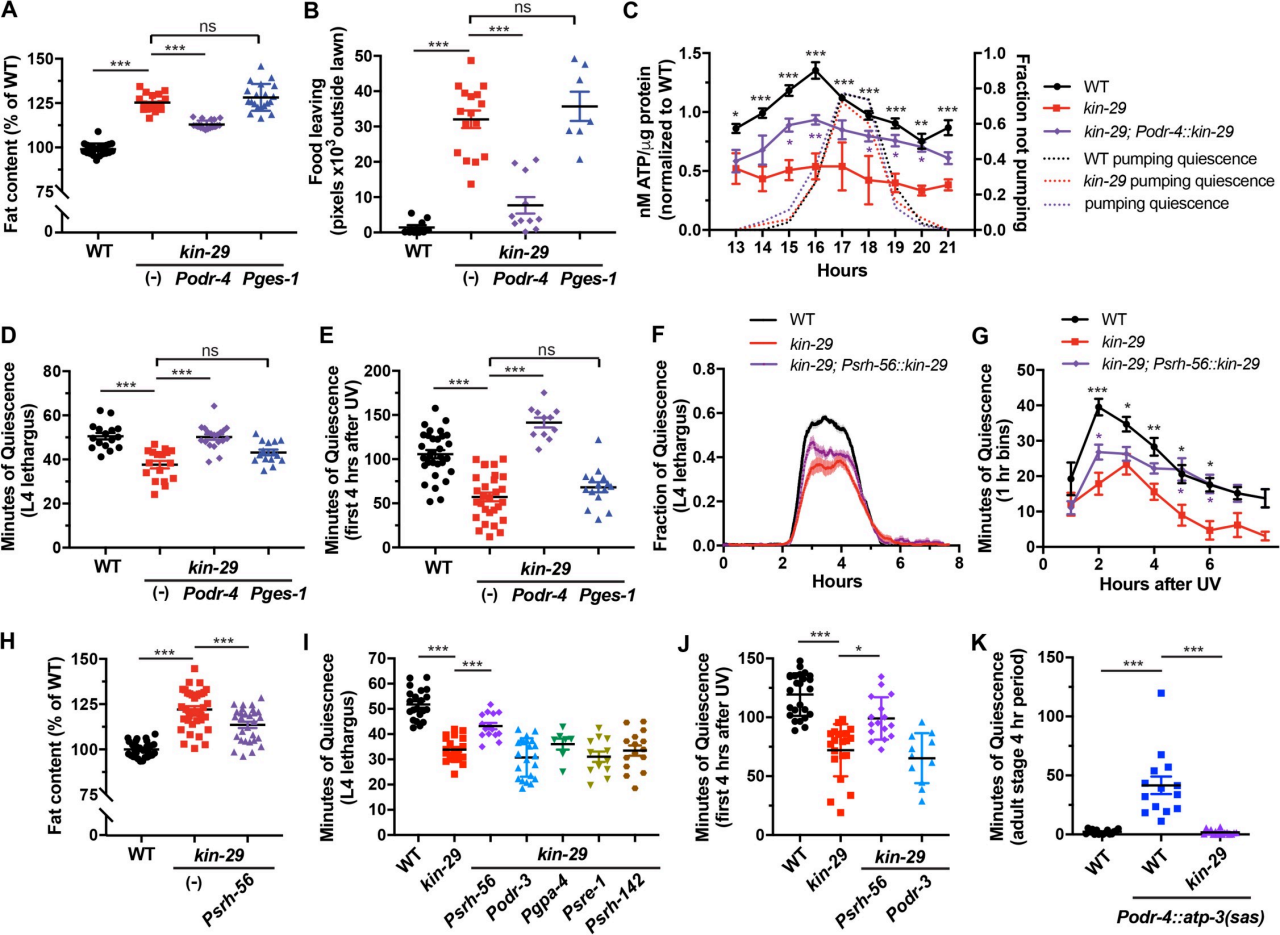
*kin-29* acts in a subset of sensory neurons to regulate DTS and SIS sleep, fat stores, and food-leaving behavior. (A and B) Expression of *kin-29* in sensory neurons but not in the gut corrects the increased fat content (A) and increased food-leaving behavioral (B) phenotypes of *kin-29* null mutants. *odr-4* is expressed in 12 pairs of sensory neurons. *ges-1* is expressed in the intestine. Fat content was measured with fixative Nile Red staining, and data are represented as a percentage of total body fat in WT controls ± SEM (*n* = 18–24 animals). Food leaving was quantified as the area of exploration with each data point representing tracks from a population outside the bacterial lawn. Each data point represents the total number of pixels outside of the bacterial lawn of 7 animals per plate, and the horizontal lines represent the mean ± SEM of individual experiments. *** indicates values that are different from WT and nontransgenic (-) *kin-29* animals at *p* < 0.001 by an ANOVA with Tukey multiple-comparisons test (S1 Data, Sheet 5A and 5B). (C) Total body ATP per μg protein measured before, during, and after L1 lethargus/DTS of the indicated genotypes. *kin-29* animals expressing *Podr-4*::*kin-29* partially restore the reduced ATP levels of *kin-29* mutants. *Podr-4*::*kin-29* is an extrachromosomal transgenic array, and about 20% of siblings of this strain that have lost the array (and are therefore *kin-29* mutants) are included in the ATP measurements, thereby reducing the overall ATP levels. Data are normalized to the average value of the WT time course. The second y-axis shows the averaged fraction of nonpumping animals (*n* = 10) for each genotype and time point. Graphs show the mean ± SEM of 7 experiments for WT, 2 experiments for *kin-29* mutants, and 3 experiments for *Podr-4*::*kin-29*. Statistical comparisons were performed with a 2-way ANOVA using time and genotype as factors, followed by post hoc pairwise comparisons at each time point to obtain nominal *p*-values, which were subjected to a Bonferroni correction for multiple comparisons. ** and * indicate corrected *p*-values that are significantly different between *kin-29* mutants and *kin-29* animals carrying the *Podr-4*::*kin-29* transgene at *p* < 0.01 and *p* < 0.05, respectively (S1 Data, Sheet 5C). (D and E) The reduced body movement quiescence of *kin-29* null mutants during L4 lethargus/DTS (D) and after UV exposure/SIS (E) is restored by expressing *kin-29* in *odr-4*(+) sensory neurons, but not in the *ges-1*(+) intestinal cells (*n* > 15 animals per group). Nontransgenic (-) *kin-29*-mutant animals are shown. Data are represented as the mean ± SEM. *** indicates values that are different from WT and nontransgenic (-) *kin-29* animals at *p* < 0.001 by an ANOVA with Tukey multiple-comparisons test (S1 Data, Sheet 5D and 5E). (F and G) Reduced movement quiescence of *kin-29* null mutants during L4 lethargus/DTS (F) and after UV exposure/SIS (G) is partially restored by expressing *kin-29* in *srh-56*-expressed neurons (ASH, ASJ, ASK). (F) The fraction of quiescence in a 10-min moving window (*n* = 9–11 animals for each trace). The x-axis represents hours from the start of recording in the late L4 stage. The data from individual worms were aligned such that the start of lethargus quiescence occurred simultaneously. Shading indicates SEM. (G) Mean ± SEM of body movement quiescence per hour after UVC irradiation (1,500 J/m^2^) (*n* = 8–10 animals for each genotype). Statistical comparisons were performed by an unpaired multiple-comparison *t* test with Holm-Sidak correction. ***, **, and * indicate corrected *p*-values that are different from *kin-29* mutants at *p* < 0.001, *p* < 0.01, and *p* < 0.05, respectively (S1 Data, Sheet 5F and 5G). (H) Fat content measured with fixative Oil Red O staining. Data are represented as a percentage of total body fat in WT controls ± SEM (*n* = 28–36 animals for each genotype). *** and * indicate values that are different from WT and nontransgenic (-) *kin-29* animals at *p* < 0.001 by an ANOVA with Tukey multiple-comparisons test (S1 Data, Sheet 5H). (I and J) Minutes of body movement quiescence during L4 lethargus/DTS (I) and after UVC exposure (J) in WT animals, *kin-29* null mutant animals, and *kin-29* mutants carrying transgenes encoding *kin-29* under the control of the indicated promoters (*n* > 7 animals for each genotype). Data are represented as the mean ± SEM. *** and * indicate corrected *p*-values at *p* < 0.001 and *p* < 0.05, respectively, by an ANOVA with Tukey multiple-comparisons test (I), and Kruskal-Wallis with Dunn multiple-comparisons test (J) (S1 Data, Sheet 5I and 5J). (K) Minutes of body movement quiescence of *Podr-4*::*atp-3(sas*) adult transgenic animals during 4 hr (*n* = 14–16 for each genotype). Data are represented as the mean ± SEM. *** indicate corrected *p*-values that are different from WT or *kin-29* mutants at *p* < 0.001 by a Kruskal-Wallis with Dunn multiple-comparisons test (S1 Data, Sheet 5K). DTS, developmentally timed sleep; *ges-1*, gut esterase 1; L1, first larval stage; L4 stage, fourth larval stage; ns, not significant; SIS, stress-induced sleep; UVC, ultraviolet C; WT, wild type.

Similar to broad expression of *sik* genes in mammals [28], *kin-29* is broadly expressed in both neural and nonneural cells in *C*. *elegans* [29]. Since fat is stored primarily in *C*. *elegans* intestinal cells [36], we initially asked whether the excessive fat phenotype of *kin-29* mutants is explained by intestinal action of KIN-29. Expression of *kin-29* under the intestine-specific gut esterase 1 (*ges-1*) promoter did not rescue the excess fat, the food-leaving behavior, or sleep defects of *kin-29* mutants (Fig 5A and 5D; S8A and S8B Fig), indicating that *kin-29* does not act in the gut to regulate these phenotypes.

We next assessed a role for *kin-29* in the nervous system. The sensory nervous system of *C*. *elegans*, similar to the mammalian hypothalamus, plays an important role in sensing nutrient availability and signaling to regulate animal metabolism [74]. Two *kin-29* phenotypes, a small body size and the propensity to enter the dauer diapause stage, are corrected by using the *odr-4* promoter to express the *kin-29* cDNA in 12 pairs of sensory neurons [29]. We tested the hypothesis that the fat-storage phenotype too is controlled by *kin-29* acting in these *odr-4*(+) sensory neurons. *odr-4* promoter–driven *kin-29* rescued the high-fat stores (Fig 5A and S8A Fig), food-leaving starvation behavior (Fig 5B and S8A Fig), lipid droplet morphology (S4C Fig), and low-ATP-level phenotypes (Fig 5C and S8C Fig) of *kin-29* mutants. In addition, *Podr-4*::*kin-29*, but not *Pges-1*::*kin-29*, rescued the defective DTS and SIS phenotypes of *kin-29* mutants (Fig 5D and 5E; S8B Fig).

We next examined the role of KIN-29 function in DTS and SIS in subsets of the 12 sensory neurons defined by the *odr-4* promoter activity (S9A Fig). Reconstitution of *kin-29* function in the ASH, ASK, and ASJ sensory neuron pairs using the *srh-56* promoter (S9B Fig) partially corrected the DTS and SIS phenotype (Fig 5F and 5G) as well as the fat phenotypes (Fig 5H and S9C Fig) of *kin-29* mutants. In contrast, *kin-29* expressed in single or subsets of sensory neurons under the control of 4 different other promoters (i.e., *odr-3*, *gpa-4*, *sre-1*, and *srh-142*) did not rescue the sleep phenotypes (Fig 5I and 5J; S9D and S9E Fig). These data suggest that KIN-29 function in ASK and/or ASJ is important for sleep and lipid homeostasis. Because the rescue of these phenotypes using the *srh-56* promoter to drive *kin-29* expression is weaker than the rescue using the *odr-4* promoter, *kin-29* likely also functions in other, as yet undefined, *odr-4*(+) neurons to regulate sleep and lipid homeostasis. However, we cannot rule out the possibility that these differences are explained by different strengths of the promoters used.

The ATP synthase of the mitochondrial energy chain is a component of the primary cellular energy–producing machinery [75]. To examine the effects of ATP depletion on sleep, we knocked down the gene *atp-3*, which encodes a subunit of the mitochondrial ATP synthase. We performed the knockdown by expressing sense and antisense (sas) *atp-3* RNA under control of the *odr-4* promoter [76] and measured quiescence in adults. Similar to *kin-29* mutants [29] and other sensory neuron mutants [77], *atp-3(sas)* transgenic animals were small (S10B Fig), suggesting that the transgene effectively impaired sensory neuron function. However, it is unlikely that *atp-3* knockdown resulted in death of *odr-4*(+) neurons, since morphology of these neurons was grossly intact (S10C Fig).

Adult animals carrying *atp-3(sas)* in *odr-4*(+) neurons showed increased movement quiescence (Fig 5K and S10A Fig). To test for tissue specificity of the knockdown, we also made transgenic animals in which *atp-3(sas)* was expressed under control of the intestinal *ges-1* promoter. Effective knockdown of *atp-3* function in the intestine was supported by observing reduced ATP levels in *Pges-1*::*atp-3(sas)* animals (S10D Fig). However, knocking down *atp-3* in the intestine did not promote movement quiescence in adults (S10E Fig). These results suggest that reducing the rate of ATP production specifically in sensory neurons but not the gut promotes sleep. To test whether *kin-29* is required for the increased quiescence of *atp-3(sas)* transgenic animals, we crossed these transgenics into animals mutant for *kin-29*. *kin-29* mutants fully suppressed the increased quiescence of *Podr-4*::*atp-3(sas)* animals (Fig 5K). We also examined *isp-1*, a mitochondrial function mutant with low ATP levels [78]. *isp-1* mutants were highly quiescent following a 20-min heat shock at 35°C, and this quiescence largely depended on *kin-29* function (S10F Fig).

Taken together, these data further support a role for *kin-29* acting in sensory neurons that respond to low ATP levels to regulate intestinal fat and organismal sleep. Importantly, our data suggest that *kin-29* acts in the same neurons to regulate both fat and sleep, as would be predicted by a linear model in which fat liberation promotes sleep.

### KIN-29 SIK acts upstream of ALA and RIS activation to promote sleep

The *odr-4* gene is not expressed [79] in the 2 best-characterized interneurons regulating sleep, the ALA [39] and RIS [42] neurons, suggesting that *kin-29* does not act in these sleep-executing neurons but rather acts at a step either before or after activation of these neurons.

**Fig 6:**
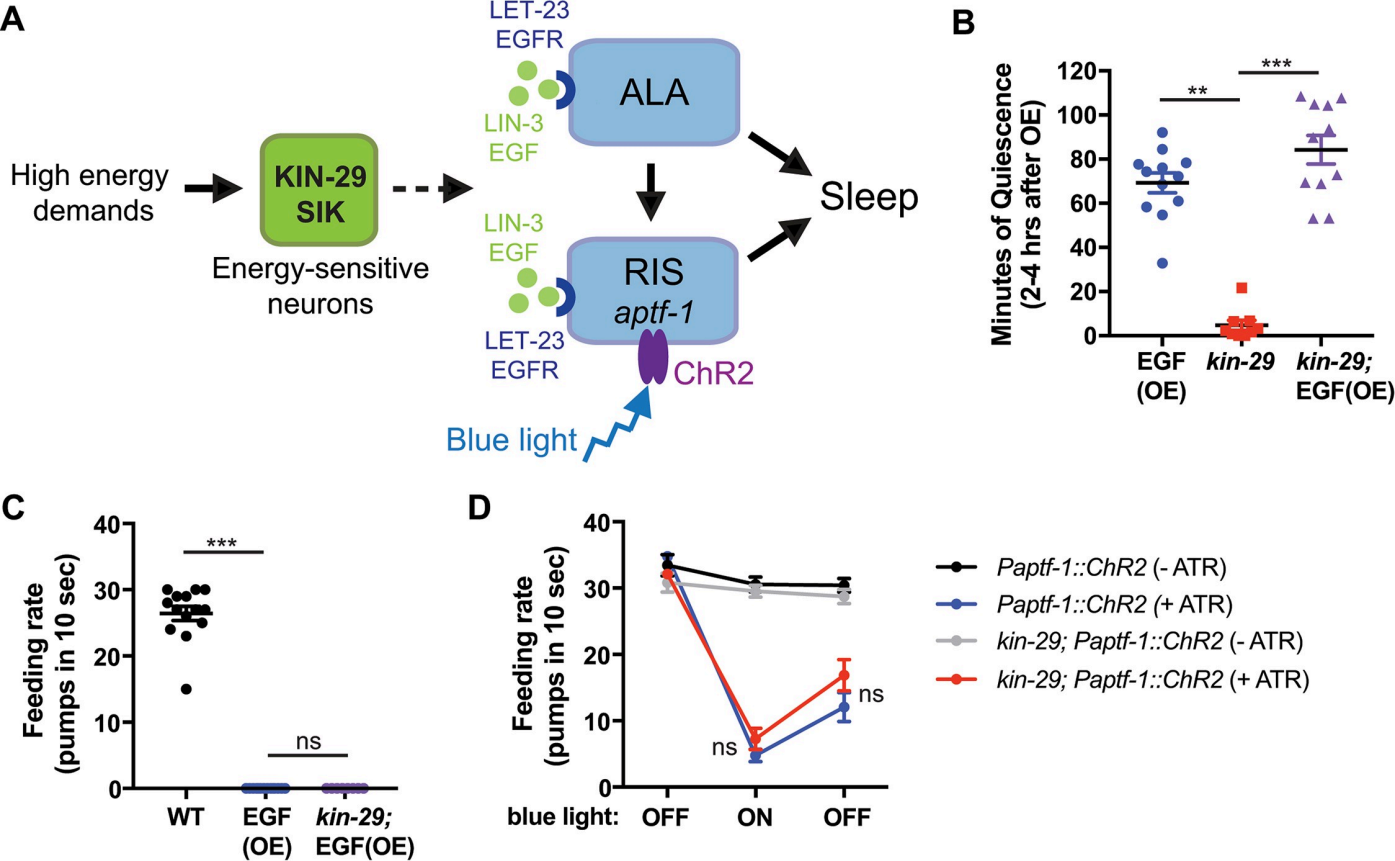
*kin-29* acts upstream of ALA and RIS neurons to regulate sleep. (A) Model by which *kin-29*/SIK functions in energy-sensitive sensory neurons upstream of the sleep-promoting RIS and ALA neurons. ALA and RIS activation by LIN-3/EGF or RIS activation by ChR2 bypasses the requirement for KIN-29 function in sleep. RIS is also required for movement quiescence during SIS (see S11C–S11F Fig and [43]). (B and C) *kin-29* null mutation does not affect the body movement quiescence (B) and reduced feeding rate (C) observed in response to EGF OE (*n* > 10 animals). To induce EGF OE, adult animals expressing a *Phsp-16*.*2*::*LIN-3C* transgene were heat-shocked for 30 min, 2 hr prior to analysis of behavior (see Material and methods). Data are represented as mean ± SEM. *** and ** indicate corrected *p*-values that are different from WT or *kin-29* mutants at *p* < 0.001 and *p* < 0.01, respectively, by a Kruskal-Wallis with Dunn multiple-comparisons test (S1 Data, Sheet 6B and 6C). (D) Optogenetic stimulation of the RIS neuron causes a reduction in feeding rate, which is not dependent on *kin-29*. WT and *kin-29* null mutants expressing *Paptf-1*::*ChR2* grown either in the presence or absence of ATR were exposed to blue light (ON) (see Material and methods). Pumps were counted during a 10-s window, before, during, and after exposure of transgenic animals (*n* = 9–13) to blue light. Data are represented as the mean ± SEM for each condition. ns indicates values that are not different between WT *Paptf-1*::*ChR2* (+ATR) and *kin-29*; *Paptf-1*::*ChR2* (+ATR) by an ANOVA with Tukey multiple-comparisons test S1 Data, Sheet 6D). ATR, all-*trans* retinal; ChR2, channelrhodopsin2; EGF, epidermal growth factor; EGFR, EGF receptor; ns, not significant; OE, overexpression; SIK, salt-inducible kinase; SIS, sleep-induced stress; WT, wild type.

Epidermal growth factor (EGF) activates the ALA [80] and RIS [81] neurons, which regulates SIS by releasing a cocktail of neuropeptides including those encoded by the gene *flp-13* [45,82]. To determine whether *kin-29* functions upstream or downstream of EGF signaling (Fig 6A), we asked whether the quiescence-inducing effect of EGF overexpression is attenuated in *kin-29* mutants. We observed no effect of a *kin-29* null mutation on the quiescence induced by overexpressing EGF (Fig 6B and 6C), supporting the notion that *kin-29* acts upstream or in parallel of EGF signaling. As expected for a gene acting upstream or in parallel of ALA activation, the *kin-29* null mutation also did not attenuate the quiescence induced by overexpressing *flp-13* (S11A and S11B Fig). In fact, *kin-29* mutation appeared to potentiate rather than suppress the quiescence-inducing effects of FLP-13 overexpression. This potentiation might be explained by elevated activity of heat shock–mediated gene regulation in the *kin-29* mutants.

DTS is primarily controlled by the RIS neuron, which releases neuropeptides encoded by the gene *flp-11* [83]. In addition to the requirement of ALA for SIS, we observed that RIS is required for body movement quiescence as recently reported [43,81] and, to a lesser extent, for feeding quiescence during SIS (Fig 6A; S11C–S11F Fig). To ask whether KIN-29 functions upstream of RIS, we crossed *kin-29* mutants into a strain expressing channelrhodopsin2 (ChR2) under the *aptf-1* promoter to activate RIS [42]. Illuminating adult worms expressing *Paptf-1*::*ChR2* with blue light leads to cessation of pumping when worms are treated with the ChR2 cofactor all-*trans* retinal (ATR) but no change in pumping rate in non-ATR controls [42]. The *kin-29* null mutation did not impair the ATR-dependent reduction in pumping in response to optogenetic activation of *aptf-1-*expressing neurons (Fig 6D), indicating that KIN-29 acts upstream, or in parallel, of RIS.

Together, these results indicate that KIN-29 functions in energy-sensitive sensory neurons upstream of the sleep-promoting ALA and RIS neurons. These data are again consistent with a linear model in which *kin-29*, in response to dropping ATP levels, promotes fat liberation, which in turn promotes sleep via activation of ALA and/or RIS.

### KIN-29 SIK acts in sensory neuron nuclei to regulate sleep

Under standard growth conditions, KIN-29 localizes to the cytosol, but in response to cell stress induced by high heat exposure, KIN-29 moves into the nucleus [29]. It regulates gene transcription via interaction with the nuclear factors the myocyte enhancer factor 2 (MEF-2) and the histone deacetylase 4 (HDA-4) [84]. In contrast, the mammalian KIN-29 homolog SIK3 protein has been proposed to act in the cytosol to phosphorylate synaptic proteins [30]. To determine where KIN-29 acts to regulate sleep, we began by assessing its subcellular localization during sleep.

**Fig 7:**
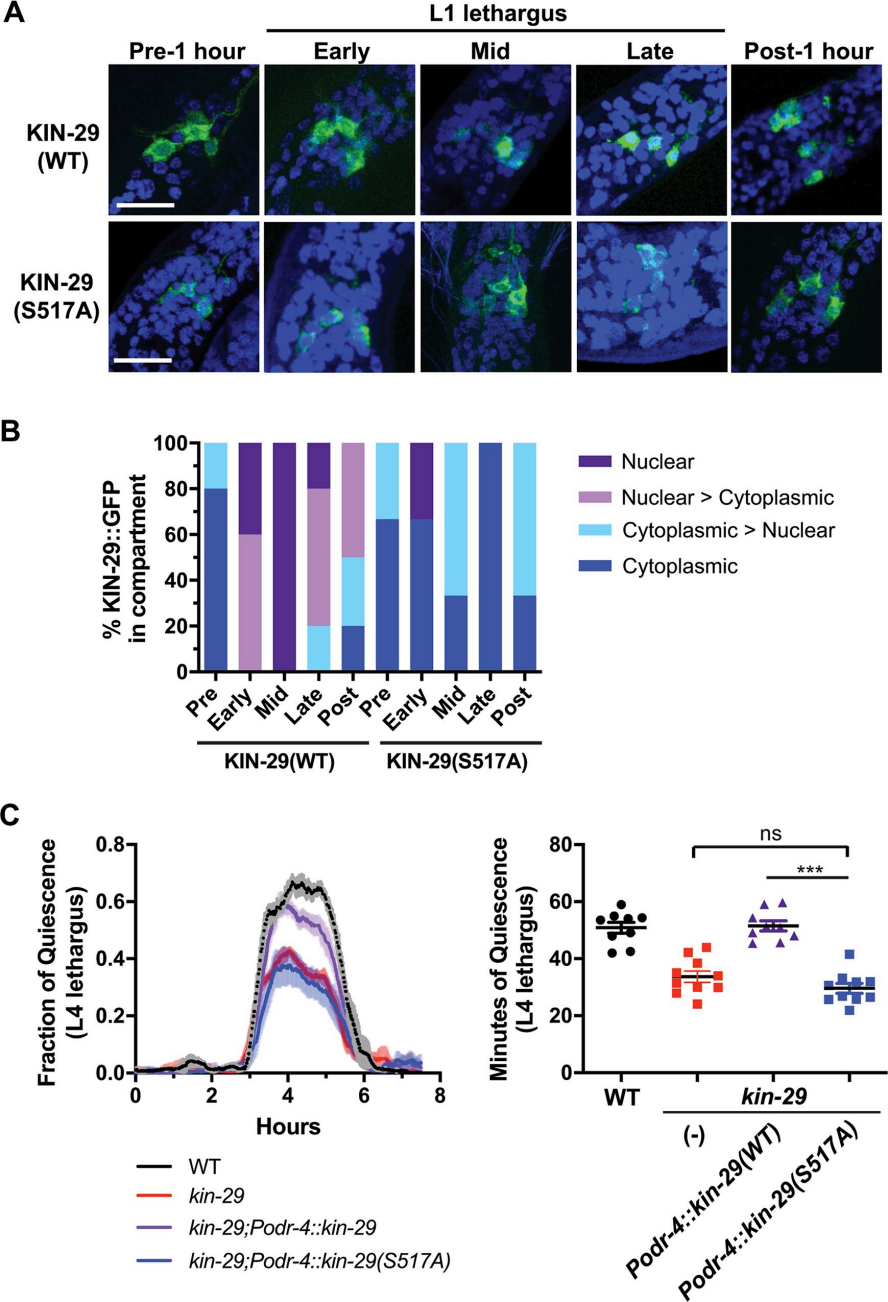
KIN-29(S517A) mutant does not translocate to the nucleus during sleep and does not rescue the short-sleeping phenotype of *kin-29*. (A) Confocal images of KIN-29::GFP in *odr-4*(+) neurons (green) of WT animals. Nuclei are identified by blue DAPI staining. One hour prior to L1 lethargus and 1 hr after L1 lethargus (*n* = 3–5 animals for each condition), KIN-29::GFP is mostly cytoplasmic. During mid and late L1 lethargus (*n* = 5–7 animals for each condition), KIN-29, but not the KIN-29(S517A) mutant, localizes to the nucleus of a subset of *odr-4*(+) neurons. Scale is 10 μm. (B) Percentage of KIN-29::GFP in the subcellular compartment of *odr-4*(+) neurons before, during, and after lethargus/DTS of the L1 stage. Shown is the percentage of animals that show fully nuclear, intermediate (nuclear > cytoplasmic, and cytoplasmic > nuclear), and fully cytoplasmic location of GFP (S1 Data, Sheet 7B). (C) The KIN-29(S517A) mutant does not rescue the reduced L4 lethargus/DTS of *kin-29* null mutants. Left graph: The fraction of quiescence in a 10-min moving window is shown with *n* = 3 animals for the WT trace, *n* = 4 animals for the *kin-29* trace, *n* = 8 animals for the *Podr-4*::*kin-29(S517A)* trace, and *n* = 6 animals for the *Podr-4*::*kin-29(WT)* trace. The x-axis represents hours from the start of recording in the late L4 stage. The data from individual worms were aligned such that the start of lethargus quiescence occurred simultaneously. Shading indicates SEM. Right graph: Total body movement quiescence during L4 lethargus/DTS determined from the time-course data. Data are represented as the mean ± SEM. ***p < 0.001 by an ANOVA with Tukey multiple-comparisons test (S1 Data, Sheet 7C). DAPI, 4,6-diamidino-2-phenylindole; DTS, developmentally timed sleep; GFP, green fluorescent protein; L1 stage, first larval stage; L4 stage; fourth larval stage; ns, not significant; WT, wild type.

One hour prior to L1 lethargus as well as 1 hr after L1 lethargus, KIN-29 expressed in *odr-4*(+) neurons was cytoplasmic (Fig 7A and 7B; S12B Fig). By contrast, during mid and late L1 lethargus, KIN-29 localized to the nuclei of a subset of *odr-4*(+) neurons (Fig 7A and 7B; S12B Fig). These data lead us to hypothesize that *kin-29* functions in the nucleus to regulate sleep. Based on this hypothesis, we would predict that a *kin-29* mutant that fails to translocate to the nucleus would have a defective regulation of sleep. We were able to test this prediction by studying the function of a KIN-29 protein with a conserved serine 517 mutated to alanine (S12A Fig). The motivation for making this particular mutant was the observation that a homologous change in the mouse SIK3 gene results in a sleepy phenotype [85]. Although we did not observe a sleepy phenotype in the *kin-29(S517A)* mutants, we found that KIN-29(S517A) mutant protein did not move to the nucleus during lethargus (Fig 7A and 7B; S12B Fig). Moreover, it did not rescue the sleep defect of *kin-29* null mutants (Fig 7C). KIN-29(S517A) stayed in the cytosol even after heat shock (S12C Fig), which strongly promotes nuclear localization of wild-type KIN-29 (KIN-29[WT]) [29]. KIN-29(S517A) was otherwise functional because it rescued the small-body-size phenotype of *kin-29* mutants (S12D Fig). Although KIN-29(S517A) protein was less abundant than KIN-29(WT), protein levels of KIN-29(WT) as well as of KIN-29(S517A) did not change during lethargus in comparison to levels before and after lethargus (S12E Fig). However, we cannot exclude the possibility that KIN-29 nuclear localization during lethargus may be affected by its overall protein levels.

**Fig 8:**
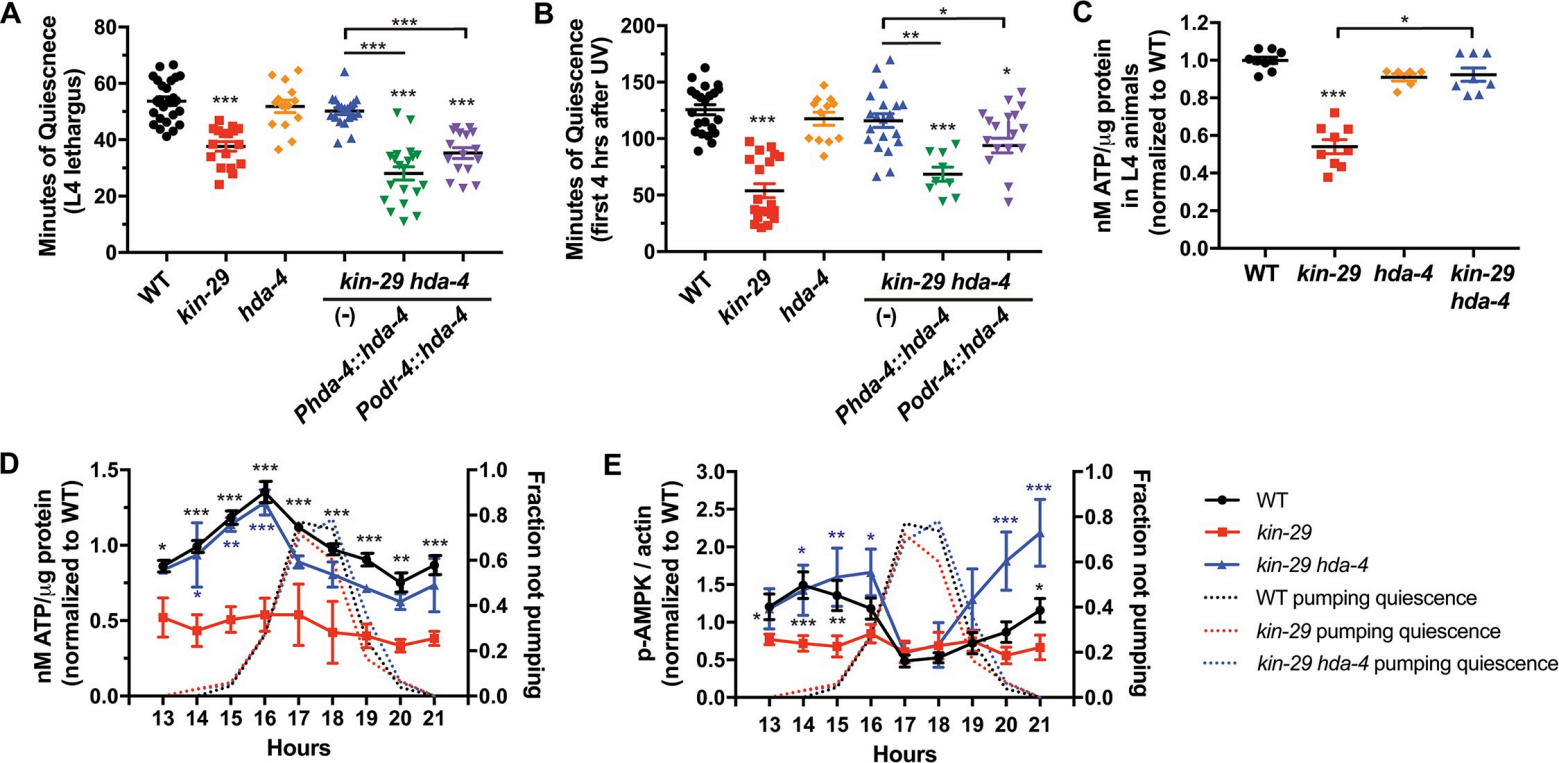
*hda-4* acts downstream of *kin-29* in sensory neurons to control the metabolic regulation of sleep. (A and B) An *hda-4* null mutation corrects the *kin-29* null mutant sleep compared with that of WT worms. Restoring *hda-4* in *kin-29 hda-4* double mutants under control of the *hda-4* promoter (*Phda-4*::*hda-4*) or the *odr-4* promoter (*Podr-4*::*hda-4*) results in reemergence of the *kin-29* quiescence defects during (A) DTS lethargus (*n* = 17–28 animals) and (B) SIS (*n* = 9–23 animals). Data are represented as the mean ± SEM. ***, **, and * indicate values that are different from WT and nontransgenic (-) *kin-29 hda-4* double-mutant animals at *p* < 0.001, *p* < 0.01, and *p* < 0.05, respectively, by an ANOVA with Tukey multiple-comparisons test (S1 Data, Sheet 8A and 8B). (C) Mutations in *hda-4* restore the reduced ATP per μg protein levels in *kin-29* mutant L4 larva. Data are normalized to WT and represented as the mean ± SEM of 6–9 experiments. ***p < 0.001, **p < 0.01 by a Kruskal-Wallis with Dunn multiple-comparisons test (S1 Data, Sheet 8C). (D) Total body ATP levels per μg protein in WT, *kin-29* single mutants, and *kin-29 hda-4* double-mutant animals measured before, during, and after L1 lethargus/DTS. Data are normalized to the average value of the WT time course. The second y-axis shows the averaged fraction of nonpumping animals (*n* = 10) for each genotype and time point. Graphs show the mean ± SEM of 2–7 experiments for ATP. Statistical comparisons were performed with a 2-way ANOVA using time and genotype as factors, followed by post hoc pairwise comparisons at each time point to obtain nominal *p*-values, which were subjected to a Bonferroni correction for multiple comparisons. ***, **, and * indicate corrected *p*-values that are different from *kin-29* mutants at *p* < 0.001, *p* < 0.01, and *p* < 0.05, respectively (S1 Data, Sheet 8D). (E) Total body p-AMPK normalized to the actin loading control in WT, *kin-29* single-, and *kin-29 hda-4* double-mutant animals measured before, during, and after L1 lethargus/DTS. Colors denoting each genotype are the same as those used in panel D. Data are normalized to the average value of the WT time course. The second y-axis shows the averaged fraction of nonpumping animals (*n* = 10) for each genotype and time point. Graphs show the mean ± SEM of 3 experiments for p-AMPK with multiple replicates for each genotype. Statistical comparisons were performed by an unpaired multiple-comparison *t* test with Holm-Sidak correction. ***, **, and * indicate corrected *p*-values that are different from *kin-29* mutants at *p* < 0.001, *p* < 0.01, and *p* < 0.05, respectively (S1 Data, Sheet 8E). AMPK, adenosine monophosphate regulated protein kinase; DTS, developmentally timed sleep; *hda-4*, histone deacetylase 4; L1, first larval stage; L4, fourth larval stage; p-AMPK, phosphorylated AMPK; SIS, stress-induced sleep; WT, wild type.

If KIN-29 were indeed acting in the nucleus to regulate sleep, then we would predict that it would genetically interact with nuclear factors. To test this prediction, we tested for genetic interactions between *kin-29* and the class II histone deacetylase HDA-4, which KIN-29 has been shown to phosphorylate and inhibit to regulate gene expression in sensory neurons [84]. HDA-4 is found in nuclei of most cells [31]. To determine whether HDA-4 is also required for the KIN-29 regulation of sleep and fat stores, we studied the phenotype of animals mutant for both *kin-29* and *hda-4*. Loss-of-function mutations in *hda-4* corrected the DTS and SIS phenotypes (Fig 8A and 8B; S13A and S13B Fig), food-leaving behavior of *kin-29* mutants (S13C Fig), and the low ATP (Fig 8C and 8D; S13D Fig) and p-AMPK abnormalities (Fig 8E; S13E and S13F Fig) of *kin-29* mutants, indicating that *hda-4* is negatively regulated by KIN-29 and acts downstream of *kin-29* to regulate sleep and starvation behavior. Expression of *hda-4* under the control of its own promoter in *kin-29 hda-4* double mutants fully restored the defective sleep phenotype of *kin-29* mutants. Expression of *hda-4* under the control of the *odr-4* promoter partially restored the defective SIS phenotype and fully restored the DTS phenotype of *kin-29* single mutants (Fig 8A and 8B; S13B Fig). These data are consistent with KIN-29 acting on HDA-4 in *odr-4*(+) sensory neurons but suggest that *hda-4* may have additional roles elsewhere in the animal.

**Fig 9:**
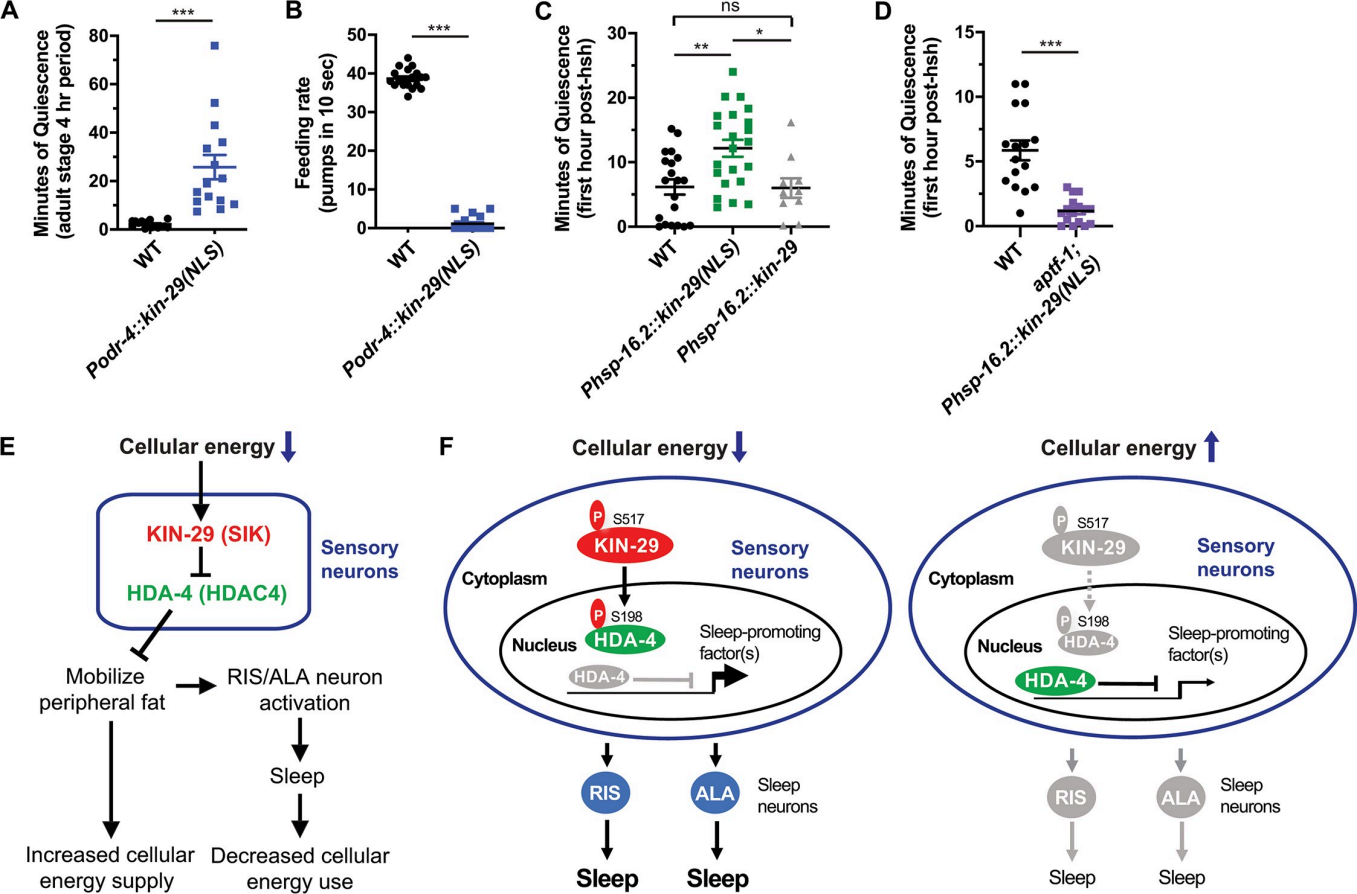
KIN-29 acts in sensory nuclei to regulate sleep. (A and B) KIN-29 expression in the nucleus of *odr-4*-expressed sensory neurons leads to anachronistic movement (A) and feeding (B) quiescence in adult animals. (A) Minutes of body movement quiescence of *Podr-4*::*kin-29(NLS)* transgenic adult animals and WT control animals during a 4-hr period. Data are represented as the mean ± SEM with *n* = 15 animals for each. ***p < 0.001 by a 2-tailed Mann-Whitney *t* test. (B) Feeding rate of *Podr-4*::*kin-29(NLS)* transgenic animals and WT control during a quiescent bout. Feeding rate was measured as pumps per 10 s. Data are represented as the mean ± SEM with *n* = 18 animals for each. ***p < 0.001 by a 2-tailed Mann-Whitney *t* test (S1 Data, Sheet 9A and 9B). (C) Nuclear KIN-29 expression under the control of an inducible heat-activated promoter *hsp-16*.*2* leads to an increased movement quiescence after hsh compared with WT and *Phsp-16*.*2*::*kin-29* controls. Minutes of body movement quiescence *Phsp-16*.*2*::*kin-29(NLS)* transgenic adult animals and WT during the first hour post hsh as determined from time-course data in S14D Fig. Adult animals were heat-shocked at 35°C for 20 min. Data are represented as the mean ± SEM with *n* = 10–22 animals for each genotype. ** and * indicate values that are different from WT and *Phsp-16*.*2*::*kin-29* transgenic animals at *p* < 0.01 and *p* < 0.05 by an ANOVA with Tukey multiple-comparisons test (S1 Data, Sheet 9C). (D) Mutations in *aptf-1* suppress the increased quiescence of *Phsp-16*.*2*::*kin-29(NLS)* transgenic animals after hsh. Minutes of body movement quiescence in *aptf-1* mutant adults expressing the *Phsp-16*.*2*::*kin-29(NLS)* transgene and WT during the first hour post hsh as determined from time-course data in S14E Fig. Adult animals were heat-shocked at 35°C for 20 min. Data are represented as the mean ± SEM with *n* = 16 animals for each genotype. ***p < 0.001 by an unpaired 2-tailed *t* test (S1 Data, Sheet 9D). (E and F) Proposed mechanism of metabolic sleep regulation. (E) KIN-29 SIK acts in response to a drop in the cellular energy charge by signaling to nonneural cells to liberate fat, which in turn promote sleep behavior. (F) Nuclear localization of KIN-29/SIK promotes sleep. Left image: When energy levels drop, KIN-29 is phosphorylated at the conserved serine position 517 and moves to the nuclei of sensory neurons that respond to cellular energy charge and phosphorylates the class II histone deacetylase HDA-4 on residue S198, thereby alleviating HDA-4-mediated repression of genes that promote sleep via RIS and ALA neurons. Right image: When KIN-29 is no longer phosphorylated at S517, it remains in the cytosol, and HDA-4 is no longer under negative regulation by KIN-29 and thus represses gene expression, thereby leading to reduced sleep. PKA may directly phosphorylate KIN-29 at the S517 residue. HDA-4, histone deacetylase 4; hsh, heat shock; NLS, nuclear localization signal; ns, not significant; PKA, Protein Kinase A; SIK, salt-inducible kinase; WT, wild type.

Another prediction made by the hypothesis that KIN-29 functions in the nucleus to promote sleep is that a transgene encoding a KIN-29 protein engineered to be predominantly in the nucleus would result in a sleepy animal. To test this prediction, we added strong nuclear localization signals (NLSs) to the C terminus of the KIN-29 protein fused to GFP (S14A Fig). KIN-29(NLS) under the control of the *odr-4* promoter was indeed localized to the nucleus of sensory neurons even outside of lethargus or stressful conditions (S14B Fig), indicating that our strategy worked. During routine cultivation of the *kin-29*(NLS) transgenic animals, we observed animals that had episodes of movement and feeding quiescence (see example, S1 and S2 Movies). We quantified the degree of quiescence and found that, although there was worm-to-worm variability (Fig 9A), *kin-29*(NLS) transgenic animals had significantly more movement and feeding quiescence than wild-type control animals (Fig 9A and 9B; S14C Fig). However, in the course of passaging this transgenic strain, the behavioral quiescence dissipated after a few generations. Since we suspected that this loss of phenotype may be explained by a selection against quiescent animals (who do not eat and lay fewer eggs), we repeated the experiment, only this time placing *kin-29*::NLS::GFP under the control of the inducible heat-activated promoter *hsp-16*.*2*. Following heat induction of transgene expression, we observed increased quiescence of animals expressing *kin-29*::NLS::GFP but not of animals expressing *kin-29*::GFP (Fig 9C and S14D Fig). This increased quiescence required RIS neuron function, since a mutation in *aptf-1* that impairs RIS function suppressed the increased quiescence of animals expressing *kin-29*::NLS::GFP (Fig 9D and S14E Fig).

Collectively, the genetic interactions between *kin-29* and *hda-4*, the subcellular distribution of KIN-29 during DTS, and the anachronistic and induced quiescence conferred by nuclear localization of KIN-29 all support the notion that KIN-29 acts in the nucleus to regulate sleep.

## Discussion

Although the focus of much of sleep function and regulation research has been on brain neurons [86], extensive observations, both basic [87] and clinical [88,89], demonstrate a role for metabolic sleep regulators outside the nervous system. Metabolic advantages of sleep include conservation of energy [15,90], proper allocation of metabolic resources [18], temporal segregation of incompatible cellular activities [91], and energetic efficiency [87]. The observation of *C*. *elegans* sleep in the setting of starvation [4,32] supports a role for sleep in energy conservation, and the observation of sleep following cell injury [39] and during lethargus [38], when nervous system activity is dampened [10–12], supports a role for sleep in the reallocation of metabolic resources from excitable cell function to anabolic and repair functions outside the nervous system. In support of an energy-conserving role for sleep, we found that preventing sleep in the adult stage results in a drop in ATP levels (Fig 1G and S2C Fig).

The absence of a significant reduction in ATP or fat levels during L1 lethargus in *aptf-1* mutants (S5 Fig) was initially surprising given the observed ATP drop during SIS caused by RIS inhibition (Fig 1G). There are at least 3 explanations of this apparent discrepancy. First, the DTS experiment was done in L1s, whereas the SIS experiment was done in adults. The number of cells is several fold smaller in the L1 than in the adult. In particular, there is no germline in the L1, so the metabolic cost of wake activity is likely lower in the L1 than in the adult stage. Second, the *atpf-1* mutation causes a chronic defect in quiescence, which may lead to compensatory changes in the animal metabolic controls. In contrast, the HisCl-based neuronal inhibition experiment causes an acute defect in behavior, for which compensatory changes would unlikely to be playing a role. Finally, we are studying 2 different types of sleep (DTS and SIS). Although there are many similarities between DTS and SIS, there are also differences [92]. One key difference is an absence of feeding during lethargus even in quiescence-defective mutants [42]. In contrast, some SIS mutants show defective feeding quiescence after UV stress in adults [44]. Since the pharynx is the largest excitable cell organ in the worm, its activity likely has a large effect on the organism’s energy stores.

Central nervous system neurons control sleep in a top-down fashion [93,94], but bottom-up metabolic signals from glia [17,24,95,96], muscle cells [32,96–98], and adipocytes [99,100] affect activity of sleep-regulating neurons. Although several gene products have been reported to regulate both metabolism and sleep [4,9,21–23,25,26,32,41,97,98,101,102], the mechanism of the metabolic regulation of sleep has heretofore remained opaque.

Our data suggest a model (Fig 9E) in which a dropping cellular energy charge of the animal is interpreted by the protein kinase KIN-29 SIK. Although numerous potential SIK3 substrates in mouse brains were recently identified [30], our genetic data suggest that KIN-29 SIK acts primarily via a single nuclear protein substrate, the type IIa histone deacetylase HDA-4, to regulate sleep. We propose that KIN-29 SIK phosphorylates and inhibits HDA-4 in the nucleus of a set of sensory neuroendocrine cells (Fig 9F). Inhibition of HDA-4 results in de-repression of genes, which in turn results in signaling from neuroendocrine cells to adipocytes to release energy stored as triglycerides. Liberated energy stores then signal to the sleep-promoting neurons ALA and RIS, which trigger organismal sleep. The mechanism of this signaling remains unknown, but one possibility is that an increase of energy stores in intestinal cells leads to the release of 1 or more of intestinal insulins, which then act on the DAF-2 insulin receptor. Supporting such a mechanism are reports that signaling by the DAF-2 insulin receptor and the forkhead box protein O (FOXO) transcription factor DAF-16 plays a role in the promotion of sleep under certain conditions [4,32,98]. An alternative possibility is that liberated free fatty acids or their metabolites play a signaling role in regulating sleep, a mechanism that would be similar to sleep regulation by arachidonic acid metabolites in mammals [103]. Finally, a third possibility is that fatty acid catabolism by-products such as reactive oxygen species promote sleep, as has recently been demonstrated in *Drosophila* [104,105].

Our finding that the roles of KIN-29 in both fat mobilization and sleep regulation map to a small number of sensory neurons supports the view that fat homeostasis and sleep are mechanistically linked. Further supporting this notion is our observation that a genetic manipulation in gut/adipocyte cells to liberate energy stored as triglycerides promotes sleep in *kin-29*-mutant animals.

Our transgenic rescue experiments implicate in the metabolic regulation of sleep by *kin-29* the sensory neuron types ASJ and ASK as well as 1 or more of 9 other sensory neuron types expressing the gene *odr-4*. Although sleep is associated with dropping ATP levels in whole animals, and *kin-29* controls sleep by action in sensory neurons, we do not yet know whether dropping energy levels are sensed specifically in these sensory neurons or elsewhere in the animal. We favor the possibility that a dropping cellular energy is detected specifically in sensory neurons, since our experimental manipulations of ATP charge in *odr-4*(+) neurons but not in intestinal cells resulted in a sleep phenotype that was *kin-29* dependent. Since information processing during wake entails a high energetic cost [106], we speculate that sensory neurons are particularly sensitive to metabolic needs of the animal, because of their position at the boundary between external and internal environment, where they can integrate more easily internal (e.g., energy levels) and external (e.g., food availability) information. By reducing their activity, sensory neurons then gate sensory information during sleep [10–12].

Loss of function of *egl-4*, which encodes a Protein Kinase G (PKG), has increased fat stores [107] and reduced sleep [38] similar to *kin-29* mutants. Like KIN-29, EGL-4 acts in sensory neurons [38] and interacts with the KIN-29 signaling pathway to regulate chemosensory receptor gene expression and other sensory behaviors [31]. EGL-4/PKG and KIN-29/SIK may regulate sleep by phosphorylating HDA-4, which would then integrate sensory and metabolic signaling.

During times of acute metabolic stress, AMPK activation plays a key role in suppressing energetically expensive anabolic processes and enhancing energy-generating catabolic processes to maintain or restore ATP intracellular levels [108]. Surprisingly, we find that the drop in ATP levels during sleep occurs without activation of AMPK by phosphorylation until after sleep. This finding suggests that turning on catabolic processes through AMPK activation may be maladaptive to the completion of the anabolic process engaged by the animal. Interestingly, in mammals, AMPK phosphorylation is also lower during sleep than during wakefulness, likely reflecting anabolic metabolism during sleep [109]. We also observed constitutively low levels of p-AMPK in *kin-29* mutants. Our observation of low p-AMPK levels in *kin-29* loss-of-function mutants is consistent with a recent observation of elevated p-AMPK levels in mice harboring a gain-of-function SIK3 mutant [30].

Though we have been unable to find an effective antibody to measure total AMPK, we believe our western blot results reflect changes in AMPK phosphorylation and not in total AMPK protein levels. Several transcriptomic analyses [110–112] did not detect a change in AMPK mRNA during lethargus, and AMPK has not been reported to be regulated at the translational or protein stability level. Nevertheless, since we have been unable to find an antibody that detects total AMPK, it remains formally possible that the variation in p-AMPK we observe is explained by a variation in total AMPK protein.

Our phenotypic characterization indicates that, like mouse and *Drosophila* SIK3, KIN-29 is required for sleep. Moreover, like *Drosophila* dSIK [33], KIN-29 is required in neurons to mobilize fat stores from adipocytes. Because KIN-29 is ancestral to all *Drosophila* and mice SIK proteins, it may alone serve functions that are served separately by dSIK and SIK3 in *Drosophila* and by SIK1, SIK2, and SIK3 in mammals.

We and others show that *kin-29*-mutant behavioral phenotypes are not restricted to sleep. *kin-29* mutants hyperforage [29], and our findings on food-leaving behavior indicate that wake behavior is different in *kin-29* mutants. Recent studies on SIK3^slp^ mice only report a sleep/wake analysis and do not report activity of these mice when awake [27,85]. Based on our findings, we predict that as in *C*. *elegans*, mice with SIK3 variants will show behavioral defects outside of sleep.

SIK3 genetic variants are associated with obesity [113,114]. It would be of interest to know whether those obese individuals also have short sleep, as would be predicted by epidemiological studies showing short sleep to be associated with obesity [3,115]. Within the framework of the linear model we propose for sleep regulation by fat, we suggest that the association between short sleep and elevated fat stores in humans could be explained by chronic obesity promoting short sleep rather than vice versa.

## Material and methods

### Strains, general animal cultivation, and genetic controls

Worms were cultivated on the surface of NGM agar. Unless otherwise specified, worms were fed the *Escherichia coli* strain OP50 [116] or its derivative DA837 [117] and grown in 20°C incubators. All experiments were performed on hermaphrodites. The wild-type strain used was N2, variety Bristol [116]. Strains used in this study are listed in S1 Table. Double-mutant animals were constructed using standard genetic methods [118], and genotypes were confirmed by genetic linkage (for example, using balancer chromosomes marked with fluorescence), by phenotype, by polymerase chain reaction (PCR) (for example, identifying small deletions), or by sequencing of a PCR product (for example, identifying single nucleotide changes).

### Generation of plasmids and transgenic animals

To generate transgenic worms expressing *kin-29* cDNA in different tissues and cells, the coding region of *kin-29* fused at its C terminus to GFP coding region and the *unc-54* 3′ UTR sequence were cloned into the multiple cloning site (MCS) of the pMC70 plasmid (a gift from the Sengupta lab), resulting in the plasmid pSL165 (*kin-29 cDNA*::*GFP*::*unc-54 3′ UTR*). Next, promoter sequences of *ges-1* (2.0 kb), *odr-4* (3.1 kb), *odr-3* (1.7 kb), *srh-56* (1.5 kb), *gpa-4* (3.0 kb), *sre-1* (1.5 kb), or *srh-142* (2.0 kb) were cloned at the 5′ end of the *kin-29* cDNA using the 5′ MCS of pSL165.

To generate transgenic animals expressing *kin-29(S517A)* cDNA under the control of the *odr-4* promoter (3.1 kb), site-directed mutagenesis (QuickChange II Site-Directed Mutagenesis Kit, Agilent, Cat # 200532) was used on the pJG40 plasmid (*Podr-4*::*kin-29 cDNA*::*GFP)* to substitute the serine at position 517 of KIN-29 to an alanine resulting in the construct *Podr-4*::*kin-29(S517A) cDNA*::*GFP*. The mutation and the absence of any amplification errors in the construct were confirmed by sequencing.

To generate a transgene encoding a KIN-29 protein with tendency to enter the nuclei of *odr-4*-expressing sensory neurons, the coding region of *kin-29* was fused at its C terminus to a SV40(NLS) tag, a GFP coding region, and an EGL-13(NLS) tag. Next, the *kin-29* cDNA::GFP fusion in the pJG40 plasmid (*Podr-4*::*kin-29 cDNA*::*GFP*) was replaced by the *kin-29* cDNA::SV40(NLS)::GFP::EGL-13(NLS) fusion, resulting in pJG66 (S14A Fig).

To generate a transgene encoding a KIN-29 protein with tendency to enter nuclei under the control of an inducible heat-shock promoter, we replaced the 3.1-kb *odr-4* promoter in pJG66 with an approximately 600-bp *hsp-16*.*2* promoter from the pPD49.78 vector with standard restriction site cloning, which resulted in *Phsp-16*.*2*::*kin-29 cDNA*::*SV40(NLS)*::*GFP*::*EGL-13(NLS)* (or pNG165). For generation of the *Phsp-16*.*2*::*kin-29*::*GFP* construct (pNG166) without the SV40(NLS) and EGL-13(NLS) tags, we inserted the same approximately 600-bp promoter sequence of *hsp-16*.*2* in the middle MCS of pJG55 containing *kin-29 cDNA*::*GFP* (S14A Fig).

To generate transgenic worms expressing HisCl in the sleep-promoting RIS neuron (*Pflp-11*::*HisCl*), the coding region of HisCl [119] as well as sequences 3′ to the gene including a splice acceptor SL2 sequence, the coding region for mCherry, and the *unc-54* 3′ UTR were amplified from the pNP471 (*Prig-3*::*HisCl*::*SL2*::*mCherry*) plasmid [119] whereas the *flp-11* promoter (1.0 kb) [83] was amplified from genomic DNA using PCR. These fragments were combined using overlap extension PCR [120], and the final PCR product was injected into N2 worms at a concentration of 50 ng/μL along with pCFJ90 (*Pmyo-2*::*mCherry*) (AddGene) at a concentration of 2 ng/μL as a transgenesis marker, and 1 kb DNA ladder (NEB) to bring the final concentration up to 150 ng/μL. Two transgenic lines were generated, NQ1208 and NQ1209 (S1 Table).

To generate a transgene encoding the *hda-4* cDNA under the control of the *odr-4* or *ges-1* promoters, the *hda-4* cDNA was fused at its C terminus to the GFP coding region and the *unc-54* 3′ UTR sequence and inserted into the middle MCS of the pMC70 plasmid (a kind gift from the Sengupta lab). Promoters of *odr-4* (3.2 kb) or of *ges-1* (2.0 kb) were then cloned at the 5′ end of the *hda-4* cDNA.

Oligonucleotides used and generated constructs are listed in S2 Table and S3 Table, respectively. Constructs were injected into N2 worms at a concentration of 20–50 ng/μl along with P*unc-122*::*RFP* (AddGene) at a concentration of 75 ng/μl as a transgenesis marker to bring the final concentration up to 125 ng/μl. Generated transgenic lines are listed in S1 Table.

### Cell-specific knockdown of *atp-3*

To knock down *atp-3* in specific cells and tissues, we used the previously described method for cell-specific RNAi knockdown [76]. Briefly, the *odr-4* (3.1 kb) or the *ges-1* (2.0 kb) promoter sequence was fused to a sense and antisense genomic sequence of the first through third exon of the *atp-3* target gene using PCR amplification with the oligonucleotides listed in S2 Table. The PCR fragments of sense and antisense expression of *atp-3* were mixed at equimolar molar amounts and injected at 50 ng/μl, together with 50 ng/μl of the transgenesis marker *Punc-122*::*RFP* (AddGene).

### Assessment of movement quiescence

Movement quiescence was measured using the 48-well (6 × 8) WorMotel [121] for SIS and a 24-well (4 × 6) WorMotel for DTS assessments. For DTS experiments, early- to mid-L4 animals were imaged for 12–18 hr. For SIS experiments, first-day-old adult worms were imaged for 8 hr after stress induction by UV or for 2 hr after stress induction by heat shock. Briefly, worms were placed individually onto the NGM agar surface of WorMotel wells together with a thin layer of bacteria. The worms were imaged under dark-field illumination provided by a red LED strip. Images were captured every 10 s for the duration of recording using approximately 8.5 μm/pixel spatial resolution. Images were analyzed by the frame subtraction method [38] using custom Matlab software (https://github.com/cfangyen/wormotel). Movement quiescence was defined as a lack of changed pixels between successive frames. Worms that left the field of view and did not return as well as worms that burrowed into the agar were censored in the analysis.

### Feeding assessment

Feeding was assessed by counting the number of movements (pumps) of the pharyngeal grinder, a toothlike structure located in the terminal bulb of the pharynx, over the course of 10 s under direct observations through a stereomicroscope with 40–115× magnification. The experimenter was blinded to the genotype/condition of the worm. One pharyngeal pump was defined as a backward movement of the grinder. Animals were considered feeding quiescent if there were no pharyngeal pumps in a 10-s window. In heat stress experiments, feeding was measured at times 0, 15, 30, 45, and 60 min after heat shock, and movement quiescence was measured continuously for 2 hr after heat shock in a separate cohort of worms. In UV stress experiments, feeding was measured 2 hr after UV exposure, and movement quiescence was measured continuously using the WorMotel device for 8 hr after UV exposure.

For the assay of microsphere accumulation in the absence of food, worms were exposed to fluorescent polystyrene microspheres of 1.0-μm diameter (Polysciences) as described [69]. In brief, a 100-μl microsphere suspension was mixed with 100 μl S-basel buffer, spread on a 3-cm NMG-agar plate, and left at room temperature for approximately 60 min for liquid absorption. An age-synchronized population was grown until the adult stage. First-day-old adult worms were washed 3 times in S-basal buffer, transferred to the microsphere plates, and incubated approximately 15 min for uptake of the microspheres. After incubation, worms were quickly washed with M9 buffer to remove excess microspheres, mounted on 2% agar pads containing the anesthetic Na-azide (NaN_3_), and imaged on a Leica DM5500 compound microscope equipped with a Hamamatsu Orca II camera. The fluorescence intensity of microspheres accumulated in the worm gut was quantified using Volocity software (PerkinElmer).

### SIS induction by UV irradiation and heat shock

UV-induced sleep assays [44] were performed by exposing first-day-old adult worms to 1,500-J/m^2^ UVC irradiation (254 nm) in a Spectrolinker XL-1500 (Spectroline). For the UV exposure, the worms were housed either in a WorMotel chip placed in an uncovered plastic 10-cm petri dish or on the agar surface of an uncovered 5.5-cm petri dish filled with NGM agar. A thin layer of *E*. *coli* DA837 or OP50 bacteria was spread onto the surface of the NGM agar immediately before the experiment. Heat-shock-induced sleep assays were performed by submerging first-day-old adult animals in a circulating water bath set to 35°C for 30 min. During the submersion, the worms were housed on the agar surface of a 5.5-cm diameter petri dish containing 11 ml NGM agar or on a WorMotel placed in an empty plastic 10-cm petri dish sealed with Parafilm.

### Induction of EGF and FLP-13 overexpression

To induce expression of LIN-3C(EGF) or FLP-13 peptides, first-day-old adult animals carrying *Phsp-16*.*2*::*lin-3C or Phsp-16*.*2*::*flp-13* transgenes were housed on the agar surface of a 5.5-cm-diameter agar surface (with 11-ml volume of NGM agar) or on a WorMotel and submerged in a circulating water bath at 33°C for 30 min. Feeding quiescence was measured 2–2.5 hr after heat-induced transgene induction, and movement quiescence was measured continuously on the WorMotel device for 8 hr after heat exposure.

### Assessment of total body fat stores

Oil Red O fixative staining was performed as described [65]. Briefly, well-fed worms were age-synchronized by the bleaching method and grown at 20°C on NGM plates seeded with *E*. *coli* OP50. L4-staged worms were collected with dH_2_O and washed over a 15-μm nylon mesh filter to remove any bacteria. Worms were transferred to a 1.5-ml tube and excess water was aspirated off. Six hundred microliters of 60% isopropanol was added to fix animals and centrifugated at 1,200 relative centrifugal force (rcf) to pellet worms. The supernatant was removed and 600 μl of Oil Red O solution was added to each tube with pelleted worms. The Oil Red O solution was made using 0.5 g Oil Red O (Sigma, Cat # O0625) in 100 ml of 100% isopropanol, filtered through a 0.20-μm PVDF filter, and allowed to equilibrate overnight with agitation at room temperature. Tubes were placed in a wet chamber and worms were stained for 6 hr at 25°C. After staining, worms were centrifugated at 1,200 rcf, washed twice, and resuspended in 0.01% Triton X-100 in S-buffer. Worms were imaged on a 2% agar pad using a Leica DMI 3000-B inverted microscope coupled to a Leica DFC295 color camera. Oil Red intensity was quantified using the Image J software (NIH). Pixel intensity was measured in the green color channel of the images. The region of the intestine measured on each animal was from the anterior part of the intestine (first cell) to region of the intestine in the mid-body at the same AP location as the vulva. Each worm was analyzed using an equivalently sized window.

Fixative Nile Red staining was performed on transgenic and nontransgenic worms as described [65]. Briefly, well-fed worms were age-synchronized by the bleaching method, and 500–1,000 L4-staged worms were washed from NGM plates seeded with *E*. *coli* OP50 using PBS containing 0.01% Triton X-100. After settling by gravity, the worms were washed once with PBS. Excess PBS was removed and 200 μl of 40% isopropanol was added to fix animals for 3 min. Next, the supernatant was removed, and 150 μl of a Nile Red (Sigma, Cat # 19123) solution in isopropanol was added to the fixed animals and allowed to stain for 30 min in the dark with agitation. After staining, worms were allowed to settle and washed once with 1× M9 buffer and kept in the dark at 4°C until visualization. Stained worms were mounted on 2% agar pads and imaged on a Leica DM5500 Nomarski microscope equipped with a Hamamatsu Orca II camera.

Triglyceride (TAG) levels were determined with a Triglyceride assay kit (Biovision, Cat # K622). Worms were age-synchronized by the bleaching method and grown at 20°C until the L4 stage on NGM plates seeded with *E*. *coli* OP50. Worms were collected and washed with S-basal solution. A 5% Triton X-100 solution with 1× protease inhibitors (Roche Complete Mini, EDTA free) was added 1:1 to a 50-μl worm pellet, and worms were sonicated with a water bath sonicator (Branson). Lipids were dissolved twice by heating the lysate to 90°C for 5 min followed by vortexing. Following centrifugation, the supernatant was used to determine the total TAG levels according to the manufacturers protocol. TAG concentrations were normalized to the total protein content as determined by a Micro BCA protein assay kit (ThermoFisher, Cat #23235). Each assay was done in triplicate and the average TAG level (nM TAG/μg protein) was calculated.

### Assessment of lipid droplet morphology

The number and size of lipid droplets was measured as reported [122] in wild-type and *kin-29* null mutants expressing the transgenic DHS-3::GFP marker. Briefly, worms were collected at the L4 stage and imaged using a Leica SP8 confocal microscope with LAS software. Images were taken with a 63× objective. The anterior 4 intestinal cells were imaged, and the diameter and number of all visible droplets in a 50-μm^2^ area were measured using Image J version 1.51h (NIH) software [123].

### Assessment of food-leaving behavior

Food-leaving behavior was video-monitored for 12 hr at 18°C by video recording 5 or 7 young adult worms housed on the agar surface of a 5.5-cm-diameter petri dish freshly seeded with 5 μl saturated OP50 suspension. The bacteria formed a circle of 0.6-cm diameter in the middle of the plate. Movies were taken on a custom-built imaging system and worm tracking software (Volumetry, version 8.a) [124] using a USB 2.0 monochrome machine vision cameras (Point Grey Research, CMLN-13SM-CS) equipped with a 12.5-mm focal length C-mount lens (Fujinon, HF12.5HA-1B). All imaging was performed under dark-field illumination using low-angle red-light LED rings as a light source, such that worms appeared as white objects against a dark background. All cameras were kept at the same height above the plates, and bacterial lawns of the same diameter were used in all experiments. Videos were recorded in uncompressed QuickTime format using StreamPix software (Norpix, Montreal, Canada) by capturing images at a rate of 0.5 frames/s. Video files were then imported into Volumetry, and each frame was converted into an 8-bit grayscale image for subsequent analysis. To quantify food-leaving behavior, we generated a binary image containing only white pixels when the grayscale value was above a user-defined threshold that approached the maximum (intensity) grayscale value (255). This binary image identified the worms in each frame. We then collapsed the resulting frames into a single image to visualize worm tracks outside of the bacterial lawn for each 12-hr video. Worm track images were imported into the ImageJ software (NIH), and the total number of pixels representing worm tracks outside of the bacterial lawn were summed.

### Measurements of ATP levels

ATP levels in whole worms were determined as described [50]. For DTS experiments, approximately 6,000–7,000 worms were age-synchronized using the double-bleaching method [125], transferred to NGM agar surface (10-cm diameter) that was fully covered with a lawn *E*. *coli* OP50, and grown at 20°C. L1 animals were washed off the agar surface using a pipette filled with 5 ml of M9 buffer. The worm and bacterial suspension was allowed to settle through a 5-μm nylon mesh filter, which passes bacteria but traps the worms. The worms trapped by the filter were then flash frozen in liquid N_2_ and stored at −80°C until analysis. Worms were collected every hour on the hour between 12 hr and 21 hr after feeding developmentally arrested L1 animals. Because at 20°C, lethargus occurs between 16.5–18.5 hr, these sampling times including animals before, during, and after L1 lethargus. For SIS experiments, 1-d-old adult worms were exposed while on an agar surface without peptone in the presence of a thin layer of bacteria to 1,500 J/m^2^ UVC irradiation, 254 nm, ultraviolet irradiation. Following irradiation, worms were transferred to new plates containing non-UV-irradiated bacteria. Worm samples were collected every hour between time 0 and 5 hr after irradiation. Thirty to 40 adults were collected in a 1.5-ml microfuge tube under stereomicroscopal observation using a platinum wire. The samples were flash frozen in liquid N_2_ and stored at −80°C until analysis. For time-course experiments, sleep was identified by measuring the fraction of nonpumping L1 worms for DTS, and the minutes per hour of body movement quiescence for SIS. For measuring ATP levels in L4 animals, worms were grown until the mid L4 stage, and 50 animals per sample were collected in a 1.5-ml microfuge tube, flash frozen in liquid N_2_, and stored at −80°C until analysis.

All samples for ATP determination were treated identically. Following collection off the agar surface using water and a nylon mesh into 1.5-ml microfuge tubes, the worms were flash frozen in liquid nitrogen within 8–12 min of preparation time. In preliminary experiments, we found that, although there was some time-dependent degradation of ATP in the first 5 min, the levels change minimally within the time window (8–12 min) of collection (S1B Fig). Samples of frozen worms were immersed in boiling water for 15 min and then placed on ice for 5 min. ATP was quantified in supernatants of worm solutions using an ATP Determination Kit (Molecular Probes, Cat #A22066) and a microplate reader (Synergy HT, Biotek) capable of luminescence measurements according to the manufacturers protocols. ATP concentrations were normalized to total protein content as determined by a Micro BCA protein assay kit (ThermoFisher, Cat #23235). ATP was measured in technical triplicates, and the average ATP concentration per μg protein was calculated per biological sample with at least 3 biological experiments for each time point unless indicated otherwise.

### Measurement of luminescence in live animals

Luminescence was measured as previously described [54]. We used a Synergy HT microplate reader (Biotek) using a 590/35-nm emission filter. Black with clear flat-bottom microplates (Corning) were used by placing about 20 worms of the strain PE254, which carry the *feIs4[Psur-5*::*luciferase*::*GFP]* transgene (PE254), in a well in 100 μl of M9 buffer. Fifty microliters of luminescence buffer (phosphate buffer [pH 6.5], 0.1 mM D-luciferin [ThermoFisher, Cat #L2916], 1% dimethyl sulfoxide [DMSO], and 0.05% Triton as final concentrations) was added to each well for a total volume of 150 μl. Luminescence of each well was read 3 min after adding luciferin. During incubation with luciferin, the microplates were shaken at a medium setting. Background measurements of luminescence were subtracted from readings. Luminescence readings were normalized to GFP fluorescence, which was measured using a 528/20-nm emission filter.

### Measurement of p-AMPK levels

Activated p-AMPK levels were measured as previously described [126]. Worm samples for each genotype, condition, and time point were prepared by removing the supernatant. Pelleted worms were mixed with 1 volume of 2× sample loading buffer (200 mm Tris-Cl [pH 8.0], 500 mm NaCl, 0.1 mm EDTA, 0.1% Triton X-100, and 0.4 mm phenylmethylsulfonyl fluoride) and boiled for 10 min by immersion in a water bath. Worm lysates were electrophoresed on a 4–20% precast SDS-polyacrylamide gel (Mini-Protean TGX Gels, Bio-Rad) and electroblotted onto a nitrocellulose membrane (Trans-Blot Turbo Transfer Pack, Bio-Rad) using a Trans-Turbo Blot transfer system (Bio-Rad). The membrane was incubated in a blocking solution containing the p-AMPKα Thr 172 antibody (Cell Signaling Technologies, Cat #2535S, 1:1,000 dilution) or the β-actin antibody (Millipore, Cat #MAB1501R, 1:3,000 dilution) and rocked at 4°C overnight, followed by incubation with an anti-mouse antibody (Invitrogen, Cat #7076S, 1:5,000 dilution) or anti-rabbit horseradish peroxidase antibody (Jackson ImmunoResearch, Cat #7074S, 1:5,000 dilution) for 1 hr at room temperature. The ECL western blotting system (Clarity Western ECL Substrate) was used to detect the secondary antibodies on the membrane. Luminescence of the blot was visualized and captured using the Chemidoc V3 Touch Western Imager for mini-gels (Bio-Rad). ImageJ version 1.51h (NIH) [123] was used to quantify the intensity of p-AMPK and actin bands. We were unable to specifically detect total AMPK levels in worm samples using AMPKα (23A2) (Cat #2603), AMPKα1 (Cat #2795), AMPKα2 (Cat #2757), AMPKβ1 (71C10) (Cat #4178), or AMPKγ1–3 (Cat # 4187, 2536, 2550) antibodies from Cell Signaling Technologies.

### Western blots of anti-GFP in KIN-29(WT)::GFP and KIN-29(S517A)::GFP

Measurements of KIN-29(WT)::GFP and KIN-29(S517A)::GFP were conducted by cultivating about 6,000–7,000 age-synchronized worms using the double-bleaching method [125], transferred to NGM agar surface (6-cm diameter) that was fully covered with a lawn of *E*. *coli* OP50, and grown at 20°C. L1 animals were washed off the agar surface using 5 ml of M9 buffer. The worm and bacterial suspension was allowed to settle through a 5-μm nylon mesh filter, which passes bacteria but traps the worms. The worms trapped by the filter were then flash frozen in liquid N_2_ and stored at −80°C until analysis. Worms were collected at 14, 17, and 20 hr after feeding, which corresponds to before, during, and after L1 lethargus, respectively. Western blots were conducted as described above, and the rabbit monoclonal antibody GFP (D5.1) at 1:5,000 dilution (Cell Signaling Technology Cat # 2956) was used to detect GFP.

### Measurement of body size

Body-length measurements were carried out by acquiring digital images of adult worms 24 hr after the L4 larval molt at 100× magnification. The length of the worm was traced with short line segments using Leica LAS software, and the sum of the line lengths was calculated. The tail was not included in the measurements.

### Dye-filling

A stock dye solution containing 5 mg/μl red fluorescent lipophilic dye DiI (Sigma Aldrich) was diluted in M9 buffer by 10,000 times for optimal signal intensity. Animals carrying the transgene *Podr-4*::*atp-3(sas)* were soaked in the fluorescent dye solution for 1 hr and then rinsed with M9 buffer twice. Stained animals were recovered for 1 hr on NGM plates seeded with *E*. *coli* OP50 bacterial food before examination of sensory neurons with microscopy.

### Optogenetics

Optogenetic activation of RIS was conducted by exposing animals carrying the transgene *Paptf-1*::*ChR2*::*mkate2* to blue light using the GFP filter of a Leica stereomicroscope equipped with a Leica EL6000 light source. L4 animals were transferred to plates seeded with DA837 *E*. *coli* bacteria supplemented with either 100 mM ATR dissolved in EtOH or EtOH vehicle alone and incubated overnight in the dark at 20°C. While monitored at 5–12× objective lens, the pumping rate of young adult worms was counted for 10 s prior to exposure to blue light, 10 s while exposed to blue light, and 10 s after blue-light exposure. ATR plates were utilized within a week of seeding with *E*. *coli* and were stored in the dark until use.

### Histamine-mediated chemogenetic sleep deprivation

For sleep deprivation experiments, worms expressing *Pflp-11*::*HisCl* were placed on NGM agar containing 10 mM histamine hydrochloride (Sigma, Cat # H2750) immediately prior to experiments. For SIS experiments, worms were age-synchronized using the bleaching method [127] and grown on NGM agar plates seeded with either *E*. *coli* DA837 or OP50 until worms reached adulthood. One-day-old adults were transferred individually onto the agar surface of individual wells of a WorMotel PDMS chip filled with either NGM agar supplemented with histamine dissolved in water (10 mM final concentration of histamine) or water vehicle. Within 15 min, worms were UV irradiated (1,500 J/m^2^ UVC irradiation, 254 nm) while on the WorMotel chip, and movement quiescence (min) was recorded for approximately 8–10 hr as described above. For DTS experiments, mid-L4 animals were transferred to the WorMotel chip with NGM agar supplemented with either histamine dissolved in water (10 mM) or water vehicle, and body movement quiescence (min) was recorded for approximately 15–20 hr during L4 lethargus as described above.

### PHX treatment

PHX (100 mM) (Sigma, Cat #SML0120) in DMSO solution was diluted to 1 mM using DMSO (final DMSO concentration of 1%). One hundred microliters was spotted on the agar surface of *E*. *coli* OP50-seeded plates and allowed to dry. For SIS experiments, L4 larvae were exposed for about 12 hr to PHX. One-day-old adults were transferred individually to WorMotel wells filled with NGM agar supplemented with either 1 mM PHX in 1% DMSO or 1% DMSO vehicle. Worms were UV irradiated (1,500 J/m^2^ UVC irradiation, 254 nm) within 15 min of transfer, and movement quiescence was recorded for approximately 8–10 hr as described above. For DTS experiments, late-L2-stage larvae were exposed to plates containing PHX approximately 12 hr prior to experiments. Early-L4-stage worms were transferred individually to the WorMotel wells containing NGM agar supplemented with either 1 mM PHX in 1% DMSO or DMSO vehicle. Animals were then recorded for 15–20 hr, which were later analyzed to obtain body movements quiescence measurements as described above.

### Subcellular localization and quantification of KIN-29(WT)::GFP and KIN-29(S517A)::GFP

An asynchronous population of gravid adult worms were treated with alkaline bleach, and the progeny were allowed to enter the L1 diapause stage for 12 hr before resuming development by feeding them. For each transgenic line (PY5790 and NQ1241) and time point, between 1,500 and 2,500 worms were plated on 60-mm NGM agar plates seeded with bacteria. Worms were collected in 40% isopropanol and fixed for 1 min at room temperature on a nutator. Worms were sedimented by centrifugation at 800 rcf for 1 min. The supernatant was removed and 200 μl of 4,6-diamidino-2-phenylindole (DAPI) staining solution (2 ng/μl in PBST) was added to the worm pellet. Worms were allowed to stain for 2 min in the dark with nutation and pelleted by centrifugation at 1,200 rcf for 1 min. The supernatant was removed and worms were washed 1× with PBST to remove excess DAPI. Worm were transferred to an agar pad, and image z-stacks (between 10 images with a step size of 0.7 μm at 60× magnification) were captured with a Leica SP8 confocal microscope in a sequential fashion (alternated between GFP and DAPI filters).

To quantify subcellular localization of KIN-29::GFP, we used 2 approaches. In the first approach, an experimenter blinded to the genotype/condition of the worm scored each worm on a scale of 0–3, where 0 denotes fully cytoplasmic and 3 denotes fully nuclear location of the GFP. In the second approach, we quantified KIN-29::GFP using ImageJ software (NIH) by comparing the fluorescence intensity in the cytoplasm with the fluorescence intensity in the nucleus of imaged sensory neurons. Cytoplasmic and nuclear regions of each cell were determined using both the green and blue channels to show cells expressing the transgene (GFP) and nuclear staining (DAPI). A region of interest (ROI) was drawn around the cytoplasm of each cell, excluding the nucleus, and intensity was quantified on the green channel only. An additional ROI was then drawn around the nucleus of each cell, and the intensity was quantified also on the green channel. The nuclear intensity was divided into the cytoplasmic intensity to produce a nuclear:cytoplasmic ratio; higher values are indicative of greater nuclear localization of the transgene, whereas lower values are indicative of greater cytosolic localization of the transgene.

### Quiescence quantification for KIN-29(NLS)

Movement quiescence was measured using a 24-well (4 × 6) WorMotel. In the evening prior to the assay, early- to mid-L4 animals were plated on the agar surface of standard 6-cm-diameter petri dishes filled with NGM agar and plated with OP50 bacteria. Early adult animals were then transferred to a WorMotel and imaged for the first 4 hr after transfer, this period has been shown to have an absence of RIS activation on live bacteria [32]. The worms were imaged under dark-field illumination provided by a red LED strip. Images were captured every 10 s for the duration of recording using approximately 8.5-μm/pixel spatial resolution. Images were analyzed by the frame subtraction method [38] using custom Matlab software (https://github.com/cfangyen/wormotel). Movement quiescence was defined as a lack of changed pixels between successive frames.

### Statistical analysis

Data were graphed and analyzed using Graphpad Prism 8 software. Data sets were first analyzed for Gaussian distribution using a D’Agostino-Pearson or Shapiro-Wilk normality test (alpha = 0.05, *p* > 0.05). If a normality test was passed, then a parametric statistical test was performed. If a normality test was not passed, then a nonparametric statistical test was used. Statistical comparisons made include the unpaired *t* test (parametric, 2 groups), the unpaired Mann-Whitney *t* test (2-tailed, nonparametric, 2 groups), the ordinary 1-way ANOVA followed by a Tukey multiple-comparisons test (parametric, for more than 2 groups), or the Kruskal-Wallis test followed by a Dunn multiple-comparisons test (nonparametric, for more than 2 groups). For time-series experiments, we used a 2-way ANOVA or comparable mixed-effects analysis when there was a missing value followed by a Bonferroni, Tukey, or Sidak multiple-comparisons test. Specific statistical tests and *p*-values are reported in the figure legends.

## Supporting information

S1 Fig Total ATP and p-AMPK levels in *aak-2* and *pink-1* mutants. (A) Percent change in total protein (μg) and ATP (nM) levels during L1 development. Data are normalized to its baseline value at 13 hr and represented as the mean ± SEM of 4 experiments for the protein time course and 4 experiments for the ATP time course (S2 Data, Sheet S1A). (B) ATP extinction curve of wild-type L4 animals (see Material and methods) as a function of time after sample extraction (*n* > 50 for each time point) (S2 Data, Sheet S1B). (C) Correlations between normalized ATP and of the time-based change in normalized ATP (delta ATP) with the fraction of animals in lethargus. Colored lines indicate the best fit of replicates (*n* = 5) as determined by a linear regression model. Best linear fit for each time course is indicated by R^2^ (S2 Data, Sheet S1C). (D) Quantification of p-AMPK levels in L4 animals of *aak-2* null mutants with representative western blots, in which the intensity of the bands represents p-AMPK using antibodies for the mammalian p-AMPKα Thr-172 (top panel) and β-actin (lower panel) as a loading control. Data are normalized to wild-type and represented as the mean ± SEM of 2 experiments. ***p < 0.001 by an unpaired 2-tailed *t* test. A sequence alignment of AMPK proteins:phosphorylation site. AAK-2, the worm homolog of the AMPKα subunit, is regulated by phosphorylation of Threonine-243 (purple), which corresponds to the Threonine-172 of human AMPKα (S2 Data, Sheet S1D). (E and F) Total body ATP per μg protein (E) and p-AMPK normalized to the actin loading control) (F) measured in L4 animals of wild-type controls and *pink-1* mutants. Data are normalized to wild-type controls and represented as the mean ± SEM of 6 experiments for ATP and 2–3 experiments for p-AMPK. **p < 0.01 by an unpaired 2-tailed *t* test (E) and *p < 0.05 by a 2-tailed Mann-Whitney *t* test (F). Representative western blots are shown of wild-type and *pink-1* mutants in which the intensity of the bands represents p-AMPK (top panel) and β-actin (lower panel) as a loading control (S2 Data, Sheet S1E-F). AMPK, adenosine monophosphate regulated protein kinase; L1, first larval stage; L4, fourth larval stage; p-AMPK, phosphorylated AMPK; *pink-1*, Pten induced putative kinase 1.
(TIF)

S2 Fig Movement quiescence, ATP, and AMPK levels during sleep deprivation. (A and B) Chemogenetic silencing of RIS neurons results in a reduction in body movement quiescence during L4 lethargus/DTS (A) and after UVC exposure/SIS (B) in WT animals expressing the *Pflp-11*::*HisCl* transgene (2 independent transgenic lines, NQ1208 and NQ1209) in the presence of 10 mM histamine (+His), and in the absence of histamine (-His). The *flp-11* promoter is expressed in RIS. Left graphs: Time course with minutes of movement quiescence in 10-min bins during L4 lethargus/DTS and minutes of movement quiescence in 1-hr bins after UVC irradiation (1,500 J/m^2^). Statistical comparisons were performed with a 2-way ANOVA using time and genotype as factors, followed by post hoc pairwise comparisons at each time point to obtain nominal *p*-values, which were subjected to a Bonferroni correction for multiple comparisons. ***, **, and * indicate corrected *p*-values that are different from transgenic animals (-His) at *p* < 0.001, *p* < 0.01, and *p* < 0.05, respectively. Right graphs: Total minutes of movement quiescence during L4 lethargus/DTS (A) and movement quiescence during the first 4 hr after UVC irradiation (1,500 J/m^2^) (B) determined from the time-course data. Data are represented as mean ± SEM. *p < 0.05 by an unpaired 2-tailed *t* test (E) and ***p < 0.001 by an ANOVA with Tukey multiple-comparisons test (F) (S2 Data, Sheet S2A and S2B). (C) Replication of the experiment shown in Fig 1G. Total body ATP levels per μg protein were measured 0 and 2 hr after UVC exposure in adults animals expressing either the *Pflp-11*::*HisCl* transgene (NQ1209), WT adults in the presence of 10 mM histamine (+His), and WT adults in the absence of histamine (-His). Data were normalized to WT controls immediately before UVC exposure (0 hr) in the absence of histamine (-His). The graph shows the mean ± SEM of 2 biologically independent experiments with *n* = 2–6 technical replicates for each condition and time point. *** and ** indicate values that are different from that of nontransgenic animals (+His) at *p* < 0.001 and *p* < 0.01, respectively. Statistical comparisons were performed with a 2-way ANOVA using time and genotype as factors, followed by a Tukey correction for multiple comparisons (S2 Data, Sheet S2C). (D) Total p-AMPK normalized by the actin loading control measured at maximum quiescence (2 hr) after UVC exposure in WT animals and animals expressing the *Pflp-11*::*HisCl* transgene (NQ1209) in the presence of 10 mM histamine (+His) and in the absence of histamine (-His). Data were normalized to WT controls immediately before UVC exposure (0 hr) in the absence of histamine (-His). The graph shows the mean ± SEM of 2 experiments. *p < 0.05 by an ANOVA with Tukey multiple-comparisons test. A representative western blot is shown adjacent to the graph (S2 Data, Sheet S2D). AMPK, adenosine monophosphate regulated protein kinase; DTS, developmentally timed sleep; L1, first larval stage; L4, fourth larval stage; ns, not significant; p-AMPK, phosphorylated AMPK; SIS, stress-induced sleep; UVC, ultraviolet C; WT, wild type.
(TIF)

S3 Fig Quiescence, total ATP, and p-AMPK levels in *kin-29* mutants. (A) SIK phylogeny tree. The bootstrap values in percentages of each branch are denoted in red. All alignments were performed with a maximum-likelihood method MEGA X (www.megasoftware.net) with 1,000 bootstrap replicates after removing poorly aligned sequence regions (N- and C-terminal parts). Sequences (UniProtKB) used were as follows: SIK3 *Drosophila* (Q4QQA7), SIK2 *Drosophila* (O77268), SIK1 mouse (Q60670), SIK2 mouse (Q8CFH6), SIK3 mouse (Q6P4S6), SIK1 human (P57059), SIK2 human (Q9H0K1), SIK3 human (Q9Y2K2), and KIN-29 *C*. *elegans* (Q21017). (B) Heatmap of the fraction of movement quiescence of 7 wild-type and 7 *kin-29* null mutants during L4 lethargus/DTS. Each row in the heatmap represents an individual animal recorded by video over an approximately 20-hr period. The time of each worm’s record was adjusted to align to the start of L4 lethargus quiescence. See Material and methods. (C) Fraction of quiescence of a wild-type and *kin-29*-mutant individual animal during L4 lethargus/DTS. The time of the 2 worms’ records was adjusted to align to the start of the L4 lethargus quiescence. The brief episodes of quiescence of the *kin-29* mutant prior to L4 lethargus likely reflect times when the animal transiently left the field of view (S2 Data, Sheet S3C). (D) Feeding rate of wild-type and *kin-29*-mutant animals measured 2 hr after UVC irradiation (1,500 J/m^2^). Horizontal line denotes the mean ± SEM (*n* = 12–20 animals). ***p < 0.01 by a 2-tailed Mann-Whitney *t* test (S2 Data, Sheet S3D). (E and F) Body movement quiescence (E) is reduced and feeding rate (F) is increased after heat shock/SIS is reduced in *kin-29-*mutant animals in comparison with wild type. Adult animals were heat-shocked at 35°C for 30 min (see Material and methods). Graphs show the mean ± SEM of *n* = 19–23 animals for movement quiescence and *n* = 9–18 animals for feeding rate. ***p < 0.001, **p < 0.01, *p < 0.05 by a 2-way ANOVA with Bonferroni’s multiple-comparisons test (E) and mixed-effects analysis with a Bonferroni multiple-comparisons test (F) (S2 Data, Sheet S3E and S3F). (G) Bioluminescence in wild-type and *kin-29*-mutant adult animals carrying the *sur-5*::*luciferase*::*gfp* reporter. Luminescence was normalized to *gfp* expression emanating from the same transgene. Data are represented as the mean ± SD of 6–7 replicates. **p < 0.01, by an unpaired 2-tailed *t* test (S2 Data, Sheet S3G). (H) Quantification and representative western blots of p-AMPK in L4 larvae of wild-type and *kin-29* mutants. β-actin (lower panel) is a loading control. Data are normalized to wild-type and represented as the mean ± SEM of 3 experiments. **p < 0.01 by an unpaired 2-tailed *t* test (S2 Data, Sheet S3H). (I) Total body ATP per μg protein in wild-type and *kin-29*-mutant animals at 0 and 2 hr after induction of SIS by UVC exposure. Data were normalized to wild-type controls immediately before UVC exposure (0 hr) and are represented as the mean ± SEM of 4–6 experiments, **p < 0.01 by a 2-way ANOVA with Sidak’s multiple-comparisons test (S2 Data, Sheet S3I). AMPK, adenosine monophosphate regulated protein kinase; DTS, developmentally timed sleep; *gfp*, green fluorescent protein; L4, fourth larval stage; ns, not significant; p-AMPK, phosphorylated AMPK; SIK, salt-inducible kinase; SIS, stress-induced sleep; UVC, ultraviolet C.
(TIF)

S4 Fig Fat content, food-uptake behavior, and food-leaving behavior in *kin-29* mutants. (A) Food-leaving behavior measured in wild-type controls, *kin-29-*, and *eat-2*-mutant animals. *kin-29* mutants have increased food-leaving behavior similar to *eat-2* feeding-defective mutants. Food leaving was quantified as the area of exploration, with each data point representing tracks from a population outside the bacterial lawn. Each data point represents the total number of pixels outside of the bacterial lawn of 7 animals per plate, and the horizontal line represents the mean ± SEM of individual experiments. Left: Frames from a 12-hr video were collapsed in a single image for food-leaving behavior. Scale is 0.5 cm. ***p < 0.001 by an ANOVA with Tukey multiple-comparisons test (S2 Data, Sheet S4A). (B) Fat content measured with fixative Oil Red O staining for wild-type and *kin-29* mutants. Data are represented as a percentage of total body fat in wild-type controls ± SEM (*n* = 12 animals for each genotype and developmental stage). *** and ** indicate values that are different from wild type at *p* < 0.001 and *p* < 0.01, respectively, by an unpaired 2-tailed *t* test (S2 Data, Sheet S4B). Representative images are shown of animals fixed and stained with Oil Red O at different developmental stages from L1 larvae to young adults of wild-type and *kin-29* mutants. Scale is 15 μm. (C) Lipid droplet morphology of *kin-29* mutants. Top panel: Animals mutant for *kin-29* result in an increased lipid droplet number and size. Expression of *kin-29* in *odr-4*-expressing neurons restores the increased lipid droplet number of *kin-29* mutants. Lipid droplet number and size is quantified within a 50- or 18-μm^2^ area, respectively, in the anterior intestine. Lipid number: ns or ***p < 0.001 by an ANOVA with Tukey multiple-comparisons test. Lipid size: **p < 0.01 by a 2-tailed Mann-Whitney *t* test. Middle panel: The distribution and average size of lipid droplets in wild type and *kin-29* mutants (S2 Data, Sheet S4C). Lower panel: Representative images are shown of animals expressing DHS-3::GFP in wild type and *kin-29* mutants. DHS-3::GFP stains the membrane of lipid droplets in gut cells. Scale is 10 μm. (D) Microsphere uptake measured in wild-type controls, *kin-29*, and *eat-2-*mutant animals. Animals mutant for *kin-29* have normal food uptake, unlike *eat-2* feeding-defective mutants. Representative images are shown of single adult wild-type, *kin-29-*, and *eat-2*-mutant animals that accumulated 1.0-μm fluorescent microsphere beads for 15 min at 20°C. The arrows indicate the terminal bulbs of the pharynxes. Insets show DIC images of animals. Data are represented as the mean ± SEM (*n* = 10 animals). ***p < 0.001 by a Kruskal-Wallis with Dunn multiple-comparisons test (S2 Data, Sheet S4D). DIC, differential interference contrast; L1, first larval stage; ns, not significant.
(TIF)

S5 Fig ATP levels and fat stores in *aptf-1* mutants. (A) Levels of total body ATP normalized by μg protein of wild-type and *aptf-1*-mutant animals measured before, during, and after L1 lethargus/DTS. Graphs show the mean ± SD of 1 experiment for wild type and 1 experiment for *aptf-1* mutants with 3 technical replicates for each. *** and * indicate corrected *p*-values that are different from wild type at *p* < 0.001 and *p* < 0.05, respectively. Statistical comparisons were performed with a mixed-effects analysis using time and genotype as factors, followed by post hoc pairwise comparisons at each time point to obtain nominal *p*-values, which were subjected to a Bonferroni correction for multiple comparisons (S2 Data, Sheet S5A). (B) Fat content measured with fixative Oil Red O staining for wild-type and *aptf-1*-mutant animals. Data are represented as a percentage of total body fat in wild-type controls ± SEM (*n* = 25 animals for each genotype). Representative images are shown of animals fixed and stained with Oil Red O. Scale is 15 μm. DTS, developmentally timed sleep; L1, first larval stage; ns, not significant as determined by an unpaired 2-tailed *t* test (S2 Data, Sheet S5B).
(TIF)

S6 Fig Rescue of the reduced DTS and SIS of *kin-29* mutants by ATGL-1 OE. (A) ATGL-1 OE restores in part the defect in DTS body movement quiescence of *kin-29* L4 null mutants. Data are represented as a moving window of the fraction of a 10-min time interval spent quiescent of *n* = 3–6 animals for each trace. The x-axis represents hours from the start of recording in the late L4 stage. The data from individual worms were aligned such that the start of lethargus quiescence occurred simultaneously. Shading indicates SEM (S2 Data, Sheet S6A). (B) Time course of minutes of quiescence in 1-hr bins after UVC irradiation (1,500 J/m^2^). Data are represented as the mean ± SEM (*n* = 8–10 animals for each genotype). Statistical comparisons were performed with a 2-way ANOVA using time and genotype as factors, followed by post hoc pairwise comparisons at each time point to obtain nominal *p*-values, which were subjected to a Bonferroni correction for multiple comparisons. ** indicates corrected *p*-values that are different from WT at *p* < 0.01 (S2 Data, Sheet S6B). (C) Representative images shown of animals fixed and stained with Oil Red O of WT and *kin-29* null mutants with or without the ATGL-1 OE transgene. Scale is 15 μm. (D) Frames from a 12-hr video collapsed in a single image for food-leaving behavior of WT and *kin-29* null mutants with or without the ATGL-1 OE transgene. *n* = 5 animals in each image. Scale is 0.5 cm. (E) Movement quiescence during L4 lethargus/DTS of animals overexpressing ATGL-1. Data are represented as a moving window of the fraction of a 10-min time interval spent quiescent of *n* = 4–7 animals for each trace. The x-axis represents hours from the start of recording in the late L4 stage. The data from individual worms were aligned such that the start of lethargus quiescence occurred simultaneously. Shading indicates SEM. Right graph: Total minutes of quiescence in lethargus are represented as the mean ± SEM with *n* = 4–7 animals for DTS. (F) Time course of minutes of quiescence in 1-hr bins after UVC irradiation (1,500 J/m^2^). Data are represented as the mean ± SEM (*n* = 14–24 animals for each genotype). Animals overexpressing ATGL-1 are not significantly different from WT as determined by a 2-way ANOVA using time and genotype as factors, followed by post hoc pairwise comparisons at each time point to obtain nominal *p*-values, which were subjected to a Bonferroni correction for multiple comparisons. Right graph: Total minutes quiescent in SIS are displayed as mean ± SEM (*n* = 14–24 animals for each genotype). ATGL-1, adipose triglyceride lipase-1; DTS, developmentally timed sleep; L4, fourth larval stage; ns, not significant as determined by an unpaired *t* test (S2 Data, Sheet S6E; S2 Data, Sheet S6F); OE, overexpression; SIS, stress-induced sleep; UVC, ultraviolet C; WT, wild type.
(TIF)

S7 Fig PHX treatment reduces DTS and SIS in wild-type animals. (A) Schematic showing that the CPT inhibitor PHX blocks beta-oxidation of fatty acids in mitochondria to promote the accumulation of lipids. (B) PHX (1 mM) reduces body movement quiescence during L4 lethargus/DTS in wild-type animals in comparison with vehicle controls (-PHX). Data are represented as a moving window of the fraction of a 10-min time interval spent quiescent of *n* = 9 animals for the PHX(-) trace and *n* = 11 animals for the PHX(+) trace. The x-axis represents hours from the start of recording in the late L4 stage. The data from individual worms were aligned such that the start of lethargus quiescence occurred simultaneously. Shading indicates SEM (S2 Data, Sheet S7A). (C and D) Time course of minutes of quiescence in 1-hr bins (C) and feeding rate (in pumps per 10 sec) (D) after UVC irradiation (1,500 J/m^2^) in the absence (-) and presence (+) of PHX. Data are represented as the mean ± SEM with *n* = 10 animals for movement quiescence (C) and *n* = 27–28 animals for feeding quiescence (D) for the PHX(-) and PHX(+) condition. Statistical comparisons were performed with a 2-way ANOVA using time and PHX conditions as factors, followed by post hoc pairwise comparisons at each time point to obtain nominal *p*-values, which were subjected to a Bonferroni correction for multiple comparisons. ** and *** indicate corrected *p*-values that are different from wild-type at *p* < 0.01, and *p* < 0.001, respectively (S2 Data, Sheet S7C and S7D). CPT, carnitine palmitoyltransferase; DTS, developmentally timed sleep; L4, fourth larval stage; PHX, perhexiline; SIS, stress-induced sleep; UVC, ultraviolet C.
(TIF)

S8 Fig Rescue of *kin-29*-mutant phenotypes by expressing *kin-29* in sensory neuron types. (A) Representative images of animals fixed and stained with Nile Red (left images, scale is 15 μm) and food-leaving behavior (black/white image, scale is 0.5 cm) of adult animals expressing the *Podr-4*::*kin-29* or *Pges-1*::*kin-29* transgene. *odr-4*, chemosensory promoter. *ges-1*, intestinal promoter. Frames from a 12-hr video were collapsed in a single image for food-leaving behavior. *n* = 7 animals in each image. (B) Time course of minutes of quiescence in 1-hr bins after UVC irradiation (1,500 J/m^2^). Data are represented as mean ± SEM (*n* = 14–32 animals). Statistical comparisons were performed with a 2-way ANOVA using time and genotype as factors, followed by post hoc pairwise comparisons at each time point to obtain nominal *p*-values, which were subjected to a Bonferroni correction for multiple comparisons. *** and * indicate corrected *p*-values that are different from *kin-29* mutants at *p* < 0.001 and *p* < 0.05, respectively (S2 Data, Sheet S8B). (C) Levels of total body ATP levels normalized by μg protein in wild-type and *kin-29* null mutant animals expressing the *Podr-4*::*kin-29* transgene measured before, during, and after L1 lethargus/DTS. The graphs show the mean ± SD of 1 experiment for wild type with 2 technical replicates and 1 experiment for *kin-29* animals with 3 technical replicates that carry the extrachromosomal *Podr-4*::*kin-29* transgene (PY5791). Of note, PY5791 includes about 20% nontransgenic *kin-29* animals. The second y-axis shows the averaged fraction of nonpumping animals (*n* = 10) for each genotype and time point. Animals expressing *Podr-4*::*kin-29* are not significantly different from wild type, as determined by a 2-way ANOVA using time and genotype as factors, followed by a Bonferroni multiple-comparison test (S2 Data, Sheet S8C). DTS, developmentally timed sleep; *ges-1*, gut esterase 1; L1, first larval stage; UVC, ultraviolet C.
(TIF)

S9 Fig Rescue of *kin-29*-mutant phenotypes by expressing *kin-29* in individual or a set of sensory neuron types. (A) Schematic showing the transgenic rescue strategy used to restore *kin-29* expression in a subset of *odr-4*-expressing sensory neurons (left) and in individual neurons (right). (B) Expression and localization of a *srh-56* promoter (approximately 1.5 kb) fusion with GFP. A black and white image shows *Psrh-56*::*gfp* fluorescence in ASK, ASH, and ASJ neurons plus other nonneuronal cells. Scale is 10 μm. (C) Representative images of a single L4 larvae fixed and stained with Oil Red O of wild-type and *kin-29* null mutant animals with or without the *Psrh-56*::*kin-29* transgene. Scale is 15 μm. (D) Time course of minutes of quiescence in 1-hr bins after UVC irradiation (1,500 J/m^2^) of wild type and *kin-29* null mutants with or without the *Podr-3*::*kin-29* transgene. Data are represented as the mean ± SEM (*n* = 14–15 animals). Statistical comparisons were performed with a mixed-effects analysis using time and genotype as factors, followed by post hoc pairwise comparisons at each time point to obtain nominal *p*-values, which were subjected to a Bonferroni correction for multiple comparisons. *** and ** indicate corrected *p*-values that are different from *kin-29* mutants at *p* < 0.001 and *p* < 0.01, respectively (S2 Data, Sheet S9D). (E) Fraction of quiescence of *kin-29* null mutants expressing *kin-29* under control of the *odr-3* (AWA, AWB, AWC, ADF, ASH), *gpa-4* (ASI), *srh-142* (ADF), and *sre-1* (ADL) promoter. Data are represented in a 10-min time interval of *n* = 3–5 animals for each sleep trace. The x-axis represents hours from the start of recording in the late L4 stage. The data from individual worms were aligned such that the start of lethargus quiescence occurred simultaneously. Shading indicates SEM (S2 Data, Sheet S9E). GFP, green fluorescent protein; L4 stage, fourth larval stage; UVC, ultraviolet C.
(TIF)

S10 Fig RNAi knockdown of *atp-3* in sensory neurons but not in the intestine leads to anachronistic movement quiescence. (A) Individual traces of the fraction of quiescence of *Podr-4*::*atp-3(sas)* animals and wild-type control animals. The fraction of quiescence in a 10-min moving window is shown for each trace (*n* = 7–9 animals). The x-axis represents hours from the start of recording at the young adult stage (S2 Data, Sheet S10A). (B) Representative image of an animal carrying the *Podr-4*::*atp-3(sas)* transgene, which has a reduced body size compared with a wild-type animal. Scale is 50 μm. (C) Representative images of dye-filling of sensory neurons of a wild-type and *Podr-4*::*atp-3(sas)* transgenic animal. Scale is 10 μm. (D) Levels of total body ATP normalized by μg protein measured in young adults of wild-type control animals and *Pges-1*::*atp-3(sas)* transgenic animals. Data are normalized to wild-type controls and are represented as the mean ± SEM of 3 experiments. *p < 0.05 by an unpaired *t* test (S2 Data, Sheet S10D). (E) Averaged traces of the fraction of quiescence of *Pges-1*::*atp-3(sas)* animals (*n* = 9) and wild-type control animals (*n* = 10). Data are represented as a moving window of the fraction of a 10-min time interval spent quiescent for each genotype. The x-axis represents hours from the start of recording at the young adult stage. Shading indicates SEM (S2 Data, Sheet S10E). (F) *isp-1* single mutants are highly quiescent compared with *isp-1; kin-29* double-mutant animals after heat shock/SIS. Adult animals were heat-shocked at 35°C for 20 min. Minutes of body movement quiescence of *isp-1* (*n* = 10) and *isp-1; kin-29*-mutant animals (*n* = 7). Shading indicates SEM. *p < 0.05 by a 2-way ANOVA with Bonferroni’s multiple-comparisons test (S2 Data, Sheet S10F).; RNAi, RNA interference; sas, sense and antisense.
(TIF)

S11 Fig RIS is required for SIS, and *kin-29* mutants do not attenuate the quiescence of FLP-13 OE. (A and B) Body movement quiescence (A) and feeding rate (B) of *kin-29* null mutants after induction of FLP-13 OE. To induce FLP-13 OE, adult animals expressing a *Phsp-16*.*2*::*FLP-13* transgene were heat-shocked for 30 min (see Material and methods). Data are represented as the mean ± SEM with *n* = 24 animals for movement quiescence (A) and *n* = 14–17 animals for feeding quiescence. Feeding rate in pumps per 10 s was determined 2 hr after heat shock. ns and ***p < 0.001 by a 2-tailed Mann-Whitney *t* test (A) and an ANOVA with Tukey multiple-comparisons test (B) (S2 Data, Sheet S11A and S11B). (C and D) Body movement quiescence following either UVC irradiation 1,500 J/m^2^ (C) or heat shock at 35°C for 30 min (D). Data are represented as mean ± SEM (*n* > 10 animals for each genotype). Left graphs: For the time-course experiments, statistical comparisons were performed with a 2-way ANOVA using time and genotype as factors, followed by post hoc pairwise comparisons at each time point to obtain nominal *p*-values, which were subjected to a Bonferroni correction for multiple comparisons. ***, **, and * indicate corrected *p*-values that are different from wild type at *p* < 0.001, *p* < 0.01, and *p* < 0.05, respectively. Right graphs: Total minutes of movement quiescence during 8 hr after UVC irradiation (C) and 1 hr after heat shock (D) determined from the time-course data. Data are represented as mean ± SEM. ns, ***p < 0.001, **p < 0.01, and *p < 0.05 by a Kruskal-Wallis with Dunn multiple-comparisons test. Lower 2 graphs of S11D Fig: Replication experiment of movement quiescence following heat shock at 35°C for 30 min of *ceh-17-* and *aptf-1*-mutant animals compared with wild type. Left panel: 2-way ANOVA with Bonferroni correction for multiple comparisons. Right panel: 1-way ANOVA with Tukey multiple comparisons; ***p < 0.001, **p < 0.01, *p < 0.05, and ns (S2 Data, Sheet S11C and S11D). (E and F) Feeding rate following either UVC irradiation 1,500 J/m^2^ (E) or heat shock at 35°C for 30 min (F). Feeding rate in pumps per 10 s was determined 2 hr after UV irradiation (E) or heat shock (F). ***p < 0.001, **p < 0.01, and *p < 0.05 by a Kruskal-Wallis with Dunn multiple-comparisons test (S2 Data, Sheet S11E and S11F).; ns, not significant; OE, overexpression; SIS, stress-induced sleep; UVC, ultraviolet C.
(TIF)

S12 Fig KIN-29(S517A) mutant rescues the small-body-size phenotype of *kin-29*. (A) Sequence alignment of SIK3 proteins (human Q9Y2K2, mouse Q6P4S6, *Drosophila* Q4QQA7, *C*. *elegans* Q21017). The conserved PKA phosphorylation site serine is shown in blue. (B) Quantification of KIN-29(WT)::GFP and KIN-29(S517A)::GFP in a subcellular compartment of *odr-4*(+) neurons before, during, and after lethargus/DTS of the L1 stage. Higher values are indicative of greater nuclear localization of the transgene, whereas lower values are indicative of greater cytosolic localization of the transgene (see Material and methods). Data are represented as the mean ± SEM. **p < 0.01 and *p < 0.05 by an ANOVA with Tukey multiple-comparisons test (S2 Data, Sheet S12B). (C) KIN-29(S517A) remains cytosolic after heat shock. Shown is KIN-29(WT)::GFP in the nucleus (arrow) of an *odr-4*-expressed neuron after heat shock (left image). KIN-29(S517A)::GFP remains in cytoplasmic after heat shock (right image). Scale is 10 μm. (D) KIN-29(S517A) rescues the small body size of *kin-29* null mutants. Shown is the relative body length of adult animals (*n* = 8–13) of the indicated genotypes. Data are represented as the mean ± SEM. ns and ***p < 0.001 by an ANOVA with Tukey multiple-comparisons test (S2 Data, Sheet S12D). (E) Western blot analysis of KIN-29::GFP in *Podr-4*::*kin-29(WT)*::*GFP* and *Podr-4*::*kin-29(S517A)*::*GFP* transgenic strains before, during, and after lethargus/DTS of the L1 stage (S2 Data, Sheet S12E). KIN-29::GFP levels were overall lower in *kin-29(S517A)*::*GFP*, as evident both by lower *gfp* fluorescence in this strain and reduced anti-GFP staining. This difference may be explained by different copy numbers of the transgene or by different stability of KIN-29(S517A) in comparison with KIN-29(WT). Levels of KIN-29::GFP protein (top panel) using anti-GFP antibodies are indicated with β-actin (lower panel) as a loading control. DTS, developmentally timed sleep; GFP, green fluorescent protein; KIN-29(WT), wild-type KIN-29; L1 stage, first larval stage; ns, not significant; PKA, Protein Kinase A; SIK3, salt-inducible kinase 3.
(TIF)

S13 Fig Mutations in *hda-4* restore *kin-29* mutant sleep and foraging phenotypes. (A) Time course of minutes of quiescence in 1-hr bins after UVC irradiation (1,500 J/m^2^) of single and double mutants between *kin-29* and *hda-4* compared with wild type. Data are represented as the mean ± SEM (*n* > 10 animals). Statistical comparisons were performed with a 2-way ANOVA using time and genotype as factors, followed by post hoc pairwise comparisons at each time point to obtain nominal *p*-values, which were subjected to a Bonferroni correction for multiple comparisons. ***, **, and * indicate corrected *p*-values that are different from wild type at *p* < 0.001, *p* < 0.01, and *p* < 0.05, respectively (S2 Data, Sheet S13A). (B) Time course of minutes of quiescence in 1-hr bins after UVC irradiation (1,500 J/m^2^) of animals expressing *hda-4* under the control of its own promoter and under the control of the *odr-4* chemosensory neuron specific promoter. Data are represented as the mean ± SEM (*n* > 10 animals). Statistical comparisons were performed with a 2-way ANOVA using time and genotype as factors, followed by post hoc pairwise comparisons at each time point to obtain nominal *p*-values, which were subjected to a Bonferroni correction for multiple comparisons *** and ** indicate corrected *p*-values that are different from wild type at *p* < 0.001 and *p* < 0.01, respectively (S2 Data, Sheet S13B). (C) Food-leaving behavior of wild-type, single-mutant, and double-mutant animals of *kin-29* and *hda-4* quantified as the area of exploration, with each data point representing tracks from a population outside the bacterial lawn. Each data point represents the total number of pixels outside of the bacterial lawn of 7 animals per plate, and the horizontal line represents the mean ± SEM of individual experiments. Right images: Frames from a 12-hr video were collapsed in a single image for food-leaving behavior. Scale is 0.5 cm. *** indicates values that are different from wild type, *kin-29*, or *hda-4* single mutants at *p* < 0.001 by an ANOVA with Tukey multiple-comparisons test (S2 Data, Sheet S13C). (D and E) Levels of total body ATP normalized by μg protein (D) and total body p-AMPK normalized to the actin loading control (E) in wild type and *kin-29 hda-4* double mutants measured before, during, and after L1 lethargus/DTS. ATP levels in wild-type worms are not significantly different from *kin-29 hda-4* double mutants as determined by a 2-way ANOVA multiple comparison with Bonferroni correction. Shown is a representative time course of ATP and p-AMPK measured from the same samples. Data are normalized to the average value of the wild-type time course. Graphs show the mean ± SD for wild type and *kin-29 hda-4* doubles. The second y-axis shows the averaged fraction of nonpumping animals (*n* = 10) for each genotype and time point (S2 Data, Sheet S13D and S13E). (F) Quantification and representative western blots of p-AMPK in L4 larvae of wild type and single and double mutants of *kin-29* and *hda-4*. Data are normalized to wild type. ** and * indicate values that are different from wild type or *kin-29* mutants at *p* < 0.01 and *p* < 0.05, respectively, by an ANOVA with Tukey multiple-comparisons test. A western blot is shown below to the graph with p-AMPK (top panel) and β-actin (lower panel) as a loading control (S2 Data, Sheet S13F). DTS, developmentally timed sleep; *hda-4*, histone deacetylase 4; L1, first larval stage; L4, fourth larval stage; ns, not significant; p-AMPK, phosphorylated adenosine monophosphate regulated protein kinase; UVC, ultraviolet C.
(TIF)

S14 Fig Expression of KIN-29 in sensory neuron nuclei promotes sleep. (A) Schematic of the generated KIN-29(NLS)::GFP construct (see Material and methods) for the constitutive expression of KIN-29 in the nuclei of *odr-4*-expressing sensory neurons, or under the control of the *hsp-16*.*2* promoter. KIN-29(WT) is the control construct without NLS. (B) Representative images of KIN-29(WT)::GFP in the cytosol (left image) and KIN-29(NLS)::GFP in the nucleus (right image) of *odr-4*-expressed sensory neurons. The arrow indicates a cell of the *odr-4*(+) neurons. Scale is 10 μm. (C) Individual traces of the fraction of quiescence of *Podr-4*::*kin-29(NLS)*::*GFP* transgenic animals and wild-type controls animals. The fraction of quiescence in a 10-min moving window is shown for each trace over a 4-hr period. The x-axis represents hours from the start of recording at the young adult stage (S2 Data, Sheet S14C). (D) Body movement quiescence is increased in *Phsp-16*.*2*::*kin-29(NLS)* transgenic animals after heat shock/SIS in comparison with control wild-type and *hsp*::*kin-29* transgenic animals. Adult animals were heat-shocked at 35°C for 20 min. Graphs show the mean ± SEM of wild-type (*n* = 20), *Phsp-16*.*2*::*kin-29(NLS)* (*n* = 10), and *Phsp-16*.*2*::*kin-29* (*n* = 22) animals for movement quiescence. Statistical comparisons were performed with a mixed-effects analysis using time and genotype as factors, followed by post hoc pairwise comparisons at each time point to obtain nominal *p*-values, which were subjected to a Tukey multiple-comparisons test. ** and * indicate corrected *p*-values that are different from *Phsp-16*.*2*::*kin-29* expressing animals at *p* < 0.01 and *p* < 0.05, respectively (S2 Data, Sheet S14D). (E) Mutations in *aptf-1* suppress the increased movement quiescence of animals expressing *Phsp-16*.*2*::*kin-29(NLS)* after heat shock/SIS in comparison with wild-type control. Adult animals were heat-shocked at 35°C for 20 min. Minutes of body movement quiescence of wild-type (*n* = 18) and *aptf-1; Phsp-16*.*2*::*kin-29(NLS)* (*n* = 16) animals. Shading indicates SEM. ***p < 0.001 and *p < 0.05 by a 2-way ANOVA with Bonferroni’s multiple-comparisons test (S2 Data, Sheet S14E). KIN-29(WT), wild-type KIN-29; NLS, nuclear localization signal; SIS, stress-induced sleep.
(TIF)

S1 Table List of strains used in this study. (XLSX)

S2 Table List of oligonucleotides used in this study. (XLSX)

S3 Table List of constructs used in this study. (XLSX)

S1 Data Raw data for all experiments from the main figures (Figs 1–9). (XLSX)

S2 Data Raw data for all experiments from the supporting figures (S1–S14 Figs). (XLSX)

S1 Movie A video of an awake adult wild-type animal in a single well of a WorMotel. The video is sped up 8 times.
(MP4)

S2 Movie A video of an adult animal in a single well of a WorMotel displaying anachronistic movement quiescence induced by expressing *kin-29* constitutively in sensory nuclei under control of the odr-4 promoter. The video is sped up 8 times.
(MP4)

## Abbreviations

AMPK: adenosine monophosphate regulated protein kinase
ATGL-1: adipose triglyceride lipase-1
ATR: all-*trans* retinal
ChR2: channelrhodopsin2
CPT: carnitine palmitoyltransferase
DTS: developmentally timed sleep
EGF: epidermal growth factor
EGFR: EGF receptor
FOXO: forkhead box protein O
ges-1: gut esterase 1
GFP: green fluorescent protein
HDA-4: histone deacetylase 4
HisCl: histamine-gated chloride channel
KIN-29(WT): wild-type KIN-29
L1: first larval stage
L4: fourth larval stage
MEF-2: myocyte enhancer factor 2
NLS: nuclear localization signal
ns: not significant
p-AMPK: phosphorylated AMPK
PHX: perhexiline
PKA: Protein Kinase A
PKG: Protein Kinase G
pink-1: Pten induced putative kinase 1
sas: sense and antisense
SIK: Salt-Inducible Kinase
SIS: stress-induced sleep
TAG: triacylglyceride
UVC: ultraviolet C
