# Supplementary figures and images for: A salt-induced kinase is required for the metabolic regulation of sleep
Source: PLoS Biol. 2020 Apr 21;18(4):e3000220. doi: 10.1371/journal.pbio.3000220 (PMC7173979; doi:10.1371/journal.pbio.3000220)

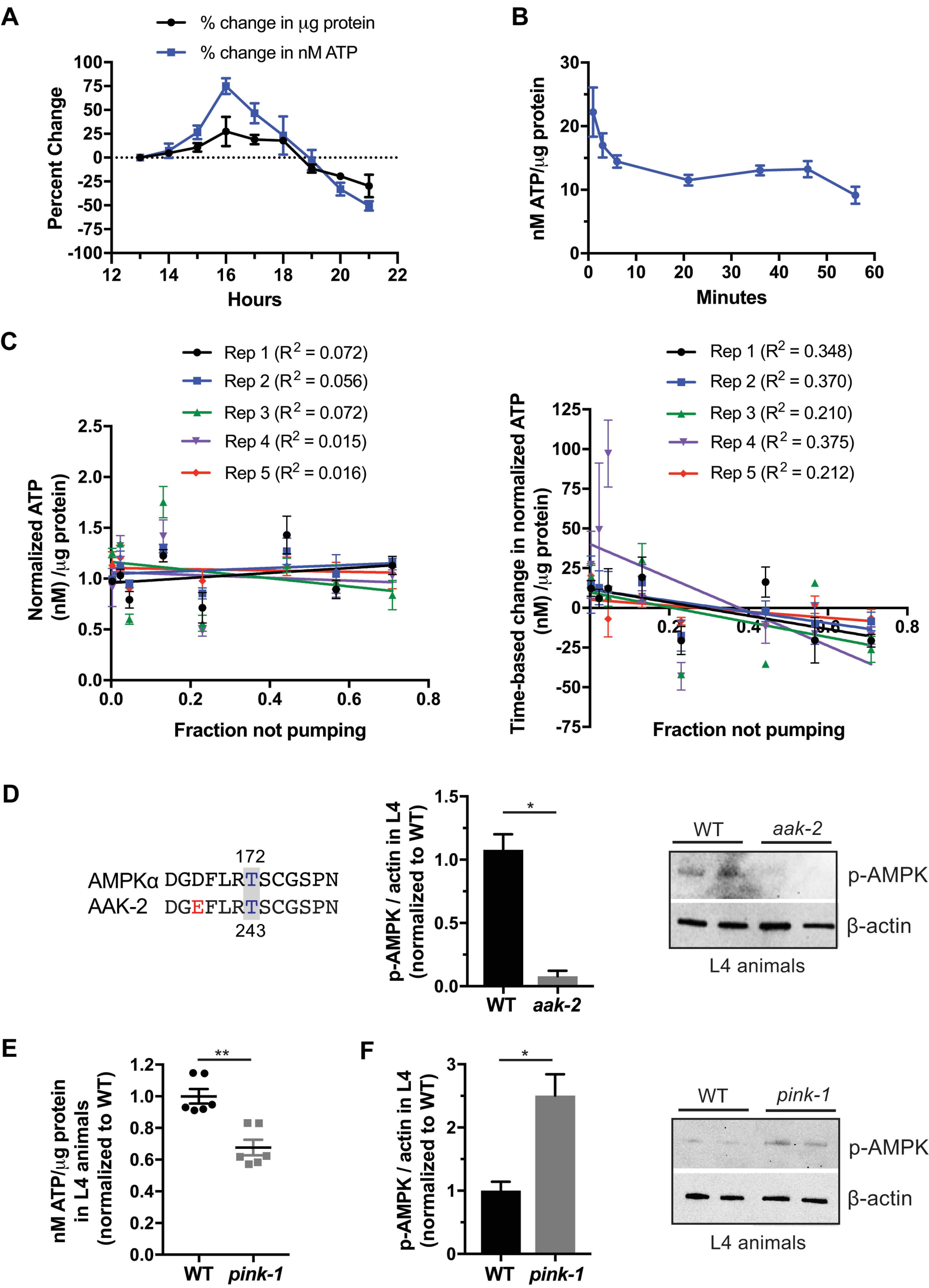

Supplement: S1 Fig — (A) Percent change in total protein (μg) and ATP (nM) levels during L1 development. Data are normalized to its baseline value at 13 hr and represented as the mean ± SEM of 4 experiments for the protein time course and 4 experiments for the ATP time course (S2 Data, Sheet S1A). (B) ATP extinction curve of wild-type L4 animals (see Material and methods) as a function of time after sample extraction (n > 50 for each time point) (S2 Data, Sheet S1B). (C) Correlations between normalized ATP and of the time-based change in normalized ATP (delta ATP) with the fraction of animals in lethargus. Colored lines indicate the best fit of replicates (n = 5) as determined by a linear regression model. Best linear fit for each time course is indicated by R2 (S2 Data, Sheet S1C). (D) Quantification of p-AMPK levels in L4 animals of aak-2 null mutants with representative western blots, in which the intensity of the bands represents p-AMPK using antibodies for the mammalian p-AMPKα Thr-172 (top panel) and β-actin (lower panel) as a loading control. Data are normalized to wild-type and represented as the mean ± SEM of 2 experiments. ***p < 0.001 by an unpaired 2-tailed t test. A sequence alignment of AMPK proteins:phosphorylation site. AAK-2, the worm homolog of the AMPKα subunit, is regulated by phosphorylation of Threonine-243 (purple), which corresponds to the Threonine-172 of human AMPKα (S2 Data, Sheet S1D). (E and F) Total body ATP per μg protein (E) and p-AMPK normalized to the actin loading control) (F) measured in L4 animals of wild-type controls and pink-1 mutants. Data are normalized to wild-type controls and represented as the mean ± SEM of 6 experiments for ATP and 2–3 experiments for p-AMPK. **p < 0.01 by an unpaired 2-tailed t test (E) and *p < 0.05 by a 2-tailed Mann-Whitney t test (F). Representative western blots are shown of wild-type and pink-1 mutants in which the intensity of the bands represents p-AMPK (top panel) and β-actin (lower panel) as a loading control (S2 [file pbio.3000220.s001.tif]

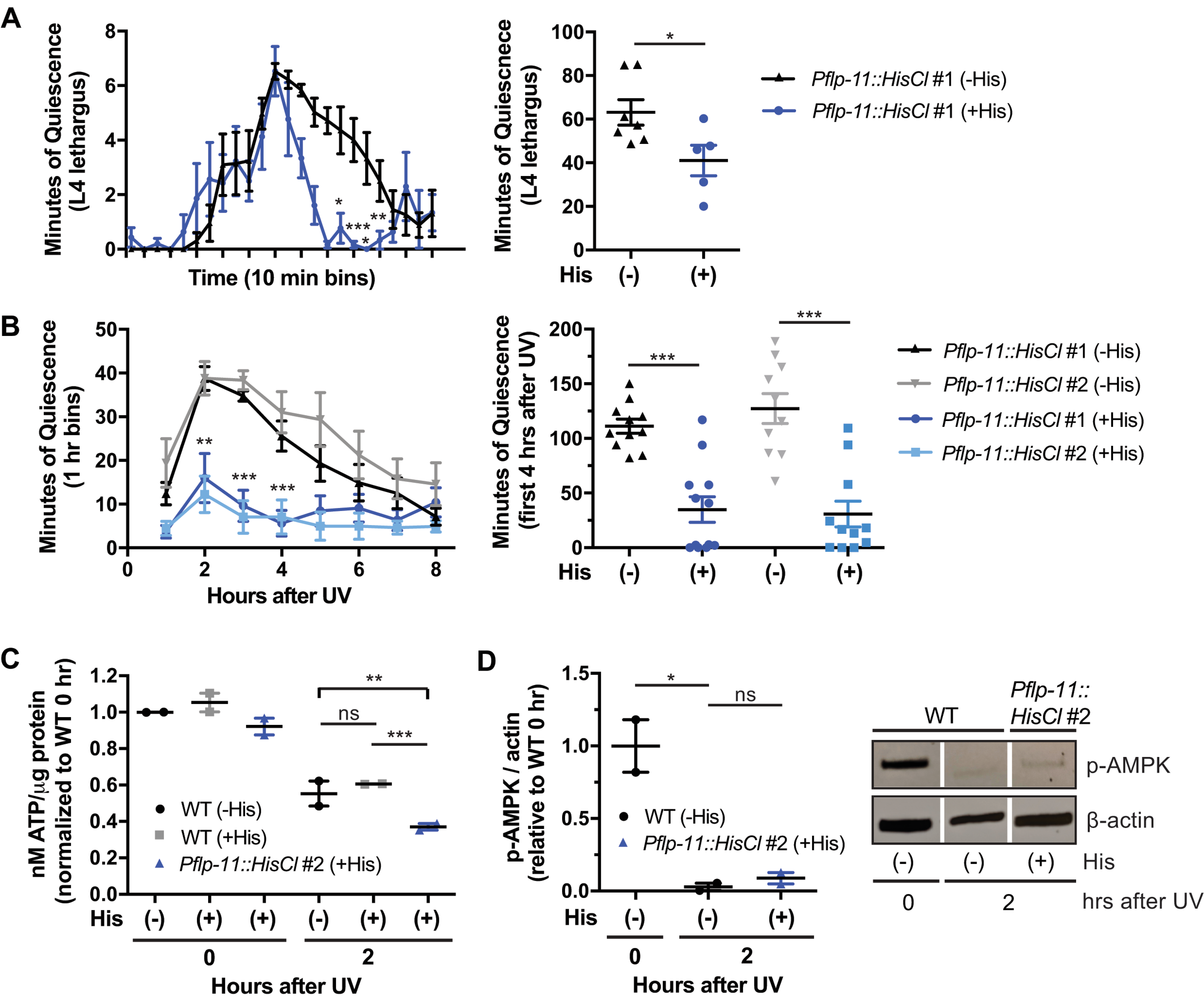

Supplement: S2 Fig — (A and B) Chemogenetic silencing of RIS neurons results in a reduction in body movement quiescence during L4 lethargus/DTS (A) and after UVC exposure/SIS (B) in WT animals expressing the Pflp-11::HisCl transgene (2 independent transgenic lines, NQ1208 and NQ1209) in the presence of 10 mM histamine (+His), and in the absence of histamine (-His). The flp-11 promoter is expressed in RIS. Left graphs: Time course with minutes of movement quiescence in 10-min bins during L4 lethargus/DTS and minutes of movement quiescence in 1-hr bins after UVC irradiation (1,500 J/m2). Statistical comparisons were performed with a 2-way ANOVA using time and genotype as factors, followed by post hoc pairwise comparisons at each time point to obtain nominal p-values, which were subjected to a Bonferroni correction for multiple comparisons. ***, **, and * indicate corrected p-values that are different from transgenic animals (-His) at p < 0.001, p < 0.01, and p < 0.05, respectively. Right graphs: Total minutes of movement quiescence during L4 lethargus/DTS (A) and movement quiescence during the first 4 hr after UVC irradiation (1,500 J/m2) (B) determined from the time-course data. Data are represented as mean ± SEM. *p < 0.05 by an unpaired 2-tailed t test (E) and ***p < 0.001 by an ANOVA with Tukey multiple-comparisons test (F) (S2 Data, Sheet S2A and S2B). (C) Replication of the experiment shown in Fig 1G. Total body ATP levels per μg protein were measured 0 and 2 hr after UVC exposure in adults animals expressing either the Pflp-11::HisCl transgene (NQ1209), WT adults in the presence of 10 mM histamine (+His), and WT adults in the absence of histamine (-His). Data were normalized to WT controls immediately before UVC exposure (0 hr) in the absence of histamine (-His). The graph shows the mean ± SEM of 2 biologically independent experiments with n = 2–6 technical replicates for each condition and time point. *** and ** indicate values that are different from that of nontransgenic animals [file pbio.3000220.s002.tif]

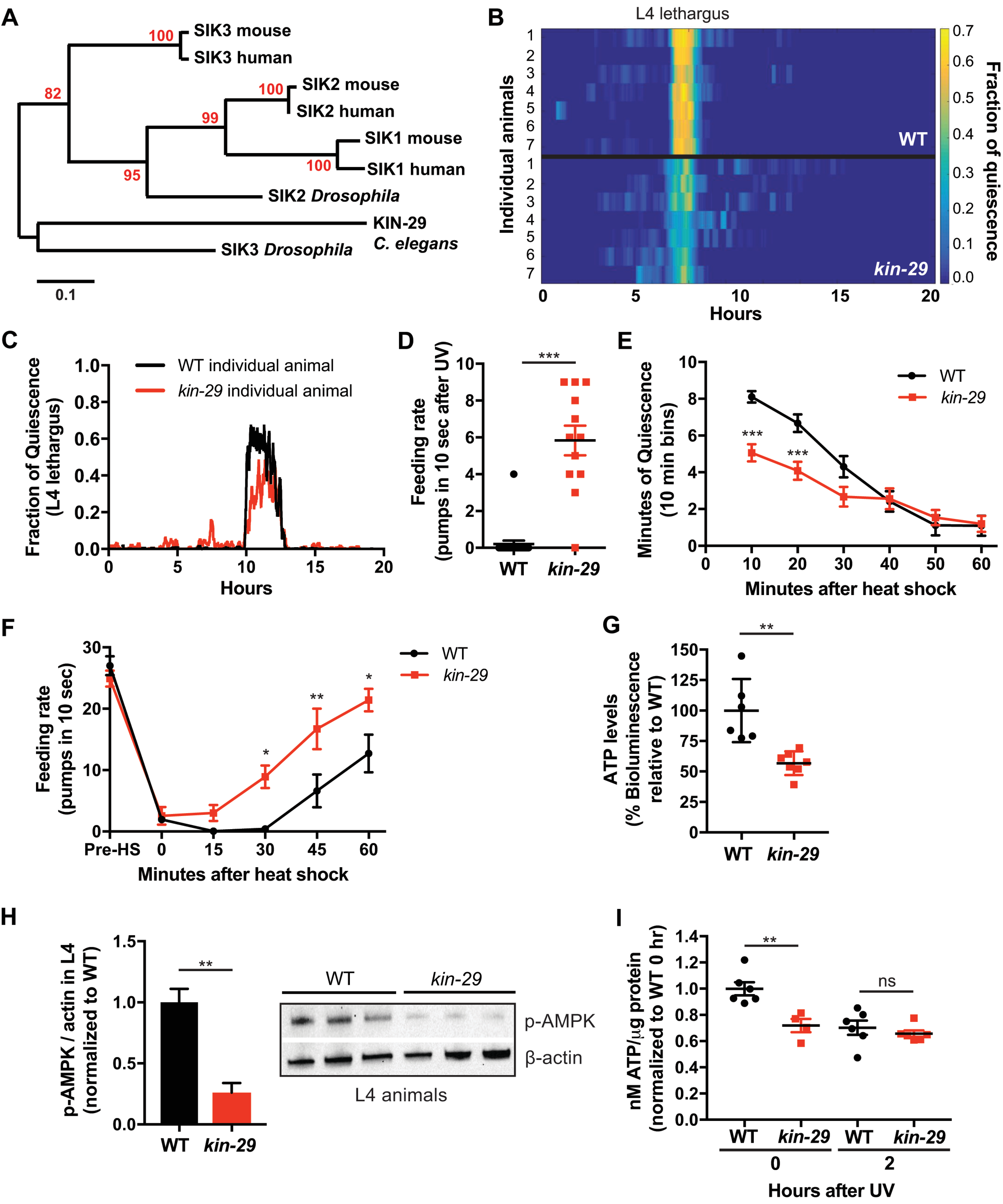

Supplement: S3 Fig — (A) SIK phylogeny tree. The bootstrap values in percentages of each branch are denoted in red. All alignments were performed with a maximum-likelihood method MEGA X (www.megasoftware.net) with 1,000 bootstrap replicates after removing poorly aligned sequence regions (N- and C-terminal parts). Sequences (UniProtKB) used were as follows: SIK3 Drosophila (Q4QQA7), SIK2 Drosophila (O77268), SIK1 mouse (Q60670), SIK2 mouse (Q8CFH6), SIK3 mouse (Q6P4S6), SIK1 human (P57059), SIK2 human (Q9H0K1), SIK3 human (Q9Y2K2), and KIN-29 C. elegans (Q21017). (B) Heatmap of the fraction of movement quiescence of 7 wild-type and 7 kin-29 null mutants during L4 lethargus/DTS. Each row in the heatmap represents an individual animal recorded by video over an approximately 20-hr period. The time of each worm’s record was adjusted to align to the start of L4 lethargus quiescence. See Material and methods. (C) Fraction of quiescence of a wild-type and kin-29-mutant individual animal during L4 lethargus/DTS. The time of the 2 worms’ records was adjusted to align to the start of the L4 lethargus quiescence. The brief episodes of quiescence of the kin-29 mutant prior to L4 lethargus likely reflect times when the animal transiently left the field of view (S2 Data, Sheet S3C). (D) Feeding rate of wild-type and kin-29-mutant animals measured 2 hr after UVC irradiation (1,500 J/m2). Horizontal line denotes the mean ± SEM (n = 12–20 animals). ***p < 0.01 by a 2-tailed Mann-Whitney t test (S2 Data, Sheet S3D). (E and F) Body movement quiescence (E) is reduced and feeding rate (F) is increased after heat shock/SIS is reduced in kin-29-mutant animals in comparison with wild type. Adult animals were heat-shocked at 35°C for 30 min (see Material and methods). Graphs show the mean ± SEM of n = 19–23 animals for movement quiescence and n = 9–18 animals for feeding rate. ***p < 0.001, **p < 0.01, *p < 0.05 by a 2-way ANOVA with Bonferroni’s multiple-comparisons test (E) and mixed-effects analysis with a Bo [file pbio.3000220.s003.tif]

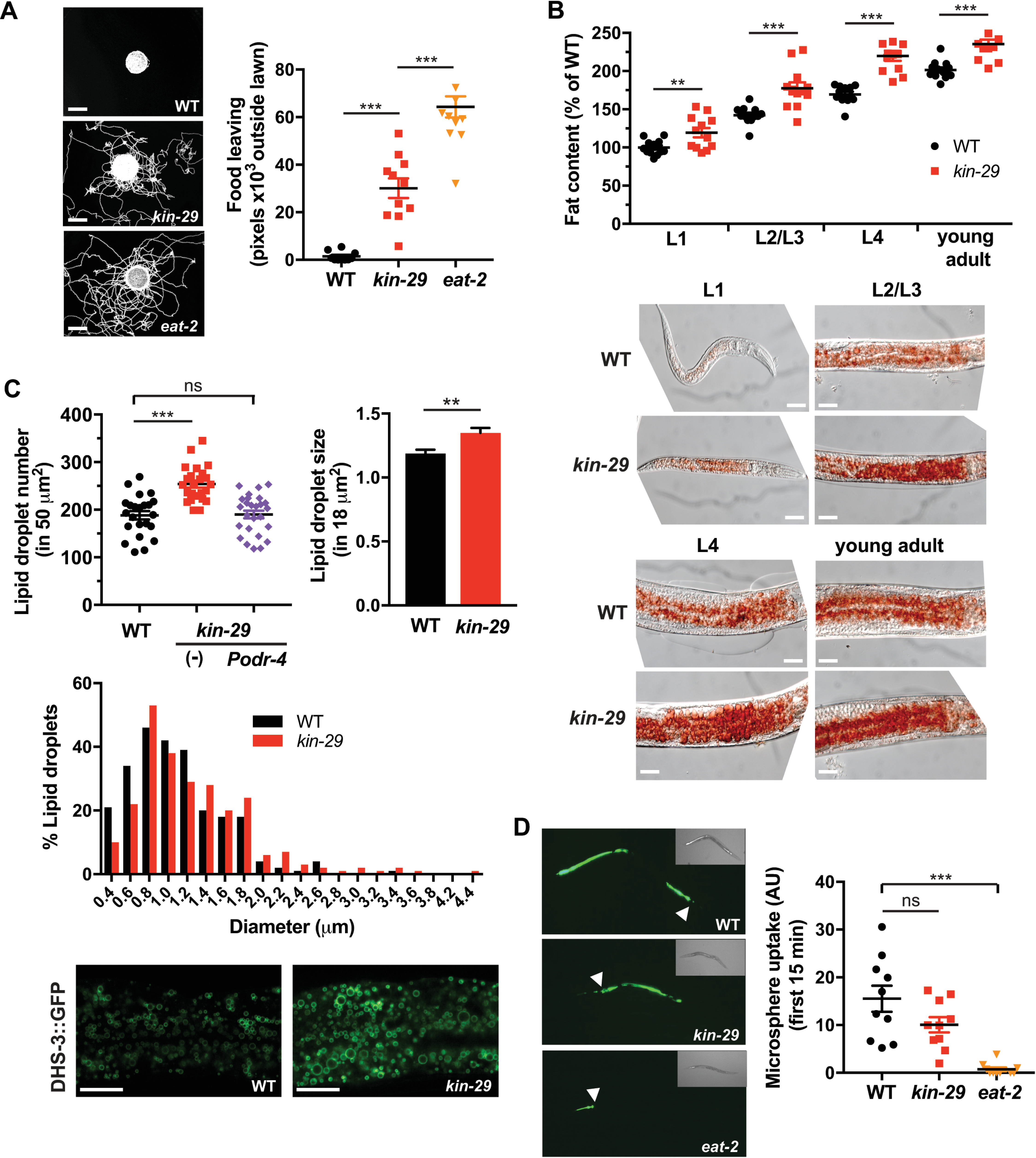

Supplement: S4 Fig — (A) Food-leaving behavior measured in wild-type controls, kin-29-, and eat-2-mutant animals. kin-29 mutants have increased food-leaving behavior similar to eat-2 feeding-defective mutants. Food leaving was quantified as the area of exploration, with each data point representing tracks from a population outside the bacterial lawn. Each data point represents the total number of pixels outside of the bacterial lawn of 7 animals per plate, and the horizontal line represents the mean ± SEM of individual experiments. Left: Frames from a 12-hr video were collapsed in a single image for food-leaving behavior. Scale is 0.5 cm. ***p < 0.001 by an ANOVA with Tukey multiple-comparisons test (S2 Data, Sheet S4A). (B) Fat content measured with fixative Oil Red O staining for wild-type and kin-29 mutants. Data are represented as a percentage of total body fat in wild-type controls ± SEM (n = 12 animals for each genotype and developmental stage). *** and ** indicate values that are different from wild type at p < 0.001 and p < 0.01, respectively, by an unpaired 2-tailed t test (S2 Data, Sheet S4B). Representative images are shown of animals fixed and stained with Oil Red O at different developmental stages from L1 larvae to young adults of wild-type and kin-29 mutants. Scale is 15 μm. (C) Lipid droplet morphology of kin-29 mutants. Top panel: Animals mutant for kin-29 result in an increased lipid droplet number and size. Expression of kin-29 in odr-4-expressing neurons restores the increased lipid droplet number of kin-29 mutants. Lipid droplet number and size is quantified within a 50- or 18-μm2 area, respectively, in the anterior intestine. Lipid number: ns or ***p < 0.001 by an ANOVA with Tukey multiple-comparisons test. Lipid size: **p < 0.01 by a 2-tailed Mann-Whitney t test. Middle panel: The distribution and average size of lipid droplets in wild type and kin-29 mutants (S2 Data, Sheet S4C). Lower panel: Representative images are shown of animals expressing DHS-3::GFP in wil [file pbio.3000220.s004.tif]

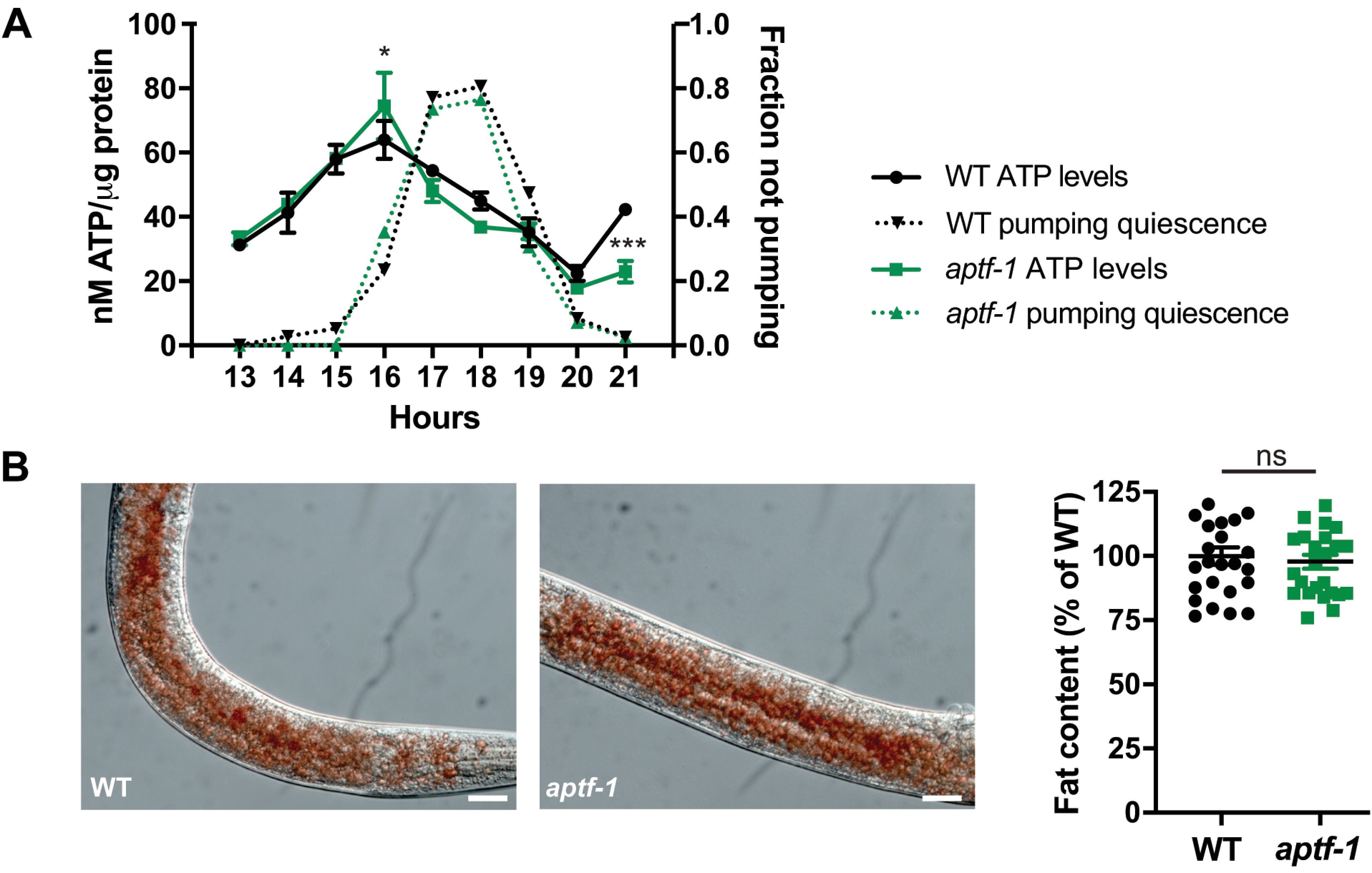

Supplement: S5 Fig — (A) Levels of total body ATP normalized by μg protein of wild-type and aptf-1-mutant animals measured before, during, and after L1 lethargus/DTS. Graphs show the mean ± SD of 1 experiment for wild type and 1 experiment for aptf-1 mutants with 3 technical replicates for each. *** and * indicate corrected p-values that are different from wild type at p < 0.001 and p < 0.05, respectively. Statistical comparisons were performed with a mixed-effects analysis using time and genotype as factors, followed by post hoc pairwise comparisons at each time point to obtain nominal p-values, which were subjected to a Bonferroni correction for multiple comparisons (S2 Data, Sheet S5A). (B) Fat content measured with fixative Oil Red O staining for wild-type and aptf-1-mutant animals. Data are represented as a percentage of total body fat in wild-type controls ± SEM (n = 25 animals for each genotype). Representative images are shown of animals fixed and stained with Oil Red O. Scale is 15 μm. DTS, developmentally timed sleep; L1, first larval stage; ns, not significant as determined by an unpaired 2-tailed t test (S2 Data, Sheet S5B). (TIF) [file pbio.3000220.s005.tif]

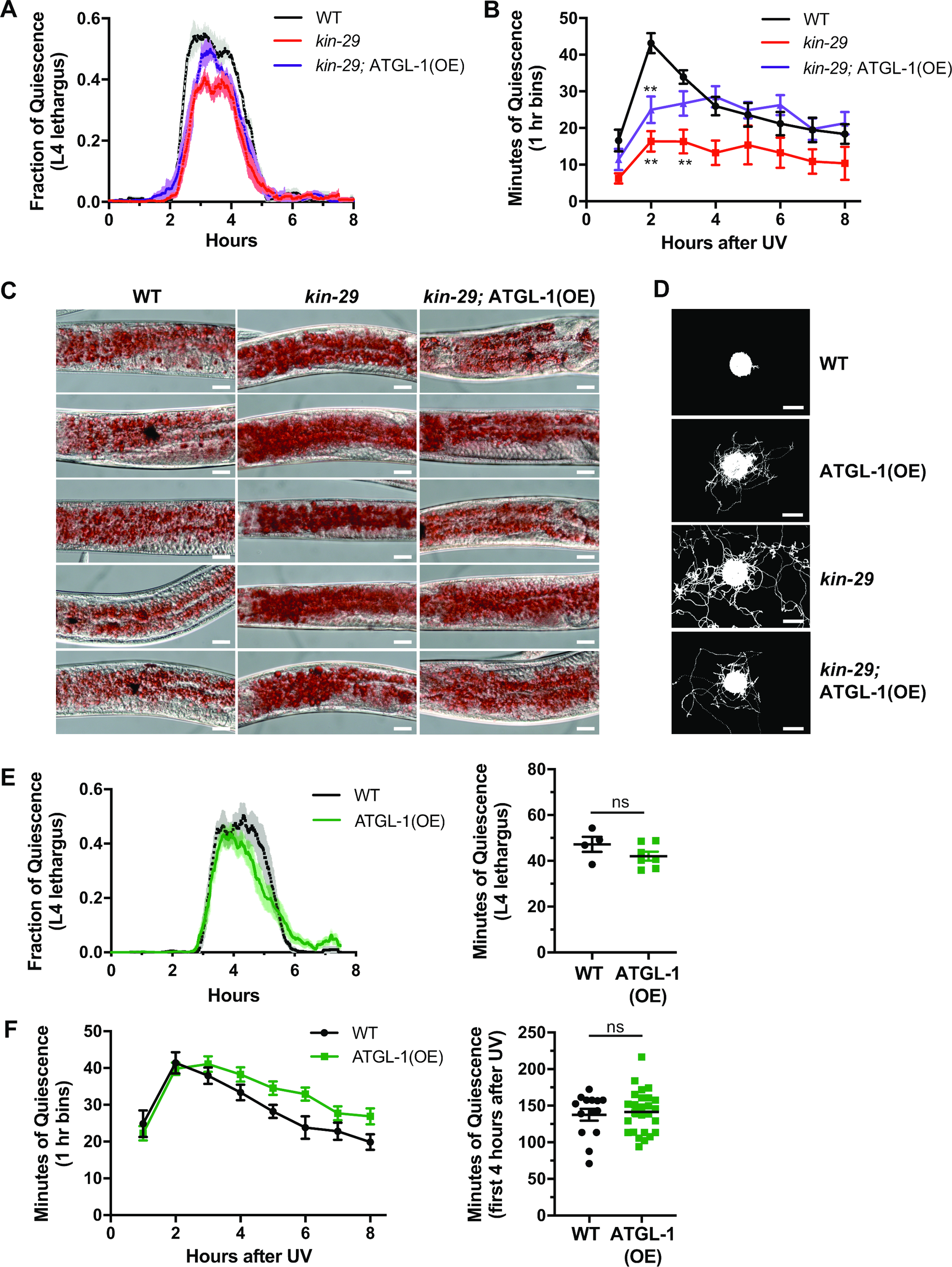

Supplement: S6 Fig — (A) ATGL-1 OE restores in part the defect in DTS body movement quiescence of kin-29 L4 null mutants. Data are represented as a moving window of the fraction of a 10-min time interval spent quiescent of n = 3–6 animals for each trace. The x-axis represents hours from the start of recording in the late L4 stage. The data from individual worms were aligned such that the start of lethargus quiescence occurred simultaneously. Shading indicates SEM (S2 Data, Sheet S6A). (B) Time course of minutes of quiescence in 1-hr bins after UVC irradiation (1,500 J/m2). Data are represented as the mean ± SEM (n = 8–10 animals for each genotype). Statistical comparisons were performed with a 2-way ANOVA using time and genotype as factors, followed by post hoc pairwise comparisons at each time point to obtain nominal p-values, which were subjected to a Bonferroni correction for multiple comparisons. ** indicates corrected p-values that are different from WT at p < 0.01 (S2 Data, Sheet S6B). (C) Representative images shown of animals fixed and stained with Oil Red O of WT and kin-29 null mutants with or without the ATGL-1 OE transgene. Scale is 15 μm. (D) Frames from a 12-hr video collapsed in a single image for food-leaving behavior of WT and kin-29 null mutants with or without the ATGL-1 OE transgene. n = 5 animals in each image. Scale is 0.5 cm. (E) Movement quiescence during L4 lethargus/DTS of animals overexpressing ATGL-1. Data are represented as a moving window of the fraction of a 10-min time interval spent quiescent of n = 4–7 animals for each trace. The x-axis represents hours from the start of recording in the late L4 stage. The data from individual worms were aligned such that the start of lethargus quiescence occurred simultaneously. Shading indicates SEM. Right graph: Total minutes of quiescence in lethargus are represented as the mean ± SEM with n = 4–7 animals for DTS. (F) Time course of minutes of quiescence in 1-hr bins after UVC irradiation (1,500 J/m2). Data are repr [file pbio.3000220.s006.tif]

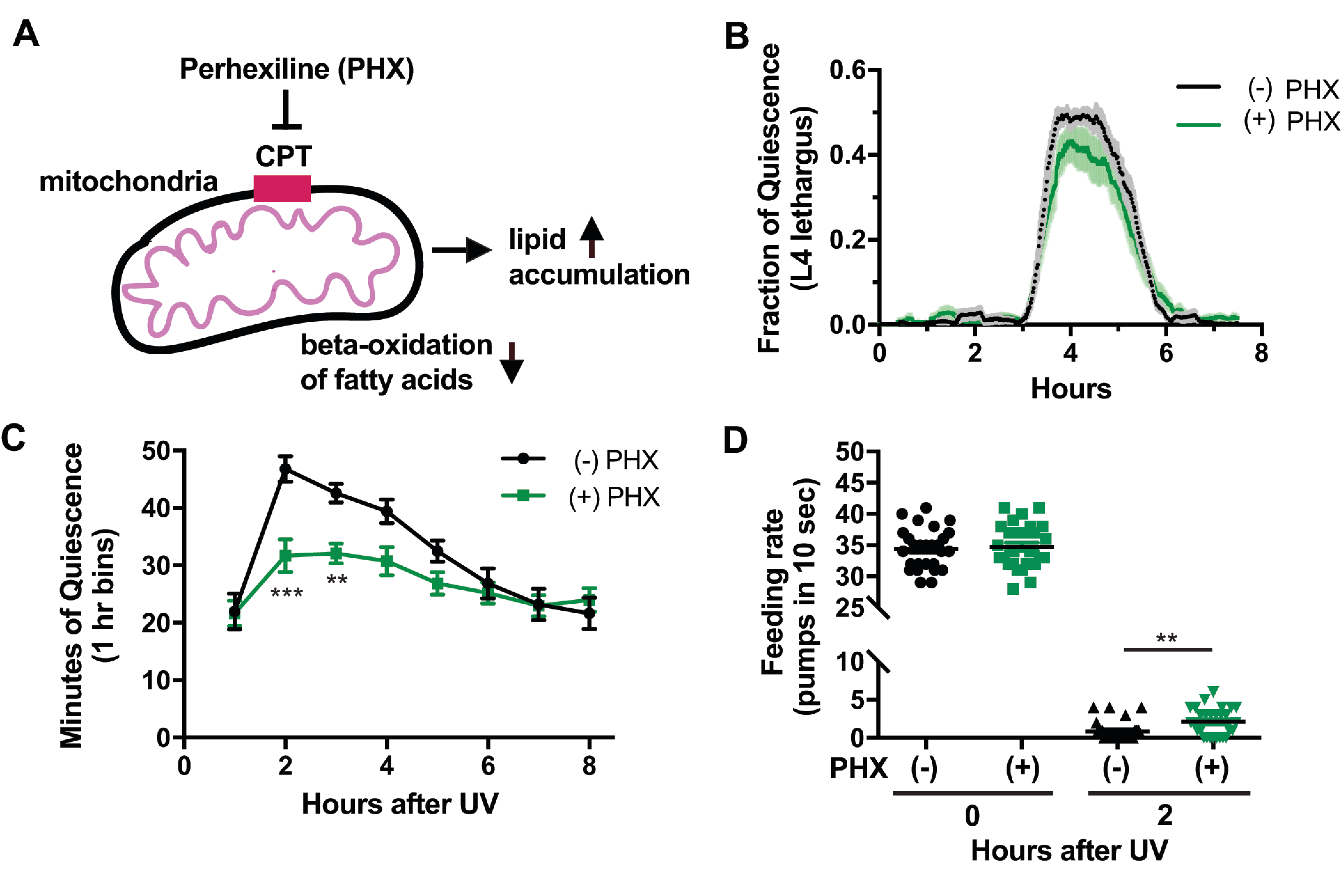

Supplement: S7 Fig — (A) Schematic showing that the CPT inhibitor PHX blocks beta-oxidation of fatty acids in mitochondria to promote the accumulation of lipids. (B) PHX (1 mM) reduces body movement quiescence during L4 lethargus/DTS in wild-type animals in comparison with vehicle controls (-PHX). Data are represented as a moving window of the fraction of a 10-min time interval spent quiescent of n = 9 animals for the PHX(-) trace and n = 11 animals for the PHX(+) trace. The x-axis represents hours from the start of recording in the late L4 stage. The data from individual worms were aligned such that the start of lethargus quiescence occurred simultaneously. Shading indicates SEM (S2 Data, Sheet S7A). (C and D) Time course of minutes of quiescence in 1-hr bins (C) and feeding rate (in pumps per 10 sec) (D) after UVC irradiation (1,500 J/m2) in the absence (-) and presence (+) of PHX. Data are represented as the mean ± SEM with n = 10 animals for movement quiescence (C) and n = 27–28 animals for feeding quiescence (D) for the PHX(-) and PHX(+) condition. Statistical comparisons were performed with a 2-way ANOVA using time and PHX conditions as factors, followed by post hoc pairwise comparisons at each time point to obtain nominal p-values, which were subjected to a Bonferroni correction for multiple comparisons. ** and *** indicate corrected p-values that are different from wild-type at p < 0.01, and p < 0.001, respectively (S2 Data, Sheet S7C and S7D). CPT, carnitine palmitoyltransferase; DTS, developmentally timed sleep; L4, fourth larval stage; PHX, perhexiline; SIS, stress-induced sleep; UVC, ultraviolet C. (TIF) [file pbio.3000220.s007.tif]

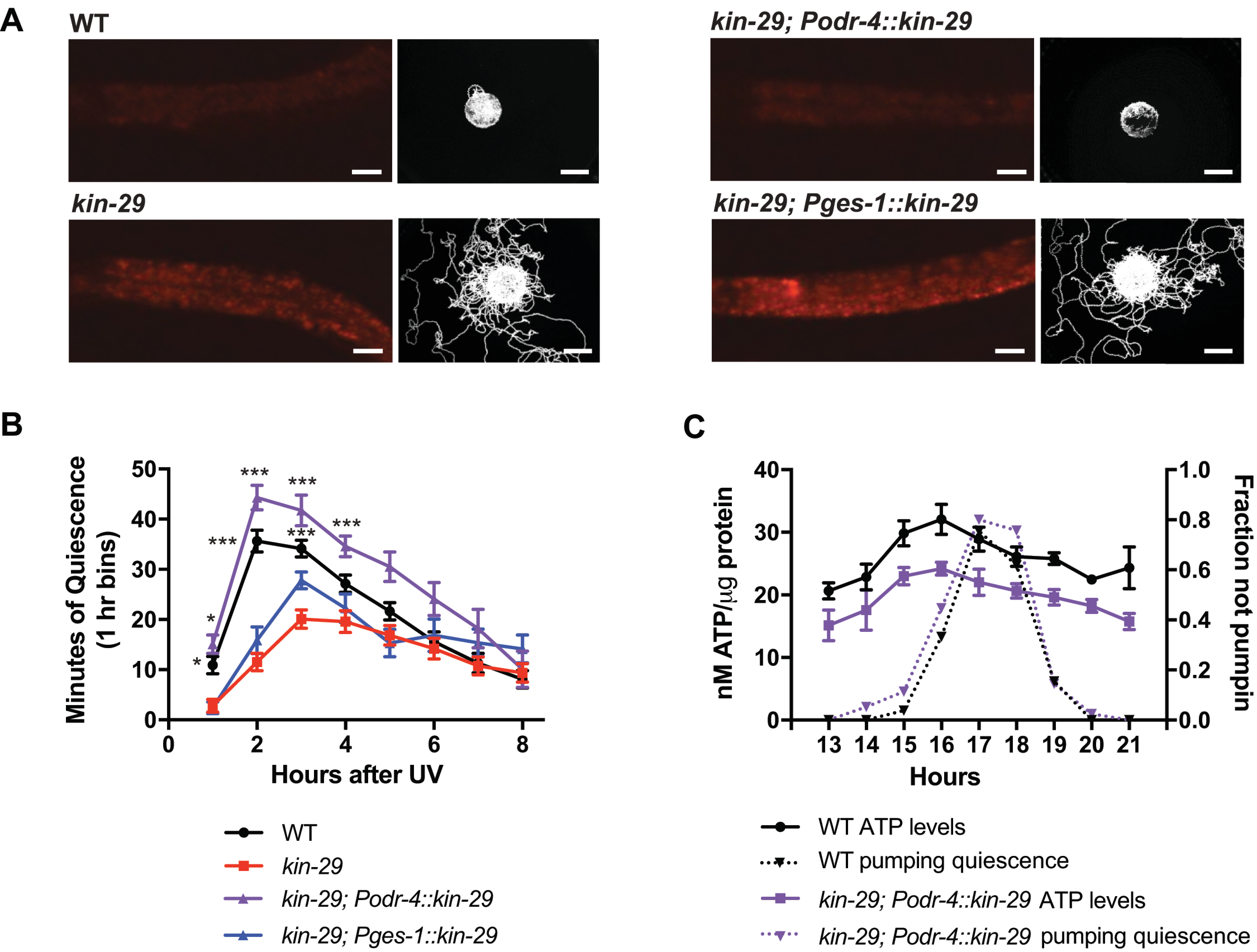

Supplement: S8 Fig — (A) Representative images of animals fixed and stained with Nile Red (left images, scale is 15 μm) and food-leaving behavior (black/white image, scale is 0.5 cm) of adult animals expressing the Podr-4::kin-29 or Pges-1::kin-29 transgene. odr-4, chemosensory promoter. ges-1, intestinal promoter. Frames from a 12-hr video were collapsed in a single image for food-leaving behavior. n = 7 animals in each image. (B) Time course of minutes of quiescence in 1-hr bins after UVC irradiation (1,500 J/m2). Data are represented as mean ± SEM (n = 14–32 animals). Statistical comparisons were performed with a 2-way ANOVA using time and genotype as factors, followed by post hoc pairwise comparisons at each time point to obtain nominal p-values, which were subjected to a Bonferroni correction for multiple comparisons. *** and * indicate corrected p-values that are different from kin-29 mutants at p < 0.001 and p < 0.05, respectively (S2 Data, Sheet S8B). (C) Levels of total body ATP levels normalized by μg protein in wild-type and kin-29 null mutant animals expressing the Podr-4::kin-29 transgene measured before, during, and after L1 lethargus/DTS. The graphs show the mean ± SD of 1 experiment for wild type with 2 technical replicates and 1 experiment for kin-29 animals with 3 technical replicates that carry the extrachromosomal Podr-4::kin-29 transgene (PY5791). Of note, PY5791 includes about 20% nontransgenic kin-29 animals. The second y-axis shows the averaged fraction of nonpumping animals (n = 10) for each genotype and time point. Animals expressing Podr-4::kin-29 are not significantly different from wild type, as determined by a 2-way ANOVA using time and genotype as factors, followed by a Bonferroni multiple-comparison test (S2 Data, Sheet S8C). DTS, developmentally timed sleep; ges-1, gut esterase 1; L1, first larval stage; UVC, ultraviolet C. (TIF) [file pbio.3000220.s008.tif]

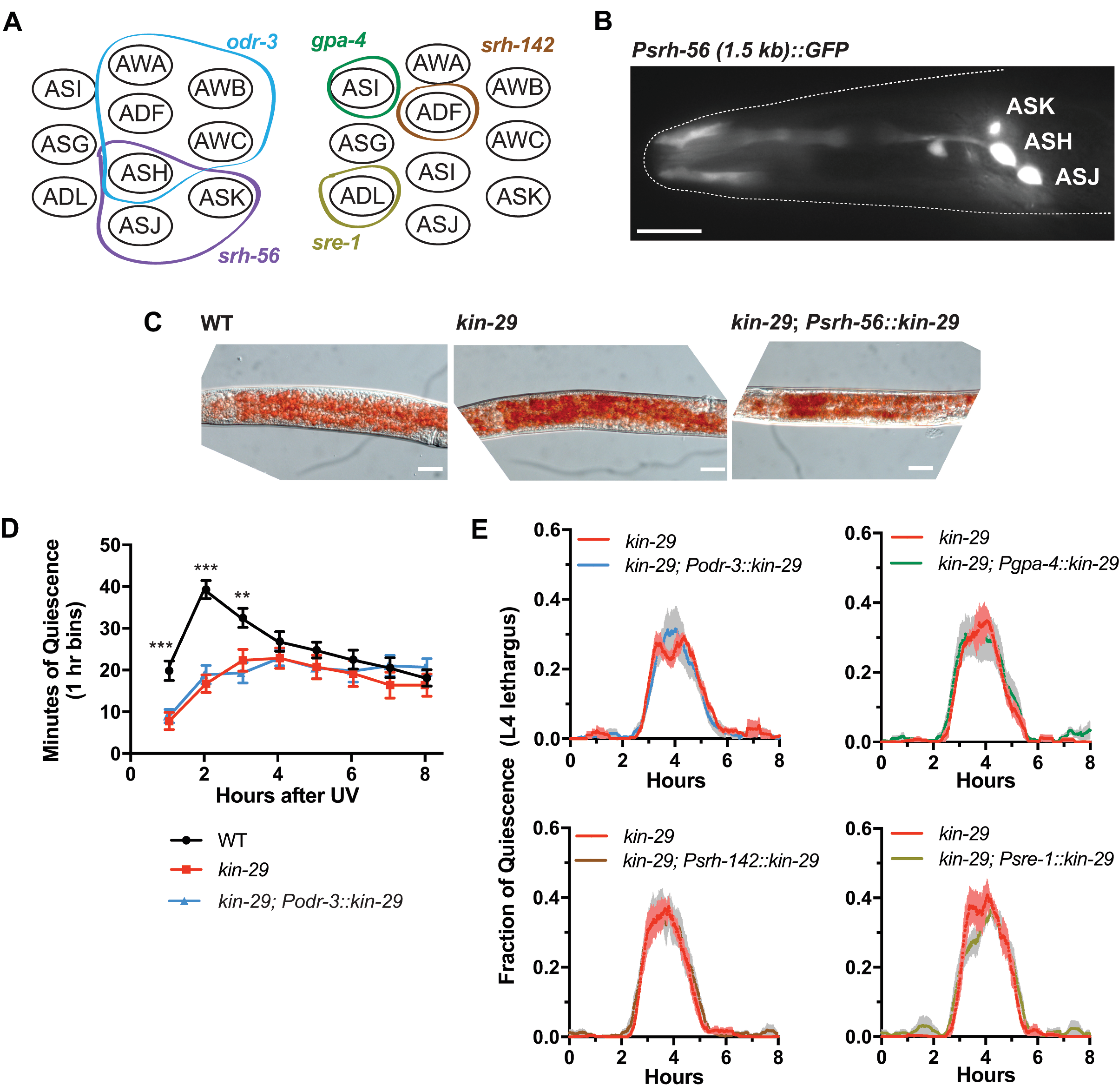

Supplement: S9 Fig — (A) Schematic showing the transgenic rescue strategy used to restore kin-29 expression in a subset of odr-4-expressing sensory neurons (left) and in individual neurons (right). (B) Expression and localization of a srh-56 promoter (approximately 1.5 kb) fusion with GFP. A black and white image shows Psrh-56::gfp fluorescence in ASK, ASH, and ASJ neurons plus other nonneuronal cells. Scale is 10 μm. (C) Representative images of a single L4 larvae fixed and stained with Oil Red O of wild-type and kin-29 null mutant animals with or without the Psrh-56::kin-29 transgene. Scale is 15 μm. (D) Time course of minutes of quiescence in 1-hr bins after UVC irradiation (1,500 J/m2) of wild type and kin-29 null mutants with or without the Podr-3::kin-29 transgene. Data are represented as the mean ± SEM (n = 14–15 animals). Statistical comparisons were performed with a mixed-effects analysis using time and genotype as factors, followed by post hoc pairwise comparisons at each time point to obtain nominal p-values, which were subjected to a Bonferroni correction for multiple comparisons. *** and ** indicate corrected p-values that are different from kin-29 mutants at p < 0.001 and p < 0.01, respectively (S2 Data, Sheet S9D). (E) Fraction of quiescence of kin-29 null mutants expressing kin-29 under control of the odr-3 (AWA, AWB, AWC, ADF, ASH), gpa-4 (ASI), srh-142 (ADF), and sre-1 (ADL) promoter. Data are represented in a 10-min time interval of n = 3–5 animals for each sleep trace. The x-axis represents hours from the start of recording in the late L4 stage. The data from individual worms were aligned such that the start of lethargus quiescence occurred simultaneously. Shading indicates SEM (S2 Data, Sheet S9E). GFP, green fluorescent protein; L4 stage, fourth larval stage; UVC, ultraviolet C. (TIF) [file pbio.3000220.s009.tif]

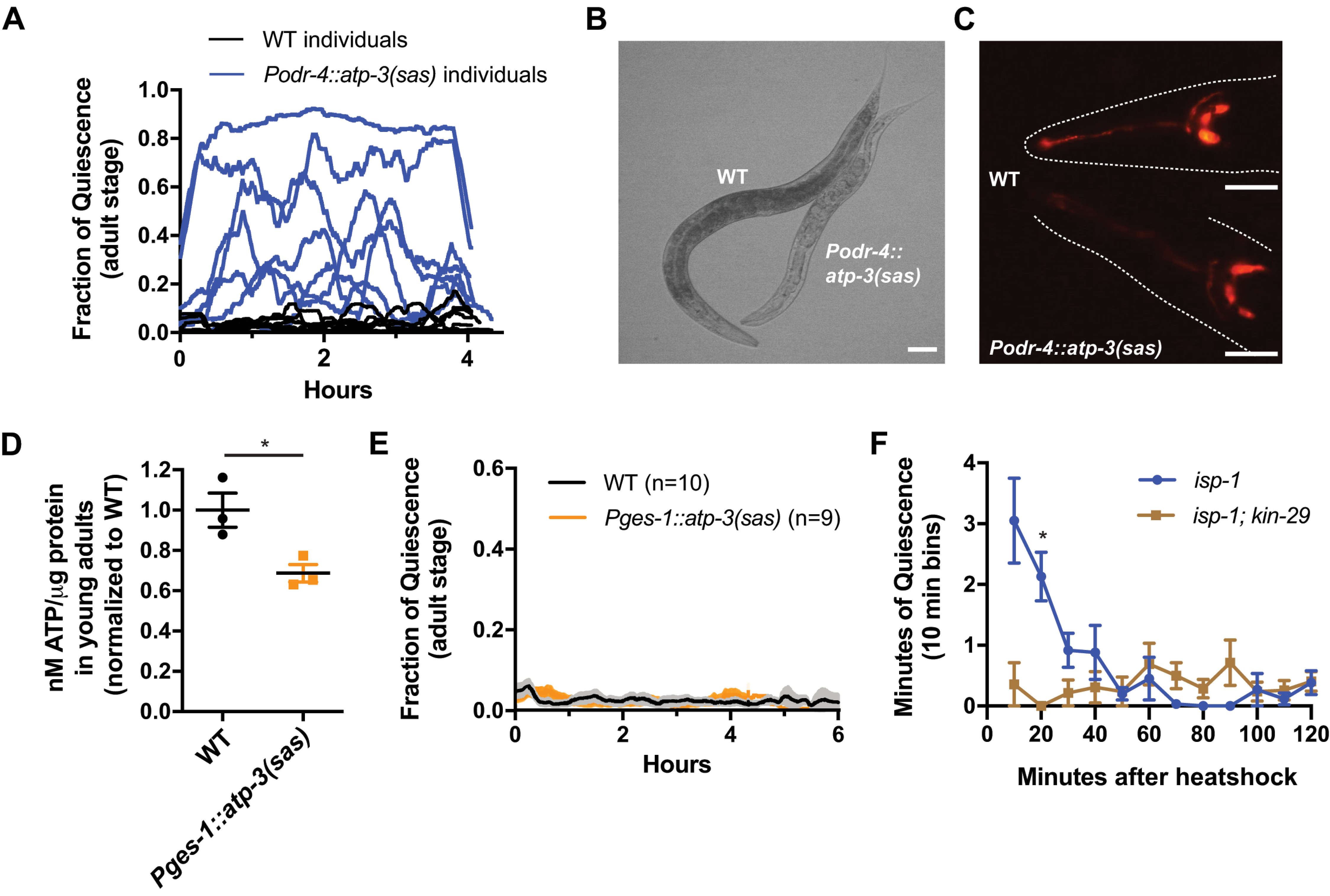

Supplement: S10 Fig — (A) Individual traces of the fraction of quiescence of Podr-4::atp-3(sas) animals and wild-type control animals. The fraction of quiescence in a 10-min moving window is shown for each trace (n = 7–9 animals). The x-axis represents hours from the start of recording at the young adult stage (S2 Data, Sheet S10A). (B) Representative image of an animal carrying the Podr-4::atp-3(sas) transgene, which has a reduced body size compared with a wild-type animal. Scale is 50 μm. (C) Representative images of dye-filling of sensory neurons of a wild-type and Podr-4::atp-3(sas) transgenic animal. Scale is 10 μm. (D) Levels of total body ATP normalized by μg protein measured in young adults of wild-type control animals and Pges-1::atp-3(sas) transgenic animals. Data are normalized to wild-type controls and are represented as the mean ± SEM of 3 experiments. *p < 0.05 by an unpaired t test (S2 Data, Sheet S10D). (E) Averaged traces of the fraction of quiescence of Pges-1::atp-3(sas) animals (n = 9) and wild-type control animals (n = 10). Data are represented as a moving window of the fraction of a 10-min time interval spent quiescent for each genotype. The x-axis represents hours from the start of recording at the young adult stage. Shading indicates SEM (S2 Data, Sheet S10E). (F) isp-1 single mutants are highly quiescent compared with isp-1; kin-29 double-mutant animals after heat shock/SIS. Adult animals were heat-shocked at 35°C for 20 min. Minutes of body movement quiescence of isp-1 (n = 10) and isp-1; kin-29-mutant animals (n = 7). Shading indicates SEM. *p < 0.05 by a 2-way ANOVA with Bonferroni’s multiple-comparisons test (S2 Data, Sheet S10F).; RNAi, RNA interference; sas, sense and antisense. (TIF) [file pbio.3000220.s010.tif]

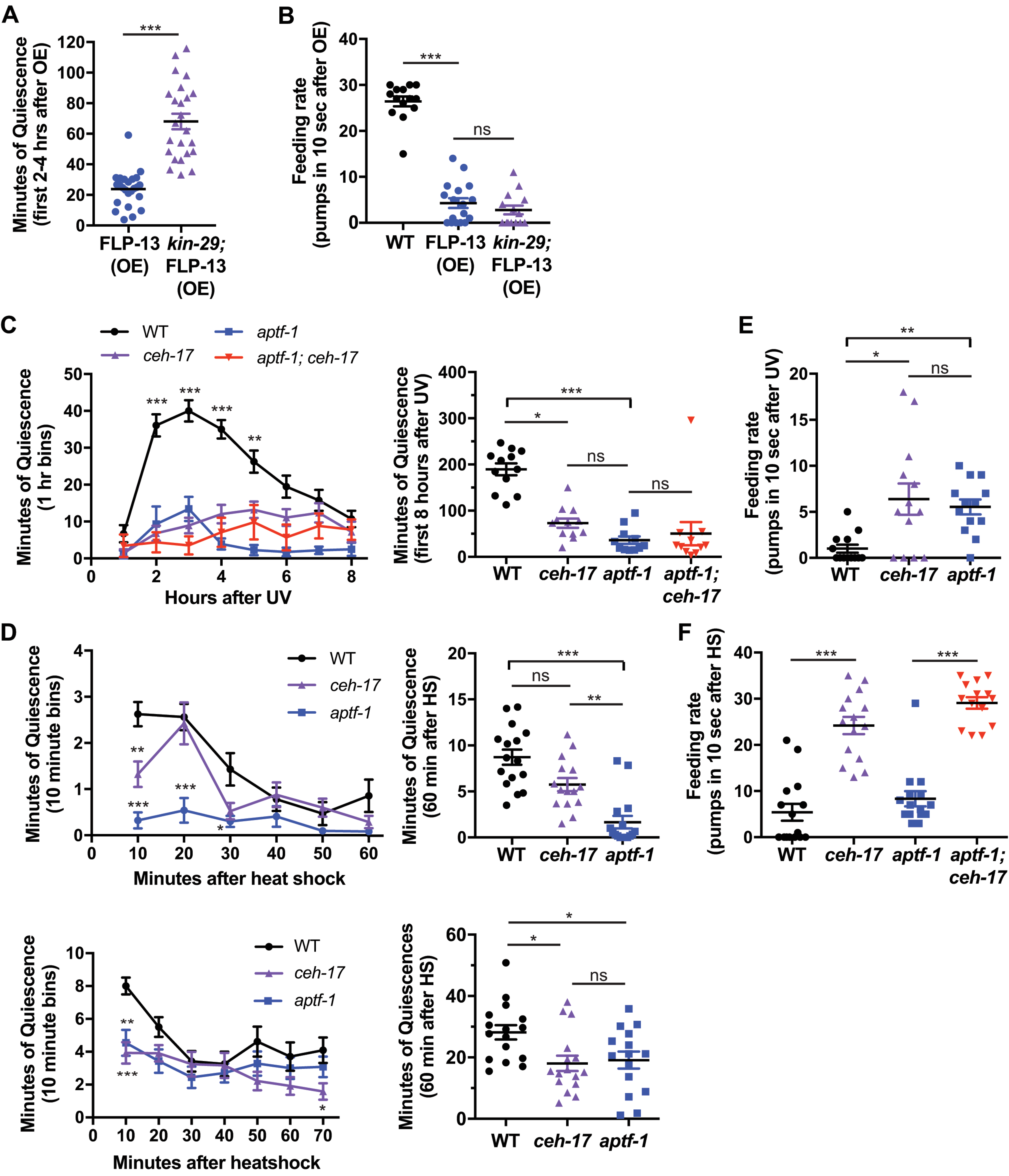

Supplement: S11 Fig — (A and B) Body movement quiescence (A) and feeding rate (B) of kin-29 null mutants after induction of FLP-13 OE. To induce FLP-13 OE, adult animals expressing a Phsp-16.2::FLP-13 transgene were heat-shocked for 30 min (see Material and methods). Data are represented as the mean ± SEM with n = 24 animals for movement quiescence (A) and n = 14–17 animals for feeding quiescence. Feeding rate in pumps per 10 s was determined 2 hr after heat shock. ns and ***p < 0.001 by a 2-tailed Mann-Whitney t test (A) and an ANOVA with Tukey multiple-comparisons test (B) (S2 Data, Sheet S11A and S11B). (C and D) Body movement quiescence following either UVC irradiation 1,500 J/m2 (C) or heat shock at 35°C for 30 min (D). Data are represented as mean ± SEM (n > 10 animals for each genotype). Left graphs: For the time-course experiments, statistical comparisons were performed with a 2-way ANOVA using time and genotype as factors, followed by post hoc pairwise comparisons at each time point to obtain nominal p-values, which were subjected to a Bonferroni correction for multiple comparisons. ***, **, and * indicate corrected p-values that are different from wild type at p < 0.001, p < 0.01, and p < 0.05, respectively. Right graphs: Total minutes of movement quiescence during 8 hr after UVC irradiation (C) and 1 hr after heat shock (D) determined from the time-course data. Data are represented as mean ± SEM. ns, ***p < 0.001, **p < 0.01, and *p < 0.05 by a Kruskal-Wallis with Dunn multiple-comparisons test. Lower 2 graphs of S11D Fig: Replication experiment of movement quiescence following heat shock at 35°C for 30 min of ceh-17- and aptf-1-mutant animals compared with wild type. Left panel: 2-way ANOVA with Bonferroni correction for multiple comparisons. Right panel: 1-way ANOVA with Tukey multiple comparisons; ***p < 0.001, **p < 0.01, *p < 0.05, and ns (S2 Data, Sheet S11C and S11D). (E and F) Feeding rate following either UVC irradiation 1,500 J/m2 (E) or heat shock at 35°C for 30 min [file pbio.3000220.s011.tif]

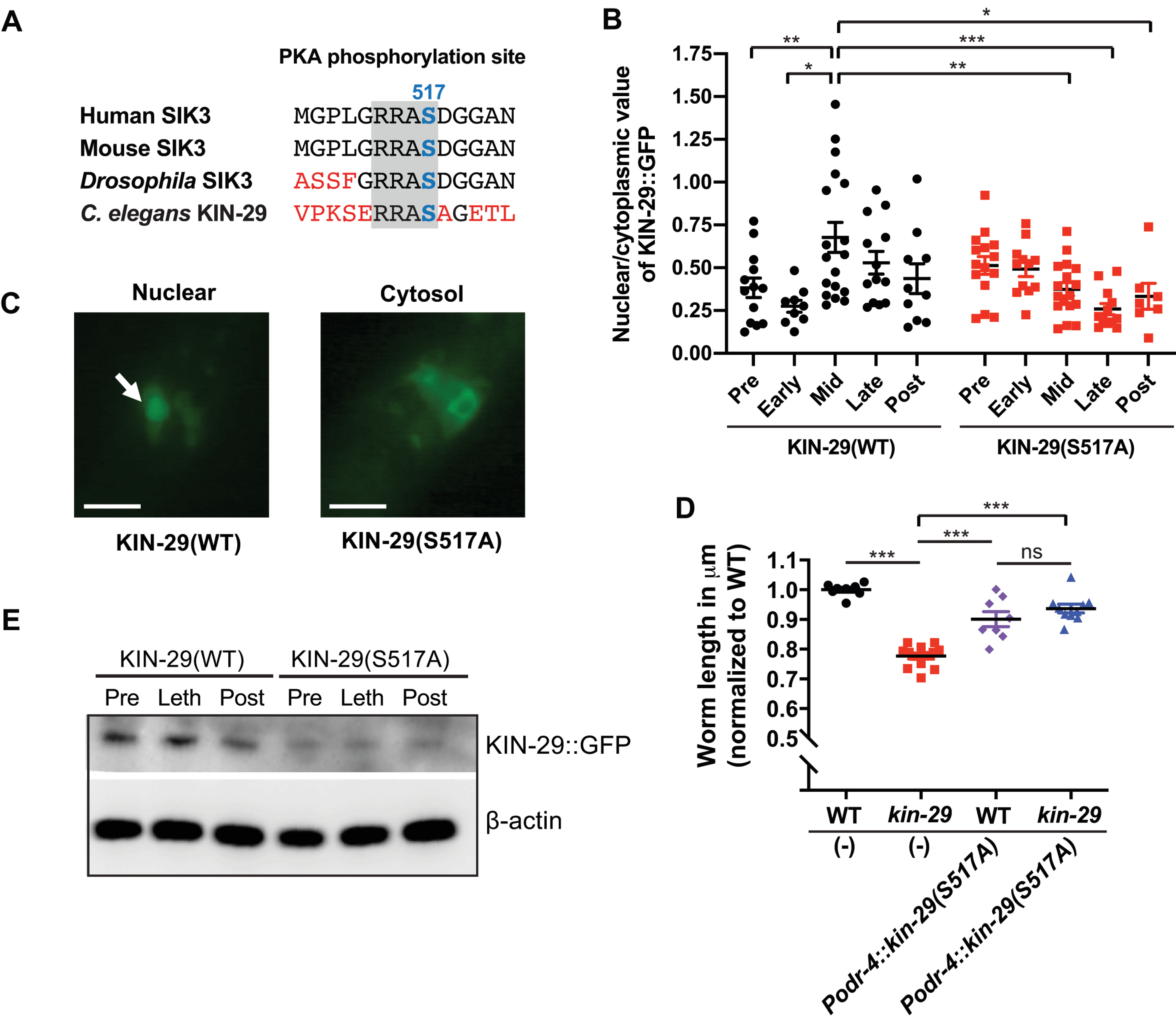

Supplement: S12 Fig — (A) Sequence alignment of SIK3 proteins (human Q9Y2K2, mouse Q6P4S6, Drosophila Q4QQA7, C. elegans Q21017). The conserved PKA phosphorylation site serine is shown in blue. (B) Quantification of KIN-29(WT)::GFP and KIN-29(S517A)::GFP in a subcellular compartment of odr-4(+) neurons before, during, and after lethargus/DTS of the L1 stage. Higher values are indicative of greater nuclear localization of the transgene, whereas lower values are indicative of greater cytosolic localization of the transgene (see Material and methods). Data are represented as the mean ± SEM. **p < 0.01 and *p < 0.05 by an ANOVA with Tukey multiple-comparisons test (S2 Data, Sheet S12B). (C) KIN-29(S517A) remains cytosolic after heat shock. Shown is KIN-29(WT)::GFP in the nucleus (arrow) of an odr-4-expressed neuron after heat shock (left image). KIN-29(S517A)::GFP remains in cytoplasmic after heat shock (right image). Scale is 10 μm. (D) KIN-29(S517A) rescues the small body size of kin-29 null mutants. Shown is the relative body length of adult animals (n = 8–13) of the indicated genotypes. Data are represented as the mean ± SEM. ns and ***p < 0.001 by an ANOVA with Tukey multiple-comparisons test (S2 Data, Sheet S12D). (E) Western blot analysis of KIN-29::GFP in Podr-4::kin-29(WT)::GFP and Podr-4::kin-29(S517A)::GFP transgenic strains before, during, and after lethargus/DTS of the L1 stage (S2 Data, Sheet S12E). KIN-29::GFP levels were overall lower in kin-29(S517A)::GFP, as evident both by lower gfp fluorescence in this strain and reduced anti-GFP staining. This difference may be explained by different copy numbers of the transgene or by different stability of KIN-29(S517A) in comparison with KIN-29(WT). Levels of KIN-29::GFP protein (top panel) using anti-GFP antibodies are indicated with β-actin (lower panel) as a loading control. DTS, developmentally timed sleep; GFP, green fluorescent protein; KIN-29(WT), wild-type KIN-29; L1 stage, first larval stage; ns, not significant; PKA, Protein [file pbio.3000220.s012.tif]

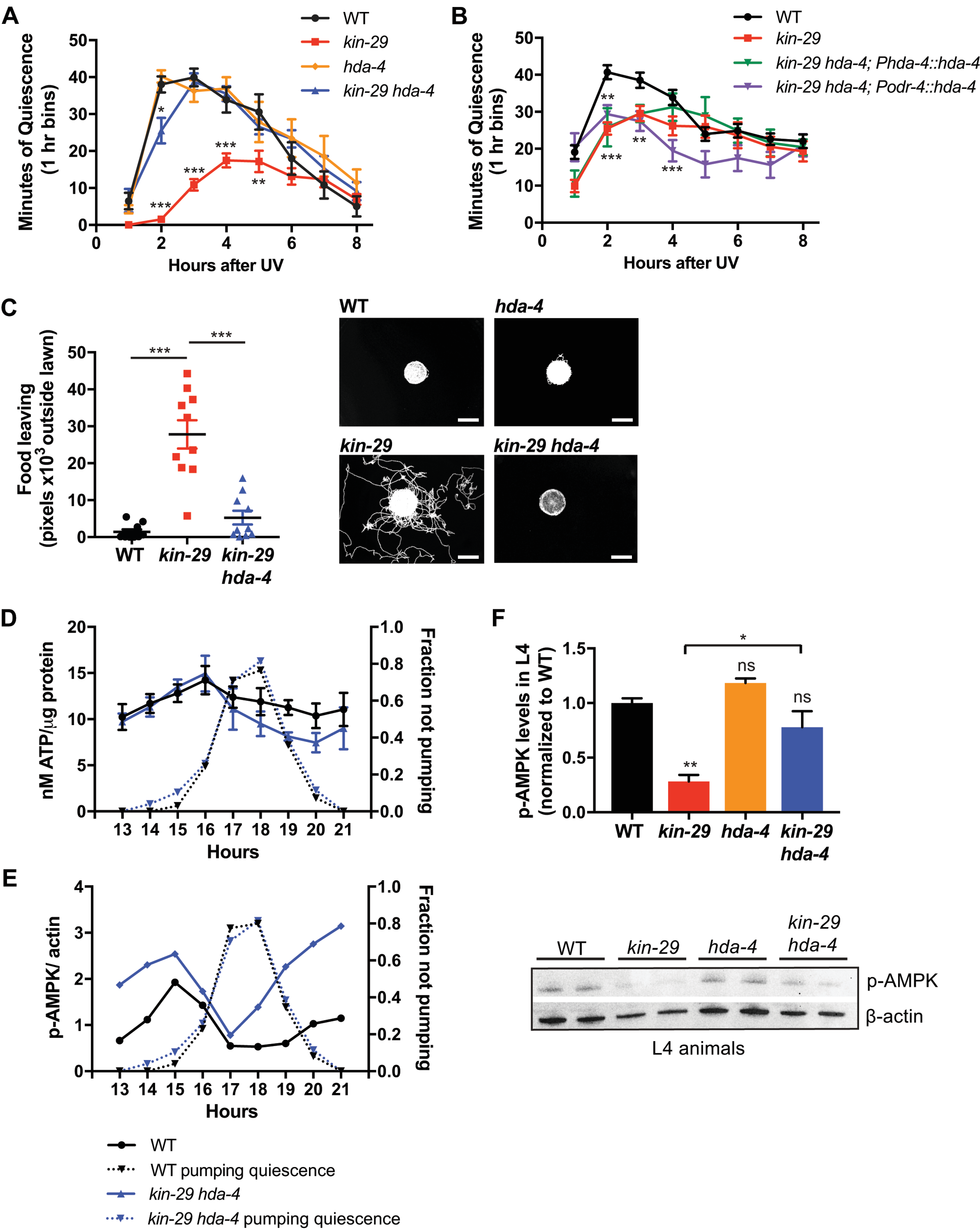

Supplement: S13 Fig — (A) Time course of minutes of quiescence in 1-hr bins after UVC irradiation (1,500 J/m2) of single and double mutants between kin-29 and hda-4 compared with wild type. Data are represented as the mean ± SEM (n > 10 animals). Statistical comparisons were performed with a 2-way ANOVA using time and genotype as factors, followed by post hoc pairwise comparisons at each time point to obtain nominal p-values, which were subjected to a Bonferroni correction for multiple comparisons. ***, **, and * indicate corrected p-values that are different from wild type at p < 0.001, p < 0.01, and p < 0.05, respectively (S2 Data, Sheet S13A). (B) Time course of minutes of quiescence in 1-hr bins after UVC irradiation (1,500 J/m2) of animals expressing hda-4 under the control of its own promoter and under the control of the odr-4 chemosensory neuron specific promoter. Data are represented as the mean ± SEM (n > 10 animals). Statistical comparisons were performed with a 2-way ANOVA using time and genotype as factors, followed by post hoc pairwise comparisons at each time point to obtain nominal p-values, which were subjected to a Bonferroni correction for multiple comparisons *** and ** indicate corrected p-values that are different from wild type at p < 0.001 and p < 0.01, respectively (S2 Data, Sheet S13B). (C) Food-leaving behavior of wild-type, single-mutant, and double-mutant animals of kin-29 and hda-4 quantified as the area of exploration, with each data point representing tracks from a population outside the bacterial lawn. Each data point represents the total number of pixels outside of the bacterial lawn of 7 animals per plate, and the horizontal line represents the mean ± SEM of individual experiments. Right images: Frames from a 12-hr video were collapsed in a single image for food-leaving behavior. Scale is 0.5 cm. *** indicates values that are different from wild type, kin-29, or hda-4 single mutants at p < 0.001 by an ANOVA with Tukey multiple-comparisons test (S2 Data, [file pbio.3000220.s013.tif]

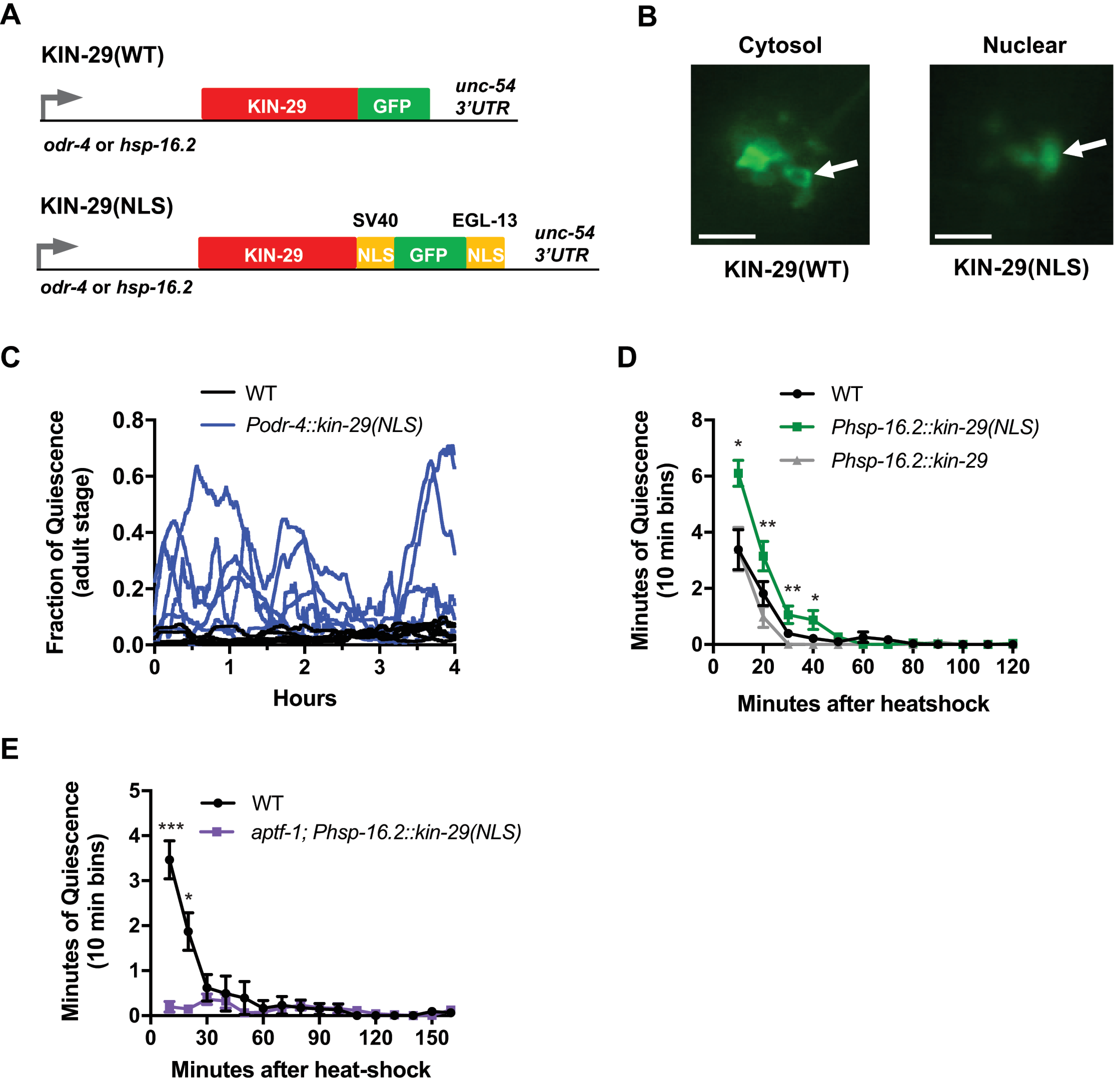

Supplement: S14 Fig — (A) Schematic of the generated KIN-29(NLS)::GFP construct (see Material and methods) for the constitutive expression of KIN-29 in the nuclei of odr-4-expressing sensory neurons, or under the control of the hsp-16.2 promoter. KIN-29(WT) is the control construct without NLS. (B) Representative images of KIN-29(WT)::GFP in the cytosol (left image) and KIN-29(NLS)::GFP in the nucleus (right image) of odr-4-expressed sensory neurons. The arrow indicates a cell of the odr-4(+) neurons. Scale is 10 μm. (C) Individual traces of the fraction of quiescence of Podr-4::kin-29(NLS)::GFP transgenic animals and wild-type controls animals. The fraction of quiescence in a 10-min moving window is shown for each trace over a 4-hr period. The x-axis represents hours from the start of recording at the young adult stage (S2 Data, Sheet S14C). (D) Body movement quiescence is increased in Phsp-16.2::kin-29(NLS) transgenic animals after heat shock/SIS in comparison with control wild-type and hsp::kin-29 transgenic animals. Adult animals were heat-shocked at 35°C for 20 min. Graphs show the mean ± SEM of wild-type (n = 20), Phsp-16.2::kin-29(NLS) (n = 10), and Phsp-16.2::kin-29 (n = 22) animals for movement quiescence. Statistical comparisons were performed with a mixed-effects analysis using time and genotype as factors, followed by post hoc pairwise comparisons at each time point to obtain nominal p-values, which were subjected to a Tukey multiple-comparisons test. ** and * indicate corrected p-values that are different from Phsp-16.2::kin-29 expressing animals at p < 0.01 and p < 0.05, respectively (S2 Data, Sheet S14D). (E) Mutations in aptf-1 suppress the increased movement quiescence of animals expressing Phsp-16.2::kin-29(NLS) after heat shock/SIS in comparison with wild-type control. Adult animals were heat-shocked at 35°C for 20 min. Minutes of body movement quiescence of wild-type (n = 18) and aptf-1; Phsp-16.2::kin-29(NLS) (n = 16) animals. Shading indicates SEM. ***p < 0.001 and [file pbio.3000220.s014.tif]
